# A Titanium‐Catalyzed Reductive α‐Desulfonylation

**DOI:** 10.1002/chem.202005400

**Published:** 2021-03-05

**Authors:** Christoph Kern, Jan Selau, Jan Streuff

**Affiliations:** ^1^ Institut für Organische Chemie Albert-Ludwigs-Universität Freiburg Albertstr. 21 79104 Freiburg im Breisgau Germany

**Keywords:** catalysis, defunctionalization, radicals, sulfones, titanium

## Abstract

A titanium(III)‐catalyzed desulfonylation gives access to functionalized alkyl nitrile building blocks from α‐sulfonyl nitriles, circumventing traditional base‐mediated α‐alkylation conditions and strong single electron donors. The reaction tolerates numerous functional groups including free alcohols, esters, amides, and it can be applied also to the α‐desulfonylation of ketones. In addition, a one‐pot desulfonylative alkylation is demonstrated. Preliminary mechanistic studies indicate a catalyst‐dependent mechanism involving a homolytic C−S cleavage.

## Graphical abstract

**Catalyst‐controlled cleavage**: A transition‐metal‐catalyzed desulfonylation gives access to functionalized nitriles and ketones from readily‐available α‐sulfonylated precursors. The homolytic reductive C−S cleavage occurs under the control of an in situ generated titanium(III) catalyst and it can be combined with a Michael addition reaction to achieve a one‐pot desulfonylative alkylation.

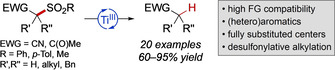

Sulfonyl groups provide a versatile platform for organic chemistry.^[1]^ In combination with a subsequent desulfonylation, they are often used as traceless linchpins for the construction of molecular frameworks.^[2]^ Here, several transition‐metal‐catalyzed and free‐radical‐based desulfonylative cross‐couplings have been developed.[^3^, ^4^] The reductive removal of sulfonyl groups at aliphatic carbon centers, however, requires strong single‐electron‐donating reagents such as Na(Hg), Al(Hg), Mg‐MeOH, SmI_2_, TiCl_4_‐Zn, but it becomes facilitated at benzylic and allylic centers, and at α‐positions of electron‐withdrawing groups.^[2]^ The reductive α‐desulfonylation of carbonyls and nitriles can be achieved with SmI_2_, under free radical conditions using tin hydrides, or with magnesium in small alcoholic solvents, for example.[^2^, ^5^] Photochemical protocols have been reported as well and for particularly activated substrates even zinc in acidic media can be sufficient to achieve the C−S cleavage.[^2e^, ^6^, ^7^] A transition metal catalyzed reductive desulfonylation that complements these reactions, on the other hand, has remained desirable.

Titanium(III) catalysis is a modern tool for organic synthesis.[^8^, ^9^, ^10^] As part of our interest in catalytic reductive functional group cleavage reactions, we have recently developed a mild titanium(III)‐catalyzed decyanation of geminal dinitriles (Scheme 1).[^11^, ^12^] Herein, we report the realization of a corresponding desulfonylation by titanium(III) single electron transfer catalysis that is based on a non‐optimized example from this earlier work. Moreover, a first application towards a desulfonylative alkylation reaction is explored.

**Scheme 1:**
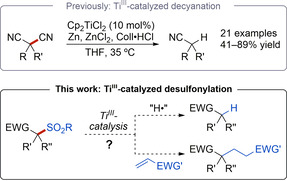
Desulfonylations and desulfonylative couplings.

The investigations started with the desulfonylation of 3‐phenyl‐2‐(phenylsulfonyl)propionitrile (**1 a**) using the previous conditions:^[11]^ in presence of Cp_2_TiCl_2_ as catalyst, zinc, and the additives ZnCl_2_, Coll⋅HCl, and TMSCl, a clean desulfonylation to 3‐phenylprionitrile (**2 a**) occurred in 56 % yield (Coll=2,4,6‐collidine). Since thiophenol was produced as byproduct, three equivalents of zinc were added to account for the sulfonyl reduction and the turnover‐enabling catalyst reduction. In the following optimization studies, it was possible to further improve the conditions in order to achieve the desired C−S cleavage in a clean fashion and with maximized yield (Table 1).^[13]^ A concentration of *c=*0.25 m proved to be ideal and an interesting dependence of the yield on the catalyst properties, the solvent, and the reaction temperature was observed. In THF at 60 °C the highest yield was achieved with (EtCp)_2_TiCl_2_ and catalysts with a significantly stronger reduction potential and increased steric bulk such as *rac*‐(ebthi)TiCl_2_ and Cp*2 TiCl_2_ gave inferior results. Changing the solvent to toluene and raising the temperature to 110 °C inverted this trend and Cp*2 TiCl_2_ gave the highest yield (96 %). It is noteworthy that all additives were required to achieve this high yield. No reaction was observed in absence of the titanium catalyst (THF, 60 °C and toluene, 110 °C).^[13]^ This is important, because 1) Zn‐TMSCl mixtures have been reported to promote radical reactions by single electron transfer at elevated temperature,^[14]^ and 2) Zn‐mediated desulfonylations at activated positions have literature precedence.^[7]^ Lowering the catalyst amount to 5 mol % reduced the yield to 43 %.^[13]^

**Table 1:** Summary of the reaction optimization.^[a]^

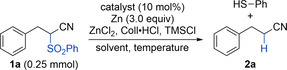			
Catalyst	ERedP/2 [V]^[b]^	THF, 60 °C	Toluene, 110 °C
Cp_2_TiCl_2_	−1.31	56 %^[c]^	41 %
(EtCp)_2_TiCl_2_	−1.40	66 %	60 %
*rac*‐(ebthi)TiCl_2_	−1.55	24 %	68 %
Cp*2 TiCl_2_	−1.65	20 %	**96 %** ^[d]^
none	–	trace^[e]^	trace^[e]^

[a] Conditions: catalyst (10 mol %), Zn (3.0 equiv), ZnCl_2_ (1.0 equiv), Coll⋅HCl (2.0 equiv), TMSCl (3.0 equiv), solvent (*c=*0.25 m), *t=*48 h. Percent values are yields of isolated **2 a**. [b] Half‐wave reduction potential determined by cyclic voltammetry of 2 mm catalyst solutions in 0.2 m Bu_4_NPF_6_/THF against the Fc^+^/Fc couple. [c] From Ref. [11]. [d] 24 h reaction time. [e] 1–3 % as determined by NMR analysis of the crude reaction mixture. ebthi=ethylenebis(*η* ^5^‐4,5,6,7‐tetrahydroindenyl).

The effectiveness of the catalytic desulfonylation was demonstrated on a range of substrates bearing common functional groups on 0.5 mmol scale (Scheme 2). Methylated and chlorinated aromatics as well as aromatic ester and phenolic OH groups were well‐tolerated (**2 b**–**2 e**, 75–87 % yield). The phenol was converted into the corresponding TMS ether under the reaction conditions and then liberated again by acidic workup. A *p*‐acetoxyphenyl‐substituted precursor gave 60 % of nitrile **2 f**, 13 % of **2 e**, and *S*‐phenyl thioacetate, which could be separated. Here, a minor transesterification of the thiophenol byproduct with the aryl ester group had occurred. Amide **1 g**, on the other hand, exclusively gave **2 g** in 78 % yield. Bis‐methoxylated and fluorinated aromatics were desulfonylated into **2 h** and **2 i** in 70 % and 81 % yield, respectively. Naphthyl as well as thienyl groups were unproblematic too (**2 j** and **2 k**). The desulfonylation also proceeded smoothly on fully substituted tertiary carbon centers (**2 l**, 86 %), on a range of purely aliphatic α‐sulfonylated nitriles giving **2 m–2 o** (61–82 %), and on a tertiary substrate with an additional cyanoalkyl substituent (**2 p**, 66 %). The desulfonylations of **1 l**–**n** were carried out on a 1 mmol scale and the reaction with **1 o** on a 3 mmol scale. In the case of the latter, this facilitated the isolation of **2 o**. Importantly, the reaction could also be applied to the α‐desulfonylation of a ketone as was exemplified on **1 q** giving **2 q** in 76 % yield.^[6a]^ Here, a slow background reaction was observed that produced 16 % of ketone **2 q** when no titanium catalyst was present. Interestingly, a corresponding α‐phenylsulfonyl ester showed no reaction. For completion, it should be noted that a desulfonylation at a benzylic position showed a more significant background reaction (**2 r**). Still, the addition of the catalyst resulted in a substantial improvement of the outcome (89 % vs. 44 % uncatalyzed). Besides phenylsulfonyl the common *p*‐toluenesulfonyl and methanesulfonyl groups could be removed as is shown for **3** and **4** that were converted into **2 a** in 90 % and 95 % yield, respectively (Scheme 3). As for most examples in Scheme 2, these were “spot‐to‐spot” reactions.

**Scheme 2:**
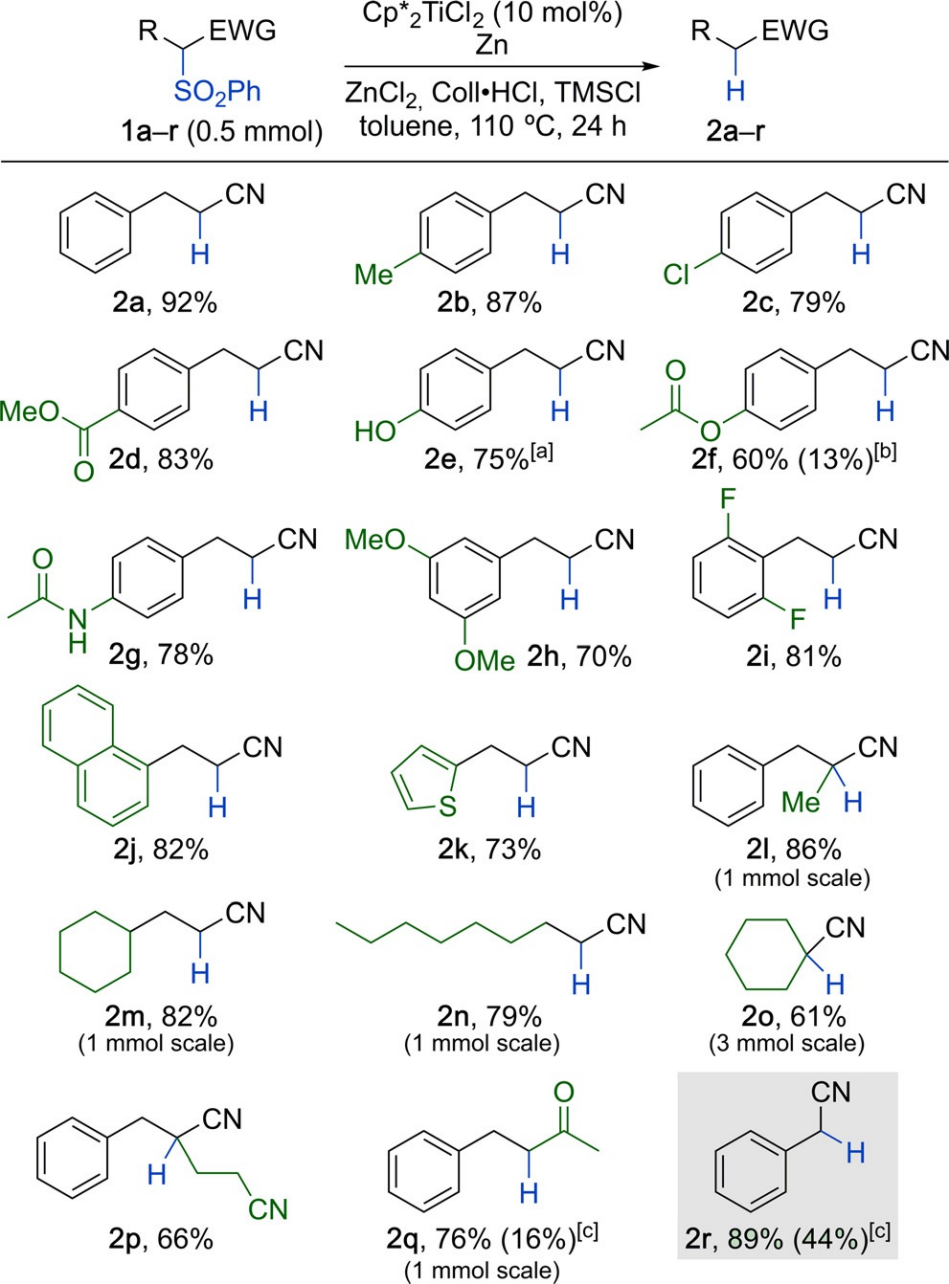
Scope of the desulfonylation using the optimized conditions from Table 1 on a 0.5 mmol scale if not noted otherwise. [a] Workup with aq. HCl. [b] Brackets show the isolated amount of **2 e**. [c] Brackets show the result in absence of the titanium catalyst.

**Scheme 3:**
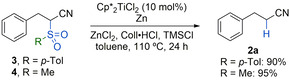
Catalytic removal of tosyl and mesyl groups.

We then sought to demonstrate, in form of a proof‐of‐principle, the further advancement of this desulfonylation into a desulfonylative alkylation by combining it with a Michael addition. This was supported by a number of titanium(III)‐catalyzed cross‐couplings involving Michael acceptors that had been developed earlier in our group.[^8a^, ^15^] In initial test reactions, acrylonitrile was identified as a suitable acceptor olefin and, after optimization of the reaction conditions, the desulfonylative coupling to dinitrile **2 p** was achieved in satisfying 60 % yield with only 5 mol % of catalyst at 80 °C (Scheme 4).^[13]^ Only traces of **2 a** were observed in the crude reaction mixture. Independent experiments showed that **1 a** and acrylonitrile underwent premature Michael addition in absence of the catalyst. It was concluded that the cross‐coupling proceeded via the Michael addition of **1 a** to acrylonitrile followed by the titanium‐catalyzed desulfonylation reaction.

**Scheme 4:**
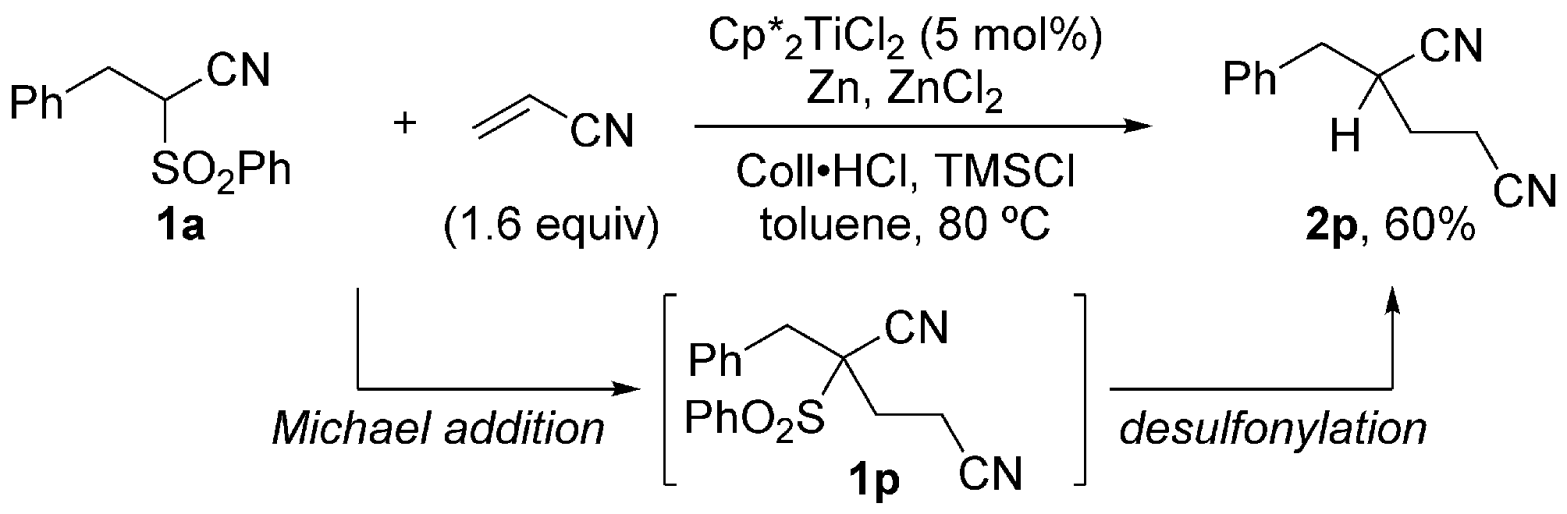
Desulfonylative one‐pot coupling with acrylonitrile.

To gain insight into the mechanism of the desulfonylation, thioether **5** was submitted to the reaction without titanium catalyst. A rapid C−S cleavage occurred and nitrile **2 a** was obtained in 78 % yield after 3 hours (Scheme 5 a). In a second experiment, **1 a** was reacted with a stoichiometric amount of pregenerated Cp*2 TiCl (with the excess of zinc removed by filtration) and nitrile **2 a** was formed in 30 % yield (33 % conversion) (Scheme 5 b). This exceeded the maximum possible yield (20 %) of a titanium(III)‐mediated cleavage pathway via thioether **5**. Furthermore, neither thioether **5** nor the corresponding sulfoxide were observed in our stoichiometric and catalytic experiments. However, the result indicated that additional titanium(III)‐mediated reduction steps had occurred, preventing a quantitative conversion of **1 a** into **2 a**.

**Scheme 5:**
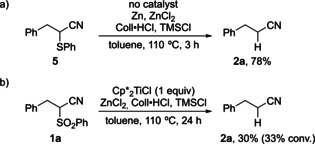
a) Non‐catalyzed α‐desulfenylation. b) Stoichiometric desulfonylation experiment.

We hypothesized that the condition‐dependent change in catalyst reactivity observed during the optimization studies (see Table 1) originated from a change in the mechanism. With Cp_2_TiCl_2_ a dual activation mode, as was established for the related titanium(III)‐catalyzed decyanation, was considered (Scheme 6 a).^[11]^ This circumvented the formation of free radicals and allowed the C−S cleavage to already take place at 60 °C. In contrast, the sulfone would not readily coordinate to a bulkier catalyst like Cp*2 TiCl_2_. An alternative single site activation would be the result, leading to the formation of free sulfonyl radicals. This path required a higher temperature and a stronger catalyst reduction potential. Preliminary calculations using the ORCA program package revealed that, in the case of Cp_2_Ti^III^Cl, coordination of the nitrile to the titanium center was slightly exergonic while coordination of the sulfone moiety was slightly endergonic under equilibrium conditions (Scheme 6 b and Table 2).[^13^, ^16^] With Cp*2 Ti^III^Cl the difference in coordination behavior changed dramatically and sulfone coordination became disfavored by 13.1 kcal mol^−1^. Changing the solvent from THF to toluene had only little effect, but the formation of **6‐Cp** became less exergonic in toluene (+0.3 kcal mol^−1^) while the formation of 6‐Cp* became more favorable (−1.4 kcal mol^−1^). Overall these observations were in agreement with the postulated change from a dual activation to a single activation mode. However, further studies will be required to completely rule out a dual activation.

**Scheme 6:**
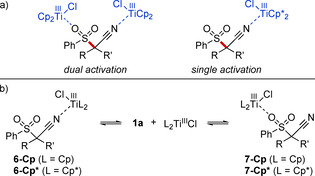
a) Catalyst‐dependent dual and single activation modes. b) Nitrile versus sulfone coordination to Cp_2_Ti^III^Cl and Cp*2 Ti^III^Cl.

**Table 2:** Calculated relative Gibbs Free Energies in kcal mol^−1^ for the coordination equilibria shown in Scheme 6 b.^[a]^

Species	in THF	in toluene
**1 a**+L_2_Ti^III^Cl	0	0
**6‐Cp**	−1.4	−1.1
**7‐Cp**	1.9	1.1
6‐Cp*	1.5	0.1
7‐Cp*	13.1	10.9

[a] Calculated on the PW6B95‐D3(BJ)‐CPCM/def2‐QZVP//TPSS‐D3(BJ)‐CPCM/def2‐TZVP level. Optimizations were carried out in THF and toluene separately.

A mechanism is proposed that starts with the reduction of the titanium(IV) catalyst to a titanium(III) species (Scheme 7). Then, coordination of the substrate via the cyano group follows and a single‐electron transfer (SET) from the titanium(III) center takes place, which leads to the *catalyst‐controlled* homolytic scission of the C−S bond.^[17]^ This single‐catalyst‐induced SET cleavage is facilitated with the bulkier and more electron‐rich Cp*2 TiCl_2_ catalyst and releases a free sulfonyl radical together with a ketenimidotitanium(IV) complex.^[18]^ The sulfonyl radical gets further reduced to the corresponding thiol and protonation of the ketenimidotitanium(IV) intermediate by Coll⋅HCl releases the nitrile product, regenerating the titanium(IV) catalyst. Although the roles of the additives still need further investigation, our observations indicate that ZnCl_2_ again prevents catalyst inhibition by the nitrile product.^[11]^ In this reaction, Coll⋅HCl is not required to achieve catalyst turnover, but it is reasonable to assume that it serves as a proton source as was established earlier for the titanium catalyzed α‐decyanation reaction.^[11]^ Furthermore, it is known to act as a titanium(III) catalyst stabilizing agent.^[19]^ The silyl chloride is proposed to either assist in the liberation of the catalyst by formation of a silylketenimine intermediate or to prevent catalyst inhibition by scavenging the thiol side product through trimethylsilyl thioether formation.[^20^, ^21^] Additional mechanistic investigations are ongoing and the results will be reported in due course.

**Scheme 7:**
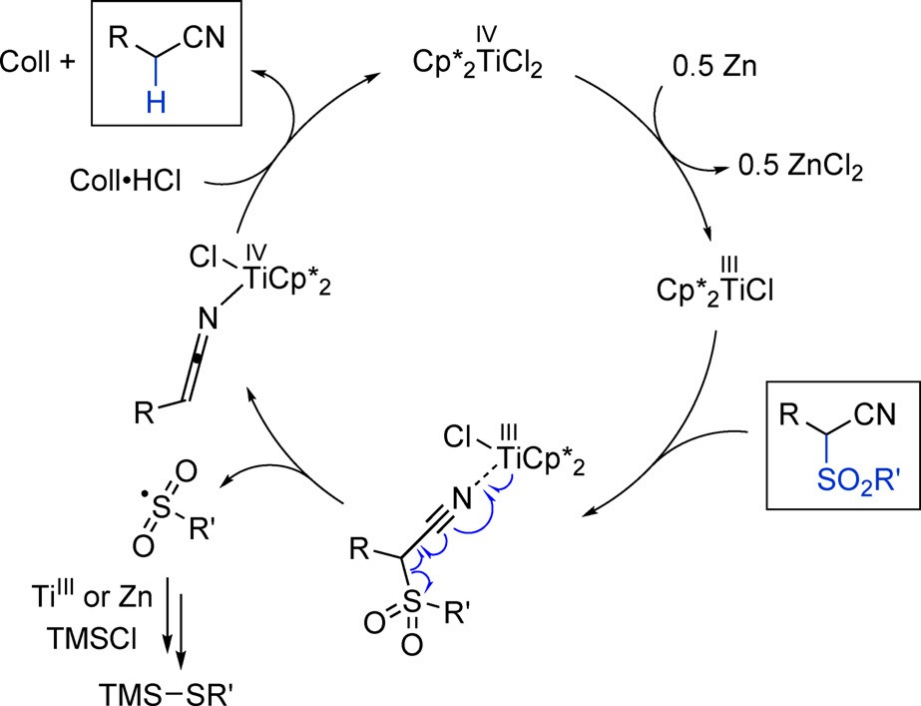
Proposed mechanistic pathway involving the catalyst‐controlled scission of the C−S bond.

In conclusion, a broadly applicable titanium(III)‐catalyzed desulfonylation of α‐arylsulfonyl and α‐methanesulfonyl nitriles has been developed. The reaction can be applied to the α‐desulfonylation of ketones as well. We have further realized a one‐pot desulfonylative cross‐coupling with acrylonitrile as a representative Michael acceptor. First experiments suggest that the reaction proceeds via a homolytic C−S cleavage that is induced by electron transfer from a single titanium(III) species. The reaction complements the existing desulfonylation protocols and the environmentally friendly early base metal catalyst as well as the non‐problematic additives and solvents render it attractive for future applications in organic synthesis.

## Conflict of interest

The authors declare no conflict of interest.

## Supporting information

As a service to our authors and readers, this journal provides supporting information supplied by the authors. Such materials are peer reviewed and may be re‐organized for online delivery, but are not copy‐edited or typeset. Technical support issues arising from supporting information (other than missing files) should be addressed to the authors.

Supplementary
