# Supplementary material for: A Titanium‐Catalyzed Reductive α‐Desulfonylation
Source: Chemistry. 2021 Mar 5;27(20):6178–82. doi: 10.1002/chem.202005400 (PMC8048938; doi:10.1002/chem.202005400)
Supplement: Supplementary file 1 — Supplementary [file CHEM-27-6178-s001.pdf]

Chemistry–A European Journal

Supporting Information

A Titanium-Catalyzed Reductive α-Desulfonylation

Christoph Kern, Jan Selau, and Jan Streuff*[a]

Table of Contents

Materials and Methods **S2**

Additive Synthesis **S3**

Substrate Synthesis **S3**

Representative procedure for the α-sulfonyl nitrile synthesis (**1a**): S3

Titanium-Catalyzed Desulfonylation Reactions **S11**

Extended Screening Results for the Catalytic Reductive Desulfonylation S11

Additive Influence S11

Representative Procedure for the Catalytic Desulfonylation Reaction (**1a** → **2a**) S12

Experimental Details and Characterization Data for Products **2b**–**r** S12

Michael Addition/Desulfonylation Tandem Reaction **S17**

Optimization of the Desulfonylative One-Pot Coupling with Acrylonitrile S17

Final Optimized Procedure S17

Reaction in Absence of Titanium Catalyst S18

Control Experiments (eqs 2 and 3) **S18**

Background Experiment with Thioether **5** S18

Desulfonylation with a Stoichiometric Amount of Cp^*_2TiCl S18

A note on potential byproducts emerging from the phenylsulfonyl radical S19

Computational Details **S20**

Coordinates of Optimized Structures in THF S21

Coordinates of Optimized Structures in Toluene S28

References **S35**

NMR Spectra **S37**

Materials and Methods

Reactions involving air sensitive reagents were performed in flame-dried Schlenk tubes or Schlenk flasks under argon atmosphere (argon 5.0) and using absolute solvents unless noticed otherwise. Absolute THF and absolute toluene were dried over potassium under argon atmosphere and freshly distilled prior to use. Dichloromethane, pentane, ethyl acetate and diethyl ether were purchased in p.a. quality. Cyclohexane for column chromatography was purchased in technical quality and purified by destillation with a rotary evaporator. An IKAmag temperature modulator in combination with an oil bath was used to control the reaction temperatures. Thin-layer chromatography (TLC) was performed using E. Merck silica gel 60 F254 precoated plates (0.25 mm) and visualized by UV fluorescence quenching or staining ($KMnO_4$). In general, Macherey- Nagel Silica gel 60 (particle size 0.04–0.063 mm) was used for flash chromatography. ^{1}H, ^{19}F and ^{13}C NMR spectra were recorded on a Bruker DRX 500 (^{1}H: 500 MHz and ^{13}C: 125 MHz), a Bruker Avance II 400 (^{1}H: 400 MHz and ^{13}C: 100 MHz), and a Bruker Avance III 300 (^{1}H: 300 MHz and ^{19}F: 282 MHz) spectrometer and reported to $CDCl_3$ [$\delta(^{1}H) = 7.26$ ppm and $\delta(^{13}C) = 77.16$ ppm]. The following abbreviations were used: s = singlet, d = doublet, t = triplet, q = quartet, m = multiplet. NMR spectra were recorded at 23 ºC unless noted otherwise. Melting points were determined using a Coesfeld MPM-HV2 melting point apparatus and are uncorrected. IR spectra were recorded on a Thermo Scientific Nicolet iS10 FT-IR spectrometer equipped with a diamond ATR unit and are reported in frequency of absorption. Low- and high-resolution mass analyses were performed by the service department at the Institute for Organic Chemistry and Biochemistry, University of Freiburg using a Thermo Finnigan TSQ 700 for electron impact ionization (EI) at 70 eV, 200 °C. High resolution mass analyses (HRMS) were carried out on a Thermo Exactive with Orbitrap-Analyzer using atmospheric pressure chemical ionization (APCI) or electrospray ionization (ESI).

Half-wave reduction potentials were determined from cyclic voltammograms that were recorded with a VersaSTAT 4 Potentiostat Galvanostat under moisture- and oxygen-free conditions. A standard three-electrode setup was employed, with a Pt millielectrode (model G0228, AMETEK) as working electrode. The counter electrode consisted of a platinum wire in an electrolyte (0.2 M Bu_4NPF_6 in THF), while the reference electrode consisted of a silver wire in a 3 M NaCl/sat. AgCl solution.[1]

Additive Synthesis

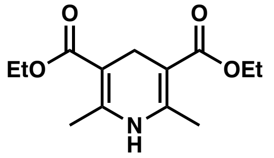

S1

Diethyl 2,6-dimethyl-1,4-dihydropyridine-3,5-dicarboxylate.[2] Synthesized from ethyl 3-oxobutanoate (26.0 g, 200 mmol, 4.0 equiv), formaldehyde (3.72 ml, 49.9 mmol, 1.0 equiv) and ammonium acetate (7.69 g, 99.8 mmol, 2.0 equiv) following literature procedure. The NMR data matched the literature values. ^{1}H NMR (500 MHz, $CDCl_3$): δ = 1.28 (t, *J* = 7.1 Hz, 6H), 2.19 (s, 6H), 3.26 (s, 2H), 4.16 (q, *J* = 7.1 Hz, 4H), 5.16 (s, 1H). ^{13}C NMR (126 MHz, $CDCl_3$): δ = 14.63, 19.34, 24.95, 59.80, 99.72, 144.87, 168.17.

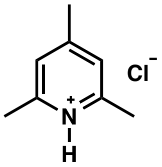

S2

2,4,6-Collidine hydrochloride.[3] Synthesized from 2,4,6-trimethylpyridine (5.29 ml, 40 mmol, 1.0 equiv), acetyl chloride (3.14 ml, 44 mmol, 1.1 equiv) and methanol (2.43 ml, 60 mmol, 1.5 equiv) following the literature procedure. The NMR data matched the literature values. ^{1}H NMR (400 MHz, $CDCl_3$): δ = 2.52 (s, 3H), 2.91 (s, 6H), 7.19 (s, 2H). ^{13}C NMR (101 MHz, $CDCl_3$): δ = 19.37, 22.10, 125.20, 153.25, 157.73.

Substrate Synthesis

The compounds **1a** to **1n** (except **2l**) were synthesized according to a modified literature procedure as follows.[4]

<u>*Representative procedure for the α-sulfonyl nitrile synthesis (**1a**):*</u>

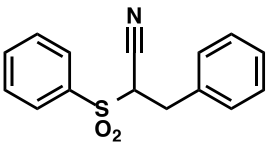

1a

3-Phenyl-2-(phenylsulfonyl)propanenitrile.[5] To a solution of 2-(phenylsulfonyl)acetonitrile (2.43 g, 13.4 mmol, 1.0 equiv) in 60 ml ethanol were added Hantzsch ester **S1** (3.40 g, 13.4 mmol, 1.0 equiv), diethylamine (416.6 μl, 4.03 mmol, 0.3 equiv), and freshly distilled benzaldehyde (1.36 ml, 13.4 mmol, 1.0 equiv). The suspension was stirred at 23 °C for 18 h. Aqueous HCl (1.0 M, 50 ml) and EtOAc (100 ml) were added successively. The layers were separated and the aqueous layer was extracted with ethyl acetate (3 × 40 ml). The combined organic layers were washed with brine and dried over Na_2SO_4, filtered and the solvent was removed under reduced pressure. The product was purified using column chromatography (CH_2Cl_2, R_f = 0.6) and then obtained as a colorless solid in 93% yield (3.40 g, 12.6 mmol). The NMR data matched the literature values. ^{1}H NMR (400 MHz, $CDCl_3$): δ = 3.10 (dd, *J* = 13.5, 11.7 Hz, 1H), 3.60 (dd, *J* = 13.5, 3.8 Hz, 1H), 4.07 (dd, *J* = 11.7, 3.8 Hz, 1H), 7.25–7.37 (m, 5H), 7.64–7.69 (m, 2H), 7.77–7.81 (m, 1H), 8.05–8.08 (m, 2H).^{13}C NMR (101 MHz, $CDCl_3$): δ = 32.91, 59.63, 113.84, 128.33, 129.21, 129.35, 129.84, 129.86, 133.72, 135.53, 135.73.

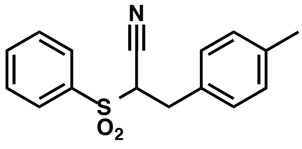

1b

2-(Phenylsulfonyl)-3-(p-tolyl)propanenitrile.[6] Synthesized from 2-(phenylsulfonyl)acetonitrile (136.4 mg, 0.75 mmol, 1.0 equiv) and 4-methylbenzaldehyde following the representative procedure for **1a**. The product was purified using column chromatography (CH_2Cl_2, R_f = 0.5) and then obtained as a colorless solid in 93% yield (200 mg, 0.70 mmol). The NMR data matched the literature values. ^1H NMR (400 MHz, $CDCl_3$): δ = 2.33 (s, 3H), 3.05 (dd, *J* = 13.5, 11.8 Hz, 1H), 3.55 (dd, *J* = 13.5, 3.9 Hz, 1H), 4.05 (dd, *J* = 11.8, 3.9 Hz, 1H), 7.15 (s, 4H), 7.64–7.69p (m, 2H), 7.76–7.81 (m, 1H), 8.04–8.08 (m, 2H). ^{13}C NMR (101 MHz, $CDCl_3$): δ = 21.22, 32.55, 59.74, 113.90, 129.06, 129.82, 129.84, 130.00, 130.59, 135.48, 135.76, 138.12.

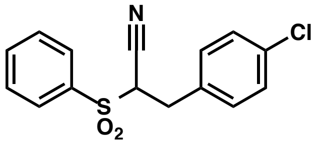

1c

3-(4-Chlorophenyl)-2-(phenylsulfonyl)propanenitrile. Synthesized from 2-(phenylsulfonyl)-acetonitrile (407.7 mg, 2.25 mmol, 1.0 equiv) and 4-chlorobenzaldehyde following the representative procedure for **1a** with 24 h reaction time. The product was purified using column chromatography [pentane/CH_2Cl_2, 1:5→1:10, R_f(pentane/CH_2Cl_2, 1:5) = 0.5] and obtained as a colorless oil in 78% yield (540 mg, 1.77 mmol). ^1H NMR (500 MHz, $CDCl_3$): δ = 3.09 (dd, *J* = 13.7, 11.6 Hz, 1H), 3.55 (dd, *J* = 13.7, 4.0 Hz, 1H), 4.04–4.09 (dd, *J* = 11.6 Hz, 4.0 Hz 1H), 7.19–7.22 (m, 2H), 7.30–7.33 (m, 2H), 7.64–7.68 (m, 2H), 7.77–7.81 (m, 1H), 8.03–8.06 (m, 2H). ^{13}C NMR (126 MHz, $CDCl_3$): δ = 32.17, 59.19, 113.65, 129.49, 129.77, 129.87, 130.60, 132.09, 134.38, 135.49, 135.62. HRMS (pos. ESI): calcd for $C_{15}H_{12}ClNO_2S^+$ $[M–H]^+$: 304.0205, found: 304.0204. MS (EI): m/z (%) = 305.1 $[M]^+$ (5), 166.0 (32), 128.1 (16), 125.0 (15), 101.1 (6), 77.1 (20). IR (ATR): ν [cm^{-1}] = 3067, 2928, 2247, 1583, 1493, 1448, 1330, 1154, 1083, 1016, 911, 854, 723, 586.

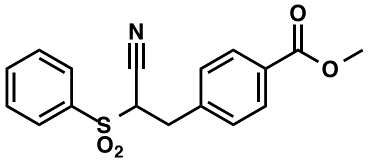

1d

Methyl 4-(2-cyano-2-(phenylsulfonyl)ethyl)benzoate. Synthesized from 2-(phenylsulfonyl)-acetonitrile (362 mg, 2.0 mmol, 1.0 eq) and methyl 4-formylbenzoate following the representative procedure for **1a** with 24 h reaction time. The product was purified using column chromatography [CH_2Cl_2:Et_2O, 100:0→6:1, R_f(CH_2Cl_2) = 0.5] as colorless solid in 81% yield (536 mg, 1.63 mmol). ^1H NMR (400 MHz, $CDCl_3$): δ = 3.17 (dd, *J* = 13.6, 11.6 Hz, 1H), 3.63 (dd, *J* = 13.6, 4.0 Hz, 1H), 3.90 (s, 3H), 4.10 (dd, *J* = 11.6, 4.0 Hz, 1H), 7.35 (d, *J* = 8.3 Hz, 2H), 7.65–7.69 (m, 2H), 7.77–7.81 (m, 1H), 8.00–8.07 (m, 4H). ^{13}C NMR (101 MHz, $CDCl_3$): δ = 32.74, 52.33, 58.98, 113.58, 129.32, 129.84, 129.89, 130.31, 130.56, 135.57, 135.64, 138.74, 166.55. HRMS (pos. ESI): calcd for $C_{17}H_{15}NO_4S^+$ $[M-H]^+$: 328.0649, found 328.0648 MS (CI): *m*/*z* (%) = 347.1 $[M+NH_4]^+$ (100), 330.1 (50), 298.1 (94), 188.2 (33), 156.2 (34) IR (ATR): ν [cm^{-1}] = 2953, 2360, 1717, 1612, 1583, 1447, 1331, 1281, 1181, 1110, 1083, 1020, 746, 720, 585.

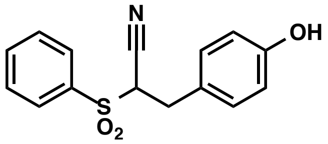

1e

3-(4-Hydroxyphenyl)-2-(phenylsulfonyl)propanenitrile. Synthesized from 2-(phenylsulfonyl)acetonitrile (544 mg, 3.00 mmol, 1.0 equiv) and 4-hydroxybenzaldehyde following the representative procedure for **1a** with 48 h reaction time. The product was purified using column chromatography [pentane/CH_2Cl_2, 1:5→0:100, R_f(pentane/CH_2Cl_2, 1:5) = 0.4] followed by subsequent crystallization from $CHCl_3$ and then obtained as a colorless solid in 64% yield (551 mg, 1.92 mmol). Mp 71 °C. ^{1}H NMR (500 MHz, $CDCl_3$): δ = 3.03 (dd, *J* = 13.7, 11.7 Hz, 1H), 3.51 (dd, *J* = 13.7, 3.9 Hz, 1H), 4.04 (dd, *J* = 11.7, 3.9 Hz, 1H), 5.18 (s, 1H), 6.78 (d, *J* = 8.6 Hz, 2H), 7.12 (d, *J* = 8.6 Hz, 2H), 7.64–7.68 (m, 2H), 7.76–7.80 (m, 1H), 8.04–8.06 (m, 2H). ^{13}C NMR (126 MHz, $CDCl_3$): δ = 32.09, 59.79, 113.94, 116.20, 125.51, 129.78, 129.86, 130.57, 135.56, 135.61, 155.72. HRMS (pos. ESI): calcd for $C_{15}H_{13}NO_3S^+$ $[M–H]^+$: 286.0532, found: 286.0543; MS (EI): m/z (%) = 287.1 $[M]^+$ (11), 145.1 (100), 125.0 (9), 107.1 (21), 77.1 (11). IR (ATR): ν[cm^{-1}] = 3437, 2925, 2251, 1613, 1612, 1597, 1516, 1448, 1329, 1216, 1152, 1103, 909, 840, 720, 591.

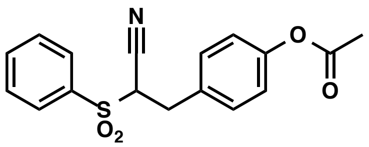

1f

4-(2-Cyano-2-(phenylsulfonyl)ethyl)phenyl acetate. Synthesized from 2-(phenylsulfonyl)acetonitrile (362 mg, 2.0 mmol, 1.0 eq) and 4-formylphenyl acetate following the representative procedure for **1a** with 24 h reaction time. The product was purified using column chromatography (pentane/CH_2Cl_2, 1:10 R_f = 0.4) and obtained as a colorless oil in 39% yield (255 mg, 0.77 mmol). ^{1}H NMR (400 MHz, $CDCl_3$): δ = 2.28 (s, 3H), 3.10 (dd, *J* = 13.7, 11.6 Hz, 1H), 3.57 (dd, *J* = 13.7, 3.8 Hz, 1H), 4.07 (dd, *J* = 11.6, 3.8 Hz, 1H), 7.07 (d, *J* = 8.6 Hz, 2H), 7.28 (d, *J* = 8.6 Hz, 2H), 7.63–7.68 (m, 2H), 7.76–7.80 (m, 1H), 8.03–8.06 (m, 2H). ^{13}C NMR (101 MHz, $CDCl_3$): δ = 21.17, 32.25, 59.36, 113.78, 122.49, 129.78, 129.83, 130.30, 131.19, 135.54, 135.64, 150.68, 169.27. HRMS (pos. ESI): calcd for $C_{17}H_{15}NO_4S^+$ $[M+Na]^+$: 352.0614, found 352.0612. MS (EI): *m*/*z* (%) = 329.1 $[M]^+$ (6), 287.1 (44), 145.1 (100), 125.0 (15), 107.1 (23). IR (ATR): ν[cm^{-1}] = 3056, 2926, 1756, 1508, 1448, 1370, 1332, 1206, 1155, 1083, 1018, 913, 854, 753, 589.

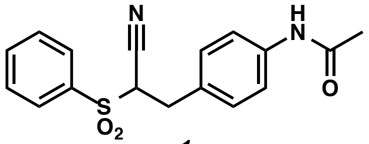

1g

***N*-(4-(2-Cyano-2-(phenylsulfonyl)ethyl)phenyl)acetamide.** Synthesized from 2-(phenylsulfonyl)acetonitrile (399 mg, 2.20 mmol, 1.0 equiv) and *N*-(4-formylphenyl)acetamide following the representative procedure for **1a** with 24 h reaction time. The product was purified using column chromatography (CH_2Cl_2/methanol, 50:1, R_f = 0.2) and obtained as a colorless solid in 83% yield (601 mg, 1.83 mmol). ^{1}H NMR (400 MHz, $CDCl_3$): δ = 2.11 (s, 3H), 3.03 (dd, *J* = 13.7, 11.6 Hz, 1H), 3.50 (dd, *J* = 13.7, 4.0 Hz, 1H), 4.10 (dd, *J* = 11.6, 4.0 Hz, 1H), 7.16 (d, *J* = 8.5 Hz, 2H), 7.46 (d, *J* = 8.5 Hz, 2H), 7.62–7.69 (m, 3H), 7.75–7.79 (m, 1H), 8.02–8.05 (m, 2H). ^{13}C NMR (101 MHz, $CDCl_3$): δ = 24.56, 32.30, 59.38, 113.88, 120.55, 129.16, 129.74, 129.78, 129.84, 135.54, 135.65, 138.12, 168.76. HRMS (pos. APCI): calcd for $C_{17}H_{16}N_2O_3S^+$ $[M+NH_4]^+$: 346.1220, found: 346.1215. MS (CI): m/z (%) = 346.1 $[M+NH_4]^+$ (100), 329.1 (70), 206.2 (5), 186.1 (13), 145.2 (9). IR (ATR): ν [cm^{-1}] = 3371, 3312, 3124, 3065, 2930, 2250, 1690, 1602, 1551, 1448, 1414, 1329, 1262, 1155, 752.

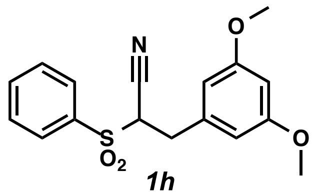

1h

3-(3,5-Dimethoxyphenyl)-2-(phenylsulfonyl)propanenitrile. Synthesized from 2-(phenylsulfonyl)acetonitrile (181 mg, 1.0 mmol, 1.0 equiv) and 3,5-dimethoxybenzaldehyde following the representative procedure for **1a** with 2 h reaction time. The product was purified by crystallization from hot ethanol and then obtained as a yellow solid in 64% yield (212 mg, 0.64 mmol). Mp 102 ºC ^{1}H NMR (400 MHz, $CDCl_3$): δ = 3.00 (dd, *J* = 13.5, 11.8 Hz, 1H), 3.51 (dd, *J* = 13.5, 3.8 Hz, 1H), 3.75 (s, 6H), 4.11 (dd, *J* = 11.8, 3.8 Hz, 1H), 6.37–6.39 (m, 3H), 7.63–7.68 (m, 2H), 7.76–7.80 (m, 1H), 8.04–8.06 (m, 2H). ^{13}C NMR (101 MHz, $CDCl_3$): δ = 33.12, 55.49, 59.38, 100.18, 107.13, 113.88, 129.79, 135.49, 135.62, 135.81, 161.42. HRMS (pos. ESI): calcd for $C_{17}H_{17}NO_4S^+$ [M–H]$^+$: 330.0806, found 330.0806. MS (EI): m/z (%) = 331.2 [M]$^+$ (4), 251.1 (17), 206.0 (36), 181.1 (21), 141.2 (100), 76.7 (59), 51.0 (22). IR (ATR): ν [cm^{-1}] = 2936, 2840, 2246, 1596, 1462, 1448, 1431, 1331, 1205, 1150, 1084, 1066, 922, 836, 797, 754, 725, 685, 637, 607, 583, 569.

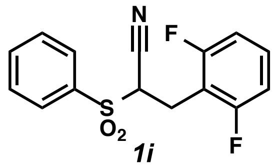

1i

3-(2,6-Difluorophenyl)-2-(phenylsulfonyl)propanenitrile. Synthesized from 2-(phenylsulfonyl)acetonitrile (272 mg, 1.5 mmol, 1.0 eq) and 2,6-difluorobenzaldehyde following the representative procedure for **1a** with 16 h reaction time. The product was purified using column chromatography (pentane/CH_2Cl_2, 1:3→0:100, R_f(CH_2Cl_2) = 0.6] and obtained as a colorless solid in 66% yield (305 mg, 1.0 mmol). ^{1}H NMR (500 MHz, $CDCl_3$): δ = 3.39 (dd, *J* = 13.9, 11.3 Hz, 1H), 3.54 (dd, *J* = 13.9, 4.9 Hz, 1H), 4.25 (dd, *J* = 11.3, 4.9 Hz, 1H), 6.90–6.95 (m, 2H), 7.26–7.32 (m, 1H), 7.66–7.70 (m, 2H), 7.78–7.82 (m, 1H), 8.07–8.09 (m, 2H). ^{13}C NMR (126 MHz, $CDCl_3$): δ = 20.55 (t, *J* = 2.7 Hz), 56.42 , 109.85 (t, *J* = 19.2 Hz), 111.85 (dd, *J* = 19.3, 5.0 Hz), 113.24 , 129.86 , 129.89 , 130.43 (t, *J* = 10.3 Hz), 135.37 , 135.66 , 161.49 (dd, *J* = 249.4, 7.1 Hz). ^{19}F NMR (470 MHz, $CDCl_3$): δ = -113.95. HRMS (pos. ESI): calcd for $C_{15}H_{11}NO_2F_2S^+$ [M–H]$^+$: 306.0395, found: 306.0406. MS (EI): m/z (%) = 307.1 [M]$^+$ (9), 166.0 (97), 146.0 (17), 141.0 (100), 119.0 (9), 77.1 (84), 51.2 (11). IR (ATR): ν [cm^{-1}] = 3069, 2932, 2248, 1627, 1593, 1471, 1448, 1332, 1294, 1269, 1240, 1214, 1155, 1084, 1034, 937, 857, 778, 737, 725, 685, 591.

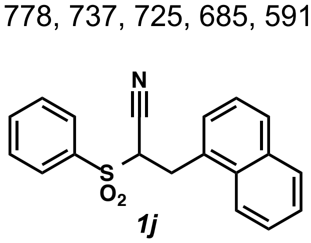

1j

3-(Naphthalen-1-yl)-2-(phenylsulfonyl)propanenitrile. Synthesized from 2-(phenylsulfonyl)acetonitrile (136 mg, 0.75 mmol, 1.0 equiv) and 1-naphthaldehydefollowing the representative procedure for **1a** with 24 h reaction time. The product was purified using column chromatography (pentane/CH_2Cl_2, 1:5, R_f = 0.5) and obtained as a colorless solid in 97% yield (233 mg, 0.73 mmol). ^{1}H NMR (400 MHz, $CDCl_3$): δ = 3.37 (dd, *J* = 14.4, 12.3 Hz, 1H), 4.25–4.30 (m, 2H), 7.41–7.46 (m, 2H), 7.52–7.56 (m, 1H), 7.59–7.63 (m, 1H), 7.66–7.70 (m, 2H), 7.78–7.84 (m, 2H), 7.89–7.94 (m, 2H), 8.12 (d, *J* = 7.9, Hz, 2H). ^{13}C NMR (101 MHz, $CDCl_3$): δ = 30.43, 58.41, 113.83, 122.15, 125.78, 126.33, 127.39, 128.53, 129.31, 129.56, 129.85, 129.86 129.87, 131.04, 134.22, 135.56, 135.68. HRMS (pos. ESI): calcd for $C_{19}H_{15}NO_2S^+$ [M–H]$^+$: 320.0751, found: 320.0749. MS (EI): *m*/*z* (%) = 321.4 [M]$^+$ (19), 178.9 (100), 152.3 (41),

141.0 (27), 76.7 (5). IR (ATR): ν [cm^{-1}] = 3062, 2926, 2247, 1599, 1584, 1512, 1448, 1396, 1332, 1265, 1204, 1154, 1083, 1019, 791, 771, 610, 571.

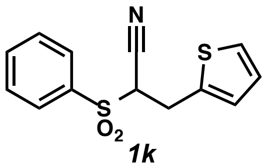

1k

2-(Phenylsulfonyl)-3-(thiophen-2-yl)propanenitrile. Synthesized from 2-(phenylsulfonyl)-acetonitrile (725 mg, 4.0 mmol, 1.0 equiv) and thiophene-2-carbaldehyde following the representative procedure for **1a** with 24h reaction time. The product was purified using column chromatography (pentane/CH_2Cl_2, 1:5, R_f = 0.5) and was obtained as a colorless solid in 82% yield (908 mg, 3.27 mmol). ^{1}H NMR (500 MHz, $CDCl_3$): δ = 3.40 (dd, *J* = 14.7, 11.5 Hz, 1H), 3.78 (dd, *J* = 14.7, 3.9 Hz, 1H), 4.14 (dd, *J* = 11.5, 3.9 Hz, 1H), 6.97 (dd, *J* = 5.1, 3.5, 1H), 7.01–7.02 (m, 1H), 7.24 (dd, *J* = 5.1, 1.1 Hz, 1H), 7.66–7.69 (m, 2H), 7.78-7.81 (m, 1H), 8.05–8.07 (m, 2H). ^{13}C NMR (126 MHz, $CDCl_3$): δ = 27.38, 59.49, 113.73, 126.11, 127.71, 127.95, 129.84, 129.89, 134.87, 135.49, 135.65. HRMS (pos. APCI): calcd for $C_{13}H_{11}NO_2S_2^+$ [M-H]$^+$: 276.0158, found: 276.0157. MS (EI): m/z (%) = 277.0 [M]$^+$ (6), 135.0 (100), 109.0 (11), 97.0 (25), 77.1 (16), 51.0 (5). IR (ATR): ν [cm^{-1}] = 2925, 2247, 1583, 1447, 1330, 1249, 1209, 1186, 1155, 1083, 998, 853, 802, 756, 745, 685, 570.

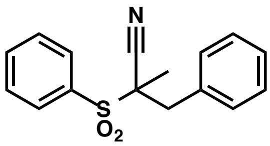

1l

2-Methyl-3-phenyl-2-(phenylsulfonyl)propanenitrile. In a flame dried Schlenk tube 3-phenyl-2-(phenylsulfonyl)propanenitrile (**1a**, 800 mg, 2,95 mmol, 1.0 equiv) was dissolved in 10 ml dimethylformamide. Sodium hydride (60% in mineral oil, 130 mg, 3.24 mmol, 1.1 equiv) was added in three portions over 15 min at 0 °C. Methyl iodide (257 µl, 4.13 mmol, 1.4 equiv) was added and the reaction mixture was stirred at 23 ºC for 3 hours. The product was precipitated by the addition of water (10 ml). The suspension was filtered and the solid was washed with water (3 × 5 ml) and pentane (3 × 5 ml). The colorless solid was dried under high vacuum for several hours and the pure product was obtained in 91% yield (770 mg, 2.70 ml). ^{1}H NMR (400 MHz, $CDCl_3$): δ = 1.52 (s, 3H), 3.14 (d, *J* = 13.3 Hz, 1H), 3.40 (d, *J* = 13.3 Hz, 1H), 7.26–7.38 (m, 5H), 7.65–7.69 (m, 2H), 7.77–7.81 (m, 1H), 8.07–8.11 (m, 2H). ^{13}C NMR (101 MHz, $CDCl_3$): δ = 18.24, 38.30, 63.08, 117.36, 128.37, 129.02, 129.56, 130.61, 131.12, 132.44, 133.97, 135.38. HRMS (pos. APCI): calcd for $C_{16}H_{15}NO_2S^+$ [M+NH_4]$^+$: 303.1162, found: 303.1162. MS (CI): m/z (%) = 303.2 [M+NH_4]$^+$ (100), 163.1 (46), 161.1 (30), 143.1 (31), 94.1 (6). IR (ATR): ν [cm^{-1}] = 3064, 2950, 2230, 1584, 1496, 1447, 1325, 1312, 1153, 723, 686, 606.

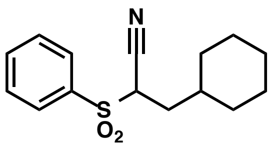

1m

3-Cyclohexyl-2-(phenylsulfonyl)propanenitrile.[7] Synthesized from 2-(phenylsulfonyl)-acetonitrile (362 mg, 2.0 mmol, 1.0 eq) and cyclohexanecarbaldehyde following the representative procedure for **1a** with 18 h reaction time. The product was purified using column chromatography (CH_2Cl_2 R_f = 0,6) and obtained as a colorless solid in 88% yield (490 mg, 1.77 mmol). The NMR data matched the literature values. ^{1}H NMR (400 MHz, $CDCl_3$): δ = 0.86–1.31 (m, 5H), 1.49–1.73 (m, 6H), 1.82 (ddd, *J* = 13.8, 11.7, 4.2 Hz, 1H), 2.00 (ddd, *J* = 13.8, 9.7, 4.2 Hz, 1H), 3.99 (dd, *J* = 11.7, 4.2 Hz, 1H), 7.61–7.66 (m, 2H), 7.74–7.78 (m, 1H), 7.99–

8.02 (m, 2H). ^{13}C NMR (101 MHz, $CDCl_3$): δ = 25.70, 25.93, 26.13, 31.77, 33.51, 33.56, 35.26, 55.83, 114.31, 129.67, 129.74, 135.29, 135.77.

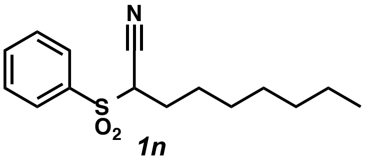

1n

2-(Phenylsulfonyl)nonanenitrile. Synthesized from 2-(phenylsulfonyl)acetonitrile (1.27 g, 7.0 mmol, 1.0 eq) and heptanal following the representative procedure for **1a** with 24 h reaction time. The product was purified using column chromatography (CH_2Cl_2, R_f = 0.6) and was obtained as a colorless solid in 45% yield (875 mg, 3.13 mmol). ^{1}H NMR (400 MHz, $CDCl_3$): δ = 0.87 (t, *J* = 7.2 Hz, 3H), 1.20–1.39 (m, 8H), 1.42–1.53 (m, 1H), 1.57–1.68 (m, 1H), 1.84–1.94 (m, 1H), 2.13–2.22 (m, 1H), 3.91 (dd, *J* = 11.0, 4.4 Hz, 1H), 7.62–7.67 (m, 2H), 7.74–7.78 (m, 1H), 8.00–8.03 (m, 2H). ^{13}C NMR (101 MHz, $CDCl_3$): δ = 14.10, 22.62, 26.75, 26.81, 28.78, 28.85, 31.65, 57.75, 114.14, 129.71, 135.31, 135.87. HRMS (pos. ESI): calcd for $C_{15}H_{21}NO_2S^+$ $[M–H]^+$: 278.1220, found: 278.1223. MS (CI): *m*/*z* (%) = 297.2 $[M+NH_4]^+$ (100), 237.1 (16), 173.2 (7), 141.1 (26), 94.1 (8). IR (ATR): ν [cm^{-1}] =2928, 2858, 1585, 1448, 1333, 1157, 1085, 753, 725, 687, 603.

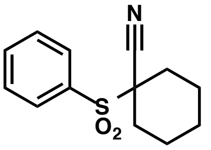

1o

1-(Phenylsulfonyl)cyclohexane-1-carbonitrile.[8] The compound was synthesized according to the literature procedure, from 2-(phenylsulfonyl)acetonitrile (906 mg, 5.0 mmol, 1.0 equiv) and 1,5-dibromopentane (812 μl, 6.0 mmol, 1.2 equiv). The crude product was purified using column chromatography [pentane/CH_2Cl_2, 1:1→0:100, R_f(CH_2Cl_2) = 0.5] and was obtained as a colorless solid in 80% yield (1.00 g, 4.0 mmol). The NMR data matched the literature values. ^{1}H NMR (400 MHz, $CDCl_3$): δ = 1.19–1.30 (m, 1H), 1.50–1.62 (m, 2H), 1.75–1.81 (m, 1H), 1.89–1.98 (m, 4H), 2.09–2.14 (m, 2H), 7.61–7.65 (m, 2H), 7.73–7.77 (m, 1H), 7.99–8.02 (m, 2H).^{13}C NMR (101 MHz, $CDCl_3$): δ = 22.63, 24.41, 29.27, 64.53, 116.99, 129.39, 130.91, 134.00, 135.13.

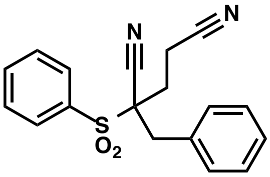

1p

2-Benzyl-2-(phenylsulfonyl)pentanedinitrile. Isolated from a control experiment of the desulfonylative cross-coupling of **1a** and acrylonitrile to **2p** in absence of titanium catalyst (see below).

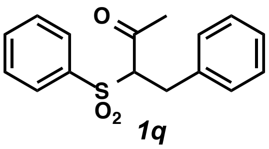

1q

4-Phenyl-3-(phenylsulfonyl)butan-2-one.[9] In a flame dried Schlenk tube K_2CO_3 (270 mg, 1.95 mmol, 1.3 equiv) and 1-(phenylsulfonyl)propan-2-one (297 mg, 1.50 mmol, 1.0 equiv) were suspended in 10 ml acetonitrile. Benzyl chloride (247 mg, 1.95 mmol, 1.3 equiv) was added and the colorless suspension was stirred at 60 °C for 24 hours. The reaction mixture was quenched with water (10 ml) and 10 ml ethyl acetate was added. The layers were

separated and the aqueous layer was extracted with ethyl acetate (3 × 10 ml). The combined organic layers were washed with brine (1x15 ml), dried over sodium sulfate, filtered and the solvent was removed under reduced pressure. The crude product was purified using column chromatography [pentane/Et_2O, 5:1→1:1, R_f(pentane/Et_2O, 5:1) = 0.3]. The pure product was obtained as a colorless solid in 61% yield (265 mg, 0.919 mmol). The NMR data matched the literature values. ^{1}H NMR (400 MHz, $CDCl_3$): δ = 2.18 (s, 3H), 3.13 (dd, *J* = 13.5, 11.6 Hz, 1H), 3.26 (dd, *J* = 13.5, 3.2 Hz, 1H), 4.42 (dd, *J* = 11.6, 3.2 Hz, 1H), 7.03–7.06 (m, 2H), 7.17–7.26 (m, 3H), 7.57–7.62 (m, 2H), 7.69–7.73 (m, 1H), 7.84–7.87 (m, 2H). ^{13}C NMR (101 MHz, $CDCl_3$): δ = 32.70, 33.07, 76.81, 127.35, 128.82, 129.02, 129.32, 129.54, 134.57, 135.86, 136.69, 199.65.

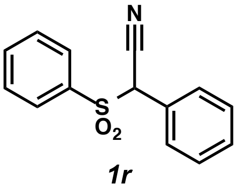

1r

2-Phenyl-2-(phenylsulfonyl)acetonitrile.[10] The compound was synthesized following a modified literature procedure.[11] A flame dried Schlenk tube, equipped with a reflux condenser was charged with tetrakis(triphenylphosphine)palladium(0) (118 mg, 0.102 mmol, 0.075 equiv), sodium hydride (60% in mineral oil) (114 mg, 2.86 mmol, 2.1 equiv) and 2-(phenylsulfonyl)acetonitrile (272 mg, 1.5 mmol, 1.1 equiv). THF (7.5 ml) was added and the orange suspension was stirred for 10 min at 23 °C. Bromobenzene (143 μl, 1.36 mmol, 1.0 equiv) was added and the mixture was stirred under reflux for 1 hour. The reaction mixture was quenched with aqueous HCl (1.0 M, 10 ml) and 10 ml ethyl acetate was added. The layers were separated and the aqueous layer was extracted with ethyl acetate (3 × 10 ml). The combined organic layers were washed with brine (15 ml), dried over sodium sulfate, filtered, and the solvent was removed under reduced pressure. The crude product was purified using column chromatography [pentane/CH_2Cl_2, 1:1→0:100, R_f(CH_2Cl_2) = 0.4] and then crystallized from $CHCl_3$. The pure product was obtained as a colorless solid in 74% yield (258 mg, 1.0 mmol). The NMR data matched the literature values. ^{1}H NMR (400 MHz, $CDCl_3$): δ = 5.14 (s, 1H), 7.26–7.30 (m, 2H), 7.34–7.39 (m, 2H), 7.43–7.47 (m, 1H), 7.49–7.54 (m, 2H), 7.69–7.73 (m, 3H). ^{13}C NMR (101 MHz, $CDCl_3$): δ = 63.22, 113.51, 125.53, 129.16, 129.31, 129.85, 130.19, 130.63, 134.50, 135.35.

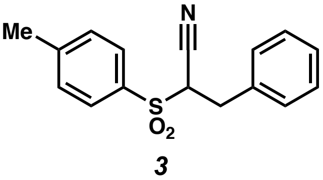

3

3-Phenyl-2-tosylpropanenitrile.[12] Synthesized from 2-tosylacetonitrile (488 mg, 2.5 mmol, 1.0 equiv) and benzaldehyde following the representative procedure for **1a** with 24 h reaction time. The product was purified by column chromatography [pentane/CH_2Cl_2, 1:10→0:100, R_f(pentane/CH_2Cl_2, 1:10) = 0.4] and obtained as colorless solid in 88% yield (627.5 mg, 2.20 mmol). The NMR data matched the literature values. ^{1}H NMR (400 MHz, $CDCl_3$): δ = 2.50 (s, 3H), 3.07 (dd, *J* = 13.6, 11.7 Hz, 1H), 3.57 (dd, *J* = 13.6, 3.8 Hz, 1H), 4.08 (dd, *J* = 11.7, 3.8 Hz, 1H), 7.25-7.37 (m, 5H), 7.45 (d, *J* = 8.1 Hz, 2H), 7.93 (d, *J* = 8.1 Hz, 2H). ^{13}C NMR (101 MHz, $CDCl_3$): δ = 21.90, 32.96, 59.58, 113.95, 128.19, 129.15, 129.25, 129.80, 130.41, 132.65, 133.81, 146.92.

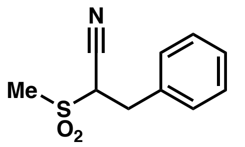

4

2-(Methylsulfonyl)-3-phenylpropanenitrile. Synthesized from 2-(methylsulfonyl)acetonitrile (207 mg, 1.74 mmol, 1.0 eq) and benzaldehyde following the representative procedure for **1a** with 24 h reaction time. The product was purified using column chromatography (CH_2Cl_2, R_f = 0.37) as colorless oil in 87% yield (318 mg, 1.52 mmol). ^{1}H NMR (400 MHz, $CDCl_3$): δ = 3.11 (s, 3H), 3.24 (dd, *J* = 13.7, 10.9 Hz, 1H), 3.54 (dd, *J* = 13.7, 4.0 Hz, 1H), 4.05 (dd, *J* = 10.9, 4.0 Hz, 1H), 7.32–7.42 (m, 5H). ^{13}C NMR (101 MHz, $CDCl_3$): δ = 32.12, 39.71, 57.79, 113.91, 128.55, 129.34, 129.48, 133.44. HRMS (APCI): calcd for $C_{10}H_{11}NO_2S^+$ $[M+H]^+$: 210.0583, found: 210.0583. MS (CI): m/z (%) = 227.0 $[M+NH_4]^+$ (100), 210.1 (5), 147.1 (6), 129.1 (33). IR (ATR): ν [cm^{-1}] = 3030, 2930, 2247, 1498, 1456, 1408, 1322, 1146, 1130, 956, 786, 754, 727, 698.

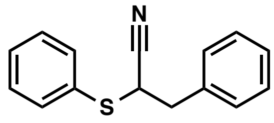

5

3-Phenyl-2-(phenylthio)propanenitrile.[13] In a flame dried schlenk flask sodium hydride (60% in mineral oil) (60 mg, 1.5 mmol, 1.0 equiv) was suspended in acetonitrile (10 ml). 2-(phenylthio)acetonitrile (196 mg, 1.5 mmol, 1.0 equiv) was added and the suspension was stirred at 23 °C for one hour. Benzyl bromide (178 µl, 1.5 mmol, 1.0 equiv) was added and the orange suspension was stirred at 23 °C for 18 hours. The reaction mixture was quenched with water (15 ml) and 20 ml ethyl acetate was added. The layers were separated and the aqueous layer was extracted with ethyl acetate (3 × 20 ml). The combined organic layers were washed with brine (1 × 15 ml), dried over sodium sulfate, filtered and the solvent was removed under reduced pressure. The crude product was purified using column chromatography (pentane/Et_2O, 10:1, R_f = 0,5). The pure product was obtained as a colorless solid in 70% yield (250 mg, 1.04 mmol). The NMR data matched the literature values. ^{1}H NMR (400 MHz, $CDCl_3$): δ = 3.07–3.19 (m, 2H), 3.91 (dd, *J* = 9.0, 6.2 Hz, 1H), 7.25–7.44 (m, 8H), 7.59–7.66zz (m, 2H).^{13}C NMR (101 MHz, $CDCl_3$): δ = 38.98, 39.03, 118.88, 127.89, 128.97, 129.24, 129.63, 129.71, 130.79, 134.71, 135.78.

Titanium-Catalyzed Desulfonylation Reactions

Extended Screening Results for the Catalytic Reductive Desulfonylation

Table S1. Extended screening table.

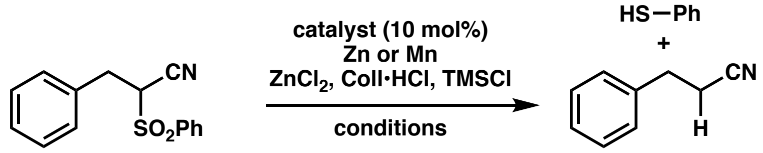

1a (0.25 mmol) ***2a***

entry	catalyst	reductant (equiv)	equiv $ZnCl_2$	equiv Coll•HCl	equiv TMSCl	solvent	*T* / °C	*t* / h	yield / %
1	Cp_2TiCl_2	Zn (3.0)	1.0	2.0	3.0	THF	60	48	56
2	Cp_2TiCl_2	Zn (3.0)	1.0	2.0	3.0	THF	60	48	n.d.[a]
3	***rac*-(ebthi)$TiCl_2$**	Zn (3.0)	1.0	2.0	3.0	THF	60	48	24
4	**$(EtCp)_2TiCl_2$**	Zn (3.0)	1.0	2.0	3.0	THF	60	48	66
5	**$Cp*_2TiCl_2$**	Zn (3.0)	1.0	2.0	3.0	THF	60	48	20
6	Cp_2TiCl_2	**Mn (3.0)**	1.0	2.0	3.0	THF	60	48	32
7	Cp_2TiCl_2	Zn (3.0)	1.0	2.0	3.0	**CH_3CN**	60	48	trace
8	Cp_2TiCl_2	Zn (3.0)	1.0	2.0	3.0	**toluene**	110	48	41
9	**$(EtCp)_2TiCl_2$**	Zn (3.0)	1.0	2.0	3.0	toluene	110	48	60
10	**(ebthi)$TiCl_2$**	Zn (3.0)	1.0	2.0	3.0	toluene	110	48	68
11	**$Cp*_2TiCl_2$**	Zn (3.0)	1.0	2.0	3.0	toluene	110	48	92
**12**	**$Cp*_2TiCl_2$**	**Zn (3.0)**	**1.0**	**2.0**	**3.0**	**toluene**	**110**	**24**	**96**
13	$Cp*_2TiCl_2$ (5 mol%)	Zn (3.0)	1.0	2.0	3.0	toluene	110	24	43
14	$Cp*_2TiCl_2$	Zn (3.0)	1.0	2.0	3.0	toluene	110	**15**	55
15	$Cp*_2TiCl_2$	Zn (3.0)	1.0	2.0	3.0	toluene	**80**	48	71
16	$Cp*_2TiCl_2$	Zn (3.0)	1.0	2.0	3.0	toluene	**60**	48	52
17	**none**	Zn (3.0)	1.0	2.0	3.0	toluene	110	48	trace
18	$Cp*_2TiCl_2$	**Zn (1.0)**	1.0	2.0	3.0	toluene	110	48	33

[a] With a reaction concentration of 1 mol l^{-1}, stirring was no longer possible.

Additive Influence

Reducing the number of additives ($ZnCl_2$, Coll•HCl, TMSCl) led to a diminished reaction outcome (Table S2) and each was previously shown to have a beneficial effects in low-valent titanium catalysis.[1,14,15,16] In our hands, the absence of $ZnCl_2$ led to a significant drop in yield. The removal of Coll•HCl or TMSCl had a smaller although still significant negative effect, resulting in 15–21% lower yields. However, the reaction was still productive and this observation could be of interest for desulfonylations in presence of functional groups that are incompatible with these reagents. For completion, a reaction without TMSCl *and* Coll•HCl and a reaction without all three additives were carried out as well, leading to inferior results. Given the inexpensive and benign nature of the additives, we continued using the optimal conditions from Table 1 in the manuscript.

Table S2. Evaluation of the additive requirement.

change in conditions	yield
none	96%
without $ZnCl_2$	55%
without Coll•HCl	75%
without TMSCl	81%
without TMSCl, Coll•HCl	51%
without TMSCl, Coll•HCl, $ZnCl_2$	34%

The compounds **2a**–**r** were then synthesized according to the following representative desulfonylation procedure on a 0.5 mmol scale (entries in Scheme 2 of the manuscript).

*Representative Procedure for the Catalytic Desulfonylation Reaction (**1a** → **2a**)*

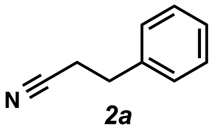

2a

3-Phenylpropanenitrile.[17] A flame-dried, argon-back-filled Young tube was charged with $Cp^*_2TiCl_2$ (19.5 mg, 0.05 mmol, 0.1 equiv), $ZnCl_2$ (68.1 mg, 0.5 mmol, 1.0 equiv), Zn (98.0 mg, 1.5 mmol, 3.0 equiv), and 2,4,6-collidine hydrochloride (**S2,** 158 mg, 1.0 mmol, 2.0 equiv). The tube was evacuated and back-filled with argon three times, before freshly distilled toluene (2 ml) was added. The suspension was stirred for 5 minutes followed by the addition of 3-phenyl-2-(phenylsulfonyl)propanenitrile (**1a**, 136 mg, 0.5 mmol, 1.0 equiv) and TMSCl (190 μl, 1.5 mmol, 3.0 equiv). The reaction vessel was sealed and immersed into a preheated oil bath at 110°C. The reaction mixture was stirred for 24 hours and was then allowed to cool to 23 °C, resulting in a clear solution with a solid precipitate. CH_2Cl_2 (2 ml) was added and the mixture was stirred for 5 minutes under air. The solution was filtered and the residue was rinsed three times with a small amount of CH_2Cl_2 (3 × 2 ml). Here, a spatula was used to suspend the residue in the CH_2Cl_2. The combined filtrates were then concentrated under reduced pressure. The crude product was purified by column chromatography [pentane/Et_2O, 20:1→10:1, R_f(pentane/Et_2O, 3:1) = 0.4] and obtained as colorless liquid in 92% yield (60.4 mg, 0.46 mmol). The NMR data matched the literature values. The reaction carried out on a 0.25 mmol scale as part of the optimization study gave **2a** in 96% yield (31.6 mg, 0.24 mmol). ^1H NMR (400 MHz, $CDCl_3$): δ = 2.62 (t, *J* = 7.4 Hz, 2H), 2.97 (t, *J* = 7.4 Hz, 2H), 7.22–7.37 (m, 5H). ^{13}C NMR (101 MHz, $CDCl_3$): δ = 19.49, 31.76, 119.21, 127.39, 128.39, 129.03, 138.20.

*Experimental Details and Characterization Data for Products **2b–r***

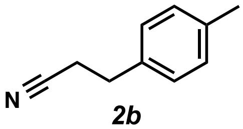

2b

3-(*p*-Tolyl)propanenitrile.[18] Synthesized from 2-(phenylsulfonyl)-3-(p-tolyl)propanenitrile (**1b**, 143 mg, 0.5 mmol, 1.0 equiv) following the representative catalytic desulfonylation procedure. The product was purified by column chromatography (hexanes/EtOAc, 10:1 , R_f = 0.3) and obtained as a colorless liquid in 86% yield (62.5 mg, 0.43 mmol). The NMR data matched the literature values. ^1H NMR (400 MHz, $CDCl_3$): δ = 2.35 (s, 3H), 2.59 (t, *J* = 7.4 Hz, 2H), 2.92 (t, *J* = 7.4 Hz, 2H), 7.12–7.17 (m, 4H). ^{13}C NMR (101 MHz, $CDCl_3$): δ = 19.53, 21.12, 31.27, 119.28, 128.21, 129.61, 135.15, 136.92.

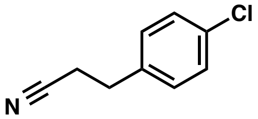

2c

3-(4-Chlorophenyl)propanenitrile.[18] Synthesized from 3-(4-chlorophenyl)-2-(phenylsulfonyl)propanenitrile (**1c**, 153 mg, 0.5 mmol, 1.0 equiv) following the representative catalytic desulfonylation procedure. The product was purified by column chromatography [pentane/Et_2O, 4:1→2:1, R_f(pentane/Et_2O, 2:1) = 0.25] and obtained as colorless liquid in 79% yield (65.5 mg, 0.395 mmol). The NMR data matched the literature values. ^1H NMR (400 MHz, $CDCl_3$): δ = 2.60 (t, *J* = 7.3 Hz, 2H), 2.93 (t, *J* = 7.3 Hz, 2H), 7.17 (d, *J* = 8.4 Hz, 2H), 7.31 (d, *J* = 8.4 Hz, 2H). ^{13}C NMR (101 MHz, $CDCl_3$): δ = 19.38, 31.04, 118.86, 129.15, 129.77, 133.32, 136.55.

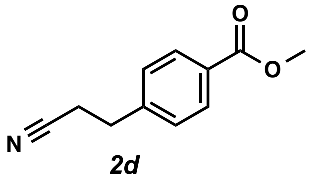

2d

Methyl 4-(2-cyanoethyl)benzoate.[18] Synthesized from methyl 4-(2-cyano-2-(phenylsulfonyl)ethyl)benzoate (**1d**, 165 mg, 0.5 mmol, 1.0 equiv) following the representative catalytic desulfonylation procedure. The product was purified by column chromatography [pentane/CH_2Cl_2, 1:2→1:6, R_f(pentane/CH_2Cl_2, 1:6) = 0.4] and obtained as colorless solid in 83% yield (78.2 mg, 0.413 mmol). The NMR data matched the literature values. ^{1}H NMR (400 MHz, $CDCl_3$): δ = 2.65 (t, *J* = 7.3 Hz, 2H), 3.02 (t, *J* = 7.3 Hz, 2H), 3.91 (s, 3H), 7.31 (d, *J* = 8.2 Hz, 2H), 8.02 (d, *J* = 8.2 Hz, 2H). ^{13}C NMR (101 MHz, $CDCl_3$): δ = 19.09, 31.64, 52.25, 118.76, 128.48, 129.49, 130.35, 143.20, 166.84.

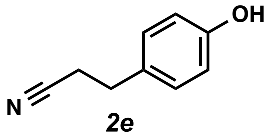

2e

3-(4-Hydroxyphenyl)propanenitrile.[19] Synthesized from 3-(4-hydroxyphenyl)-2-(phenyl-sulfonyl)propanenitrile (**1e**, 144 mg, 0.5 mmol, 1.0 equiv) following the representative catalytic desulfonylation procedure. The product was purified by column chromatography (pentane/Et_2O, 1:2, R_f = 0.4) and obtained as colorless solid in 75% yield (55 mg, 0.37 mmol). The NMR data matched the literature values. ^{1}H NMR (400 MHz, $CDCl_3$): δ = 2.58 (t, *J* = 7.3 Hz, 2H), 2.89 (t, *J* = 7.3 Hz, 2H), 5.13 (s, 1H), 6.79 (d, *J* = 8.7 Hz, 2H), 7.10 (d, *J* = 8.7 Hz, 2H). ^{13}C NMR (101 MHz, $CDCl_3$): δ = 19.85, 30.89, 115.88, 119.35, 129.65, 130.28, 155.04.

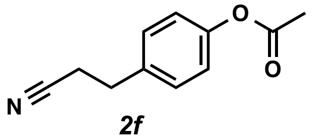

2f

4-(2-Cyanoethyl)phenyl acetate.[1] Synthesized from 4-(2-cyano-2-(phenylsulfonyl)ethyl)phenyl acetate (**2f**, 165 mg, 0.5 mmol, 1.0 equiv) following the representative catalytic desulfonylation procedure. The product was purified by column chromatography [pentane/Et_2O, 10:1→5:1, R_f(pentane/Et_2O, 10:1) = 0.1] and obtained as liquid in 60% yield (57.2 mg, 0.302 mmol). The NMR data matched the literature values. ^{1}H NMR (400 MHz, $CDCl_3$): δ = 2.29 (s, 3H), 2.60 (t, *J* = 7.4 Hz, 2H), 2.95 (t, *J* = 7.4 Hz, 2H), 7.06 (d, *J* = 8.6 Hz, 2H), 7.24 (d, *J* = 8.6 Hz, 2H). ^{13}C NMR (101 MHz, $CDCl_3$): δ = 19.39, 21.18, 31.11, 119.04, 122.10, 129.40, 135.69, 149.97, 169.47.
Further elution (pentane:Et_2O, 1:2) gave **2e** as a second fraction in 13% yield (9.8 mg, 0.066 mmol). The crude nmr of this reaction also showed the presence of *S*-phenylthioacetate, which was not isolated.

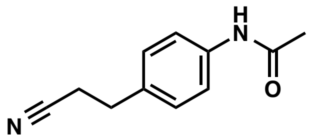

2g

***N*-(4-(2-Cyanoethyl)phenyl)acetamide.**[20] Synthesized from N-(4-(2-cyano-2-(phenylsulfonyl)ethyl)phenyl)acetamide (**1g**, 164 mg, 0.5 mmol, 1.0 equiv) following the representative catalytic desulfonylation procedure. The product was purified by column chromatography [CH_2Cl_2/Et_2O, 2:1→1:1, R_f(CH_2Cl_2/Et_2O, 2:1) = 0.2] and obtained as liquid in 78% yield (73.0 mg, 0.388 mmol). The NMR data matched the literature values. ^{1}H NMR (400 MHz, $CDCl_3$): δ = 2.13 (s, 3H), 2.58 (t, *J* = 7.3 Hz, 2H), 2.89 (t, *J* = 7.3 Hz, 2H), 7.15 (d, *J* = 8.4

Hz, 2H), 7.46 (d, *J* = 8.4 Hz, 2H), 7.78 (s, 1H). ^{13}C NMR (101 MHz, $CDCl_3$): δ = 19.50, 24.49, 31.04, 119.25, 120.49, 128.88, 133.89, 137.33, 168.78.

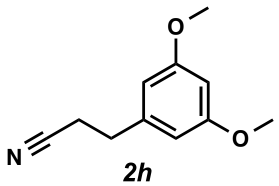

2h

3-(3,5-Dimethoxyphenyl)propanenitrile. Synthesized from 3-(3,5-dimethoxyphenyl)-2-(phenylsulfonyl)propanenitrile (**1h**, 166 mg, 0.5 mmol, 1.0 equiv) following the representative catalytic desulfonylation procedure. The product was purified by column chromatography (hexanes/EtOAc, 10:1, R_f = 0.3) and obtained as liquid in 70% yield (67.0 mg, 0.35 mmol). ^{1}H NMR (400 MHz, $CDCl_3$): δ = 2.61 (t, *J* = 7.4 Hz, 2H), 2.90 (t, *J* = 7.4 Hz, 2H), 3.79 (s, 6H), 6.37 (s, 3H). ^{13}C NMR (101 MHz, $CDCl_3$): δ = 19.32, 32.03, 55.49, 99.20, 106.51, 119.21, 140.47,161.32. HRMS (pos. ESI): calcd for $C_{11}H_{13}NO_2$ $[M+H]^+$: 192.1019, found: 192.1021. MS (EI): m/z (%) = 191.1 $[M]^+$ (67), 151.1 (100), 121.1 (20), 91.1 (3). IR (ATR): ν[cm^{-1}] = 2940, 2840, 2247, 1596, 1462, 1430, 1295, 1266, 1205, 1150, 1067, 835, 733, 701.

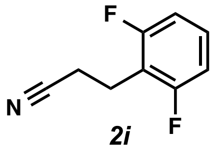

2i

3-(2,6-Difluorophenyl)propanenitrile. Synthesized from 3-(2,6-difluorophenyl)-2-(phenylsulfonyl)propanenitrile (**1i**, 154 mg, 0.5 mmol, 1.0 equiv) following the representative catalytic desulfonylation procedure. The product was purified by column chromatography [pentane/ Et_2O, 30:1→10:1, R_f(pentane/ Et_2O, 10:1) = 0.5] and obtained as colorless liquid in 81% yield (67.9 mg, 0.41 mmol). ^{1}H NMR (500 MHz, $CDCl_3$): δ = 2.64 (t, *J* = 7.5 Hz, 2H), 3.05 (t, *J* = 7.5 Hz, 2H), 6.88–6.93 (m, 2H), 7.21–7.27 (m, 1H). ^{13}C NMR (126 MHz, $CDCl_3$): δ = 17.27, 18.82 (t, *J* = 3.0 Hz), 111.52 (dd, *J* = 20.0, 5.3 Hz), 113.74 (t, *J* = 19.7 Hz), 118.59, 129.25 (t, *J* = 10.3 Hz), 161.45 (dd, *J* = 248.0, 7.9 Hz). ^{19}F NMR (470 MHz, $CDCl_3$): δ = -115.53. HRMS (APCI): calcd for $C_9H_7F_2N^+$ $[M+NH_4]^+$: 185.0885, found: 185.0884. MS (EI): m/z (%) = 167.1 $[M]^+$ (22), 127.1 (100), 107.1 (5), 101.1 (10). IR (ATR): ν [cm^{-1}] = 2929, 2249, 1627, 1990, 1469, 1264, 1237, 1185, 1043, 937, 781, 721.

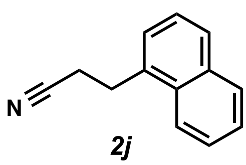

2j

3-(Naphthalen-1-yl)propanenitrile.[21] Synthesized from 3-(naphthalen-1-yl)-2-(phenylsulfonyl)propanenitrile (**1j**, 161 mg, 0.5 mmol, 1.0 equiv) following the representative catalytic desulfonylation procedure. The product was purified by column chromatography (pentane/CH_2Cl_2, 1:5, R_f = 0.2) and obtained as colorless oil in 82% yield (74.3 mg, 0.41 mmol). The NMR data matched the literature values. ^{1}H NMR (400 MHz, $CDCl_3$): δ = 2.76 (t, *J* = 7.6 Hz, 2H), 3.44 (t, *J* = 7.6 Hz, 2H), 7.39–7.47 (m, 2H), 7.51–7.59 (m, 2H), 7.81 (d, *J* = 8.1 Hz, 1H), 7.89–7.94 (m, 2H). ^{13}C NMR (101 MHz, $CDCl_3$): δ = 18.57, 28.93, 119.23, 122.72, 125.70, 126.00, 126.64, 126.66, 128.23, 129.29, 131.23, 134.00, 134.10.

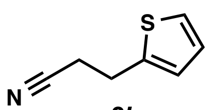

2k

3-(Thiophen-2-yl)propanenitrile.[22] Synthesized from 2-(phenylsulfonyl)-3-(thiophen-2-yl)-propanenitrile (**1k**, 139 mg, 0.5 mmol, 1.0 equiv) following the representative catalytic desulfonylation procedure. The product was purified by column chromatography

[pentane/Et_2O, 20:1→10:1, R_f(pentane/Et_2O, 10:1) = 0.2] and obtained as colorless liquid in 73% yield (50.4 mg, 0.37 mmol). The NMR data matched the literature values. ^{1}H NMR (400 MHz, $CDCl_3$): δ = 2.67 (t, *J* = 7.3 Hz, 2H), 3.18 (t, *J* = 7.3 Hz, 2H), 6.93–6.98 (m, 2H), 7.20 (dd, *J* = 5.1, 1.3 Hz, 1H). ^{13}C NMR (101 MHz, $CDCl_3$): δ = 19.91, 26.01, 118.75, 124.55, 125.76, 127.25, 140.12.

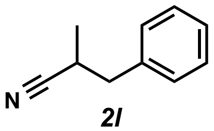

2l

2-Methyl-3-phenylpropanenitrile.[23] Synthesized from 2-methyl-3-phenyl-2-(phenylsulfonyl)propanenitrile (**1l**, 285 mg, 1.0 mmol, 1.0 equiv) following the representative catalytic desulfonylation procedure. The product was purified by column chromatography [pentane/ Et_2O, 20:1→10:1 R_f(pentane/ Et_2O, 10:1) = 0.3] and obtained as colorless oil in 86% yield (125 mg, 0.86 mmol). The NMR data matched the literature values. ^{1}H NMR (400 MHz, $CDCl_3$): δ = 1.34 (d, *J* = 6.9 Hz, 3H), 2.81–2.99 (m, 3H), 7.23-7.38 (m, 5H). ^{13}C NMR (101 MHz, $CDCl_3$): δ = 17.67, 27.57, 40.06, 122.57, 127.30, 128.76, 129.10, 136,95.

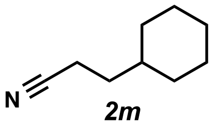

2m

3-Cyclohexylpropanenitrile.[24] Synthesized from 3-cyclohexyl-2-(phenylsulfonyl)propanenitrile (**1m**, 280 mg, 1 mmol, 1.0 equiv) following the representative catalytic desulfonylation procedure. The product was purified by column chromatography [pentane/Et_2O, 30:1→10:1, R_f(pentane/Et_2O, 10:1) = 0.5] and obtained as colorless liquid in 82% yield (113 mg, 0.82 mmol). The NMR data matched the literature values. ^{1}H NMR (400 MHz, $CDCl_3$): δ = 0.86–0.95 (m, 2H), 1.09–1.30 (m, 3H), 1.33–1.44 (m, 1H), 1.55 (td, *J* = 7.4 Hz, 7,2 Hz 2H), 1.64–1.75 (m, 5H), 2.34 (t, *J* = 7.4 Hz, 2H). ^{13}C NMR (101 MHz, $CDCl_3$): δ = 14.80, 26.10, 26.47, 32.68, 32.73, 36.76, 120.17.

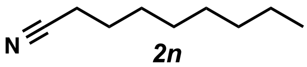

2n

Nonanenitrile.[25] Synthesized from 2-(phenylsulfonyl)nonanenitrile (**1n**, 280 mg, 1 mmol, 1.0 equiv) following the representative catalytic desulfonylation procedure. The product was purified by column chromatography [pentane/Et_2O, 30:1→10:1, R_f(pentane/Et_2O, 10:1) = 0.5] and obtained as colorless liquid in 79% yield (110 mg, 0.79 mmol). The NMR data matched the literature values. ^{1}H NMR (400 MHz, $CDCl_3$): δ = 0.88 (t, *J* = 6.9 Hz, 3H), 1.28–1.32 (m, 8H), 1.40–1.48 (m, 2H), 1.65 (tt, *J* = 7.1 Hz, 7.5 Hz, 2H), 2.32 (t, *J* = 7.1 Hz, 2H). ^{13}C NMR (101 MHz, $CDCl_3$): δ = 14.15, 17.25, 22.71, 25.53, 28.80, 28.85, 29.08, 31.82, 119.92.

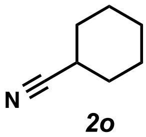

2o

Cyclohexanecarbonitrile.[26] Synthesized from 1-(phenylsulfonyl)cyclohexane-1-carbonitrile (**1o**, 748 mg, 3.0 mmol, 1.0 equiv) following the representative catalytic desulfonylation procedure. The product was purified by distillation and obtained as liquid in 61% yield (200 mg, 1.8 mmol). The NMR data matched the literature values. ^{1}H NMR (400 MHz, $CDCl_3$): δ = 1.36–1.55 (m, 4H), 1.65–1.78 (m, 4H), 1.82–1.88 (m, 2H), 2.58–2.64 (m, 1H). ^{13}C NMR (101 MHz, $CDCl_3$): δ = 24.22, 25.40, 28.16, 29.67, 122.74.

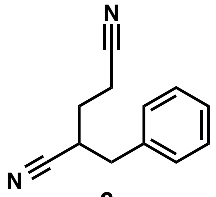

2p

2-Benzylpentanedinitrile.[27] Synthesized from 2-benzyl-2-(phenylsulfonyl)pentanedinitrile (**1p**, 145 mg, 0.448 mmol, 1.0 equiv) following the representative catalytic desulfonylation procedure. The product was purified by column chromatography (pentane/Et_2O, 2:1, R_f = 0.3) and obtained as colorless oil in 66% yield (54.2 mg, 0.294 mmol). The NMR data matched the literature values. ^1H NMR (400 MHz, $CDCl_3$): δ = 1.89–2.03 (m, 2 H), 2.49–2.65 (m, 2H), 2.89–3.03 (m, 3H), 7.23–7.39 (m, 5H). ^{13}C NMR (101 MHz, $CDCl_3$): δ = 15.46, 27.73, 32.82, 38.02, 117.97, 119.96, 127.82, 129.07, 129.10, 135.75.

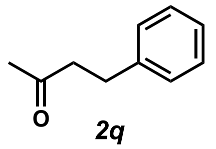

2q

4-Phenylbutan-2-one.[28] Synthesized from 4-phenyl-3-(phenylsulfonyl)butan-2-one (**1q**, 288 mg, 1.0 mmol, 1.0 equiv) following the representative catalytic desulfonylation procedure. The product was purified by column chromatography [pentane/Et_2O, 20:1→5:1, R_f(pentane/Et_2O, 5:1) = 0.4] and obtained as colorless oil in 76% yield (104.5 mg, 0.76 mmol). The NMR data matched the literature values. ^1H NMR (400 MHz, $CDCl_3$): δ = 2.14 (s, 3H), 2.76 (t, *J* = 8.0, 2H), 2.91 (t, *J* = 8.0 Hz, 2H), 7.18–7.22 (m, 3H), 7.27–7.31 (m, 2H). ^{13}C NMR (101 MHz, $CDCl_3$): δ = 29.78, 30.02, 45.14, 126.12, 128.30, 128.50, 141.03, 207.75.

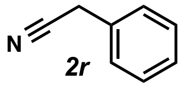

2r

2-Phenylacetonitrile.[1] Synthesized from 2-phenyl-2-(phenylsulfonyl)acetonitrile (**1r**, 129 mg, 0.5 mmol, 1.0 equiv) following the representative catalytic desulfonylation procedure. The product was purified by column chromatography (hexanes/EtOAc, 10:1, R_f = 0.4) and obtained as colorless oil in 89% yield (52 mg, 0.44 mmol). The NMR data matched the literature values. ^1H NMR (400 MHz, $CDCl_3$): δ = 3.75 (s, 2H), 7.31–7.41 (m, 5H). ^{13}C NMR (101 MHz, $CDCl_3$): δ = 23.78, 117.93, 128.07, 128.20, 129.29, 130.08.

Michael Addition/Desulfonylation Tandem Reaction

In initial experiments, acrylonitrile was identified as a suitable partner for the one-pot desulfonylative Michael reaction. Other Michael acceptors such as methyl acrylate, phenyl acrylate, cinnamonitrile, and methyl vinyl ketone were tested as well during our optimization studies, but no coupling products were observed. A broad expansion of this tandem reaction will require an additional thorough optimization that is not part of this study.

Optimization of the Desulfonylative One-Pot Coupling with Acrylonitrile

Table S3. Optimization of the desulfonylative cross-coupling.

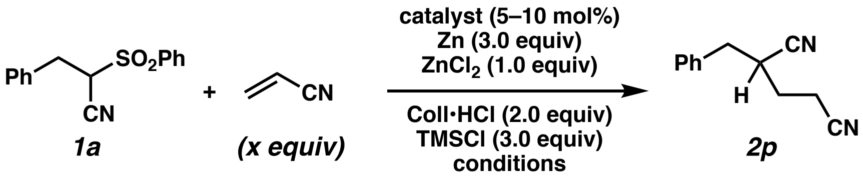

entry	catalyst	solvent	*T* / °C	*t* / h	x	yield / %
1	Cp_2TiCl_2 (10 mol%)	THF	60	24	2	0
2	Cp_2TiCl_2 (10 mol%)	toluene	110	24	2	16
3	$Cp^*_2TiCl_2$ (10 mol%)	toluene	110	24	2	21
4	$Cp^*_2TiCl_2$ (10 mol%)	toluene	110	24	1.6	33
5	$Cp^*_2TiCl_2$ (10 mol%)	toluene	110	48	1.6	37
6	$Cp^*_2TiCl_2$ (10 mol%)	toluene	110	48	1.4	34
7	$Cp^*_2TiCl_2$ (10 mol%)	toluene	90	24	1.6	41
8	$Cp^*_2TiCl_2$ (10 mol%)	toluene	70	24	1.6	37
9	$Cp^*_2TiCl_2$ (10 mol%)	toluene	70	48	1.6	50
10	$Cp^*_2TiCl_2$ (10 mol%)	toluene	70	72	1.6	55
11	$Cp^*_2TiCl_2$ (10 mol%)	toluene	80	72	1.6	42
**12**	**$Cp^*_2TiCl_2$ (5 mol%)**	**toluene**	**80**	**72**	**1.6**	**60**
13	$Cp^*_2TiCl_2$ (5 mol%)	toluene	110	24	1.6	<10%

Final Optimized Procedure

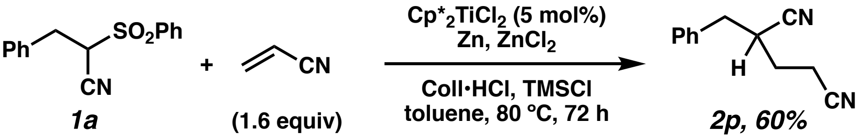

A flame-dried, argon-back-filled Young tube was charged with $Cp^*_2TiCl_2$ (9.73 mg, 0.025 mmol, 0.1 equiv), $ZnCl_2$ (34.1 mg, 0.25 mmol, 1.0 equiv), Zn (49.0 mg, 0.75 mmol, 3.0 equiv) and 2,4,6-collidine hydrochloride (**S2,** 78.8 mg, 0.5 mmol, 2.0 equiv). The tube was evacuated and back-filled with argon three times before freshly distilled toluene (1 ml) was added. The resulting suspension was stirred for 5 minutes followed by the addition of 3-phenyl-2-(phenylsulfonyl)propanenitrile (**1a**, 67.8 mg, 0.25 mmol, 1.0 equiv), acrylonitrile (26.2 μl, 0.40 mmol, 1.6 equiv) and TMSCl (95 μl, 0.75 mmol, 3.0 equiv). The reaction vessel was sealed and immersed into a pre heated oil bath (80 °C). The reaction mixture was stirred for 72 hours and was then allowed to cool to 23 °C, resulting in a clear solution with a solid precipitate. CH_2Cl_2 (2 ml) was added and the mixture was stirred for 5 minutes under air. The solution was filtered and 2 ml acetone was added to partially dissolve the residue. The remaining solid was filtered off and the filtrates were combined followed by concentration under reduced pressure. Product **2p** was purified by column chromatography [pentane/Et_2O, 2:1→1:1, R_f(pentane/Et_2O, 2:1) = 0.3] and obtained as colorless oil in 60% yield (27.3 mg, 0.148 mmol).

Reaction in Absence of Titanium Catalyst

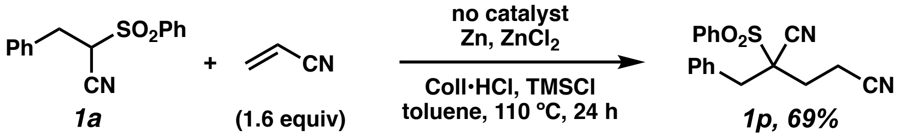

The reaction was carried out following the above procedure without titanium catalyst at 110 °C oil bath temperature and with 24 h reaction time. The crude mixture was purified using column chromatography [pentane/CH_2Cl_2, 1:5→0:100, R_f(pentane/CH_2Cl_2, 1:5) = 0.4] and **1p** was obtained as a colorless solid in 69% yield (55.9 mg, 0.172 mmol). ^{1}H NMR (500 MHz, $CDCl_3$): δ = 2.21 (ddd, J = 14.6, 12.4, 4.4 Hz, 1H), 2.38 (ddd, J = 16.6, 12.0, 4.4 Hz, 1H), 2.55 (ddd, J = 14.6, 12.0, 4.8 Hz, 1H), 2.83 (ddd, J = 16.6, 12.4, 4.8 Hz, 1H), 3.08 (s, 2H), 7.19–7.22 (m, 2H), 7.35–7.38 (m, 3H), 7.70–7.73 (m, 2H), 7.83–7.86 (m, 1H), 8.07–8.10 (m, 2H). ^{13}C NMR (126 MHz, $CDCl_3$): δ = 14.25, 27.17, 39.44, 65.34, 115.38, 117.71, 128.96, 129.44, 129.91, 130.30, 131.00, 131.23, 133.68, 135.98. HRMS (pos. ESI): calcd for $C_{18}H_{16}N_2O_2S^+$ $[M+Na]^+$: 347.0825, found: 347.0821. MS (EI): m/z (%) = 324.1 $[M]^+$ (1), 183.1 (100), 141.0 (66), 115.1 (23), 91.1 (33), 77.1 (37). IR (ATR): ν [cm^{-1}] =3064, 2956, 2200, 1583, 1496, 1447, 1325, 1312, 1135, 1073, 723, 685, 606.

Control Experiments (eqs 2 and 3)

*Background Experiment with Thioether **5***

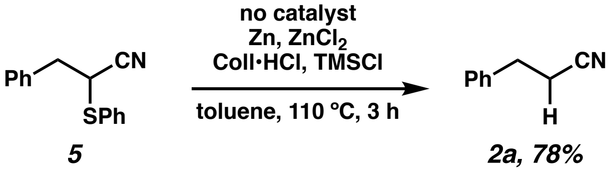

Thioether **5** (0.25 mmol) was submitted to the representative desulfonylation procedure without using any titanium catalyst. After 3 h the reaction was quenched and worked up as described. Purification of the crude material by column chromatography [pentane/Et_2O, 20:1→10:1, R_f(pentane/Et_2O, 20:1) = 0.5] gave **2a** as a colorless liquid in 78% yield (25.7 mg, 0.195 mmol).

Desulfonylation with a Stoichiometric Amount of Cp$_2$TiCl*

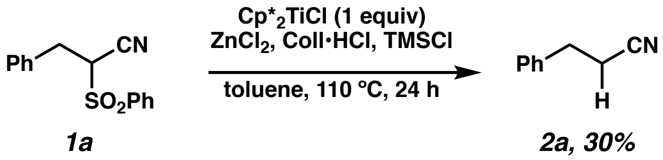

All glassware was treated in a KOH bath, rinsed with distilled water and stored in a drying oven (120 °C) over night prior to the experiment.[29] Oxygen and water must be rigorously excluded during this experiment (argon atmosphere and absolute solvents are required).

In a flame-dried and argon-back-filled Schlenk tube equipped with a magnetic stir bar $Cp^*_2TiCl_2$ (97.3 mg, 0.25 mmol, 1.0 equiv), Zn (572.0 mg, 8.75 mmol, 35.0 equiv) and 2,4,6-collidine hydrochloride (78.8 mg, 0.5 mmol, 2 equiv) were suspended in freshly distilled toluene (2 ml).[30] The mixture was vigorously stirred for 10 minutes and the mixture changed from a brown-red suspension to a dark blue solution, indicating the formation of Cp^*_2TiCl.[31,32] The dark blue solution was filtered using a Schlenk frit into a second flame-dried and argon-back-filled schlenk tube containing a magnetic stir bar. The former reaction flask and Schlenk frit were rinsed with toluene (1 ml) into the Schlenk tube containing the Cp^*_2TiCl solution.

The dark blue Cp^*_2TiCl solution was stirred and 3-phenyl-2-(phenylsulfonyl)propanenitrile (**1a**, 67.8 mg, 0.25 mmol, 1.0 equiv), zinc chloride (34.1 mg, 0.25 mmol, 1.0 equiv) and TMSCl (95 µl, 0.75 mmol, 3.0 equiv) were added. The tube was sealed with a greased glass stopper and immersed into a preheated oil bath at 110°C. The reaction mixture was stirred for 24 hours

and was then allowed to cool to 23 °C, resulting in a clear brown solution with a solid precipitate. CH_2Cl_2 (2 ml) was added and the mixture was stirred for 5 minutes under air. The solution was filtered and the residue was washed three times with a small amount of CH_2Cl_2 (3 × 2 ml) followed by filtration. Here, a spatula was used to suspend the residue in the CH_2Cl_2. The combined filtrates were then concentrated under reduced pressure. ^{1}H NMR analysis of the crude product mixture indicated 30–34% conversion with the remaining material being unreacted **1a**. Product **2a** was purified by column chromatography [pentane/Et_2O, 20:1→10:1, R_f(pentane/Et_2O, 20:1) = 0.5] and obtained as colorless liquid in 30% yield (9.8 mg, 0.075 mmol).

A note on potential byproducts emerging from the phenylsulfonyl radical

After workup of the stoichiometric experiment as described we did not observe byproducts originating from the phenylsulfonyl radical in the crude mixture. However, the literature suggests that phenylsulfonyl radicals undergo a number of reactions including loss of SO_2 to give a phenyl radical,[33] and homo coupling and disproportionation reactions that can be promoted by protic additives such as water (Scheme S1).[34] Reduction by titanium(III) is another possible pathway. This renders the identification and quantification of the byproducts difficult and further investigations will be required to elucidate the nature of the byproducts.[35]

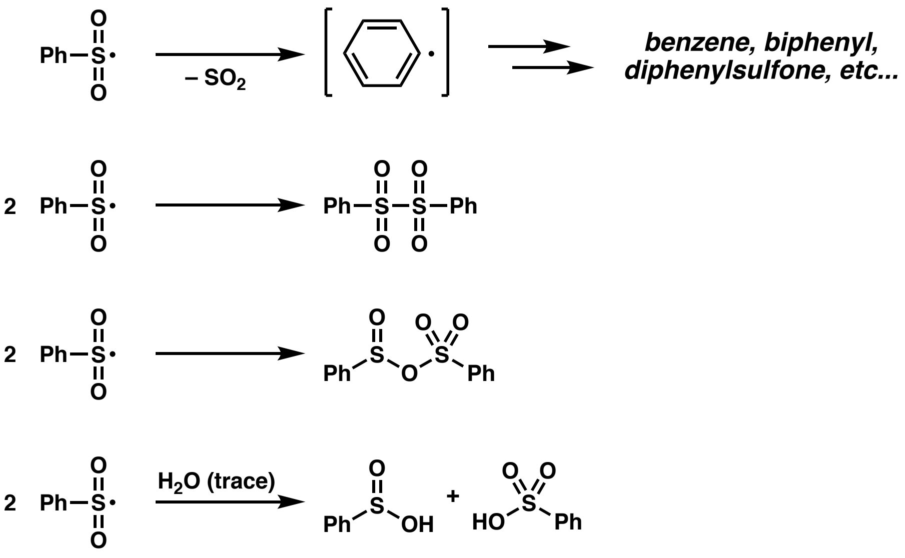

Scheme S1. Potential sequential reactions of phenylsulfonyl radicals.

Computational Details

The Orca 4.2.0 program package was used for the DFT calculations.[36] The RI-J approximation for Coulomb integrals and the COSX numerical integration for HF exchange (RIJCOSX) were applied.[37,38] Furthermore, the D3 dispersion correction with Becke-Johnson damping, D3(BJ), was applied in all calculations.[39,40] All structure optimizations were finalized using the TPSS functional[41] together with the def2-TZVP basis set[42] and matching auxiliary basis sets.[43] The conductor-like polarizable continuum model (CPCM)[44] was applied for the optimizations and the structures were separately optimized in THF and toluene. The optimizations were carried out with the *Grid3 FinalGrid5 TightSCF* options. Frequency analyses were carried out numerically (*NumFreq*). Stationary points (minimum structures) were characterized by the absence of imaginary frequencies. The correction to the Gibbs Free Energy was obtained from the Orca output of the frequency calculation. Single-point calculations were carried out using the PW6B95[45] functional and the def2-QZVP basis set and matching auxiliary basis sets.[46] The single point calculations were carried out with the *Grid4 FinalGrid5* options. The CPCM model together with the correction $\Delta G^{*\rightarrow o}_{solv}$ (= 1.90 kcal mol^{-1}, see Born-Haber cycle in Scheme S2) were applied to obtain the energy in solution.

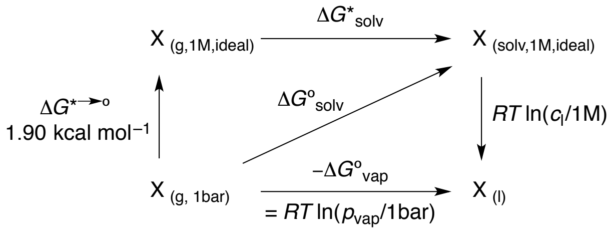

Scheme S2. Interconversion Scheme for the calculation of solvation energies for a compound X.

Table S4. Energies of the calculated species in THF.

**Compound**	**TPSS-CPCM/def2-TZVP / Hartree**	**PW6B95-CPCM/def2-QZVP/ Hartree**	**Correction to *G* / kcal mol^{-1}**	***G*$_{solv}$ / kcal mol^{-1}**
Substrate **1a**	–1183.316044	–1184.510672	124.96	–743164.81
Cp_2TiCl	–1697.215172	–1698.518023	82.32	–1065751.93
Cp^*_2TiCl	–2090.627172	–2092.372941	242.42	–1312739.52
**6-Cp**	–2880.563263	–2883.052967	222.99	–1808918.16
**6-Cp***	–3273.97503	–3276.906601	385.16	–2055902.88
**7-Cp**	–2880.54976	–2883.046509	222.24	–1808914.86
**7-Cp***	–3273.946492	–3276.886136	383.97	–2055891.23

Table S5. Energies of the calculated species in Toluene.

**Compound**	**TPSS-CPCM/def2-TZVP / Hartree**	**PW6B95-CPCM/def2-QZVP/ Hartree**	**Correction to *G* / kcal mol^{-1}**	***G*$_{solv}$ / kcal mol^{-1}**
Substrate **1a**	–1183.3094	–1184.5037	124.94	–743160.47
Cp_2TiCl	–1697.2113	–1698.5139	81.6	–1065750.04
Cp^*_2TiCl	–2090.6241	–2092.3695	242.44	–1312737.35
**6-Cp**	–2880.5534	–2883.042	222.73	–1808911.56
**6-Cp***	–3273.9675	–3276.8984	385.14	–2055897.75
**7-Cp**	–2880.5413	–2883.0378	222.23	–1808909.39
**7-Cp***	–3273.9397	–3276.8793	383.95	–2055886.93

Coordinates of Optimized Structures in THF

1a

C	-4.22005353180608	3.90137879963430	-1.43985336933276
C	-4.86114636988096	5.12135731248710	-1.21847125329247
C	-4.76084260529134	2.71405572969642	-0.93383556072126
C	-6.05034719294752	5.16502627811661	-0.48852331594483
C	-6.59717440853623	3.98340732759449	0.01791004400509
C	-5.95440862201927	2.76553347108165	-0.20290531402577
H	-3.29589267075271	3.86956152364945	-2.01141072787920
H	-6.55088088963701	6.11388695623522	-0.31792186814262
H	-4.43258298302359	6.03617623812619	-1.61784628820380
H	-6.38351993040687	1.84622097292049	0.18930909862933
H	-7.52579490145492	4.00973180021401	0.58107079135114
C	-4.05358802389313	1.39452535368997	-1.14165759434637
C	-3.14910850763236	1.05483292387037	0.06887995730155
H	-3.44209214249983	1.42275635183099	-2.04713349212837
H	-4.77743316688894	0.58069991127808	-1.23370119955818
S	-2.51730458170795	-0.69008079396074	-0.00173364806516
C	-2.02473995934956	1.96368466075389	0.21492379326251
H	-3.73118023747172	1.04418218690964	0.99864391816746
N	-1.11973076269933	2.68473484659417	0.29507942404940
O	-1.66370687952868	-0.87529071111045	1.16368462700695
O	-3.70097323178534	-1.52047757890005	-0.18226842567356
C	-1.52836556761757	-0.75527464027762	-1.46826915319983
C	-2.12864905010638	-1.12239263672436	-2.67615819883404
C	-0.17234769769693	-0.42630443217943	-1.38322206028243
C	0.59691528361177	-0.46620336999645	-2.54416958024196
C	-1.34429201306181	-1.15795978379670	-3.82713198533969
H	-3.18005668191714	-1.38642357878435	-2.70534395644581
H	0.26650967880558	-0.15937331830241	-0.42853199784316
C	0.01186736657922	-0.82836270684156	-3.76014468040103
H	-1.79089175410761	-1.44728099449742	-4.77313683969187
H	0.61803666022683	-0.85945684092466	-4.66047724931713
H	1.65267537449750	-0.21927125838688	-2.49705389486208

Cp_2TiCl

C	-2.31105101494996	0.41549106298663	0.85539033691298
C	-1.84504028824797	1.18553425526410	-0.24616440215079
C	-1.44294143951316	0.27539692163714	-1.26092716609844
C	-1.62991955854157	-1.04913028886479	-0.77539851770280
H	-1.82136206615109	2.26540613709399	-0.30946761558333
C	-2.17627281702495	-0.95789456794514	0.52923055253946
H	-2.65541363064501	0.80836524390958	1.80160520454232
H	-2.39958910225287	-1.78988179430532	1.18444294490347
Ti	-0.00001071965765	-0.00000602976057	0.57486944768783
C	1.62990296289875	1.04913319628919	-0.77538132199140
C	2.17625484114881	0.95789795589938	0.52924748216062
C	1.44294474615846	-0.27539602413739	-1.26091759828547
C	1.84502958672839	-1.18553266807640	-0.24615631670758
C	2.31102662084186	-0.41549890890422	0.85540697108573
H	1.04063996256396	-0.54678585346897	-2.22640918847471
H	1.38962738461847	1.96184108930746	-1.30465598801865
H	2.39960515540852	1.78993533469379	1.18442185204079
H	1.82137841886483	-2.26539623261035	-0.30947436313259
H	2.65550114740287	-0.80839613714552	1.80159794114837
Cl	-0.00002086193590	-0.00000101333307	2.92983749322552

H	-1.04062033608471	0.54673883630773	-2.22640728656608
H	-1.38966899163009	-1.96182051483725	-1.30469056153523

Cp*$_2$TiCl

C	-0.68038967241079	1.87327587264581	1.42674296709422
C	-1.89340961374101	2.18541082015473	0.74482127638325
C	-2.78223925565527	1.08206327299668	0.89373012183802
C	-2.11645190540836	0.08982622108834	1.68029897119531
C	-0.79816557960753	0.55862666132373	1.96809784308151
Ti	-0.91464313601083	0.32509008804844	-0.37754200927802
C	-0.70541825308987	-0.67699738062877	-2.53986517878485
C	0.18390131966067	-1.36026618332252	-1.66001407695055
C	-2.03801645826523	-0.87790285956406	-2.07307583104199
C	-1.97216399042391	-1.74512116590041	-0.93922974649491
C	-0.59455989814235	-2.01117532870115	-0.65914355498263
Cl	0.62543218239192	1.84501723266168	-1.35161295744542
C	-0.33476090029705	0.04597104118052	-3.79850339127151
H	-0.96109467791684	0.92923549047490	-3.95501781792993
H	-0.47379806231749	-0.61823595648183	-4.66277127167732
H	0.70765826869660	0.36986485567106	-3.78094526985447
C	-3.28809959961457	-0.43944958366194	-2.78062074647492
H	-3.17318297009774	0.55721840512501	-3.21843663876748
H	-4.14392519692923	-0.41468734831781	-2.10186932731110
H	-3.53539389352240	-1.13014713962641	-3.59908069525485
C	-3.14402887198754	-2.50368333176467	-0.39141209804691
H	-4.02425831855086	-1.87470626080470	-0.23776611348587
H	-2.90810134382373	-3.00487872764726	0.54760551537753
H	-3.42751009914048	-3.28025807307651	-1.11517341008961
C	-0.05964158899643	-2.95023484427339	0.38294894728969
H	0.07570667179448	-3.95713231244759	-0.03526750221955
H	-0.73801593545081	-3.03894425833686	1.23487300275390
H	0.91307205479927	-2.61917770395198	0.75917432505994
C	1.67773789098164	-1.41453428557775	-1.78008106789704
H	2.06567903879601	-0.51426603790417	-2.26315192370741
H	1.98632282047659	-2.28307415934108	-2.37755249489323
H	2.15303666906349	-1.50550061441346	-0.79822005840276
C	0.20412312078701	-0.11775383965669	2.85890099692012
H	0.08422706050696	-1.20419051547980	2.84232840534584
H	0.08657273117422	0.21103804080958	3.90123972550599
H	1.22961765360053	0.11496395057537	2.55598406113547
C	-2.76370234955850	-1.06865273731746	2.37872643163599
H	-3.72558602489066	-1.33100073244235	1.93748176606290
H	-2.94766036757806	-0.78914962006870	3.42522258221591
H	-2.13339147635820	-1.96133591083699	2.39332036456363
C	-4.21736945698322	1.06218725727658	0.45473058184939
H	-4.35146336030114	1.57212592575591	-0.50374168034865
H	-4.85133136741935	1.57545591565297	1.19059936651011
H	-4.59678153003378	0.04311864069343	0.35162332585692
C	-2.19334530576010	3.46510565050569	0.02202050430303
H	-2.90215904431467	3.30326251695251	-0.79629177576476
H	-1.28295894713040	3.90056126231937	-0.39845371774831
H	-2.63920606399868	4.20195911076008	0.70390231950788
C	0.47738552842377	2.79703170096782	1.65095422857496
H	0.51873623777170	3.57913379379663	0.89045671536013
H	1.43076184961393	2.26077991928042	1.63606615475260
H	0.37992341718846	3.27858326482907	2.63381985594980

6-Cp

C	-0.91577762309196	0.23778569212775	2.32178256371044
C	-2.04758596330829	0.63803181754122	1.56240201691784
C	-2.40378886392045	-0.43219226136137	0.71274745533427
C	-1.50495581288072	-1.51190997738304	0.96198473253350
H	-2.51267041924598	1.61274807645787	1.58581105383908
C	-0.59190500871376	-1.10309833164989	1.95726099128013
H	-0.37802166913321	0.85236089513813	3.03015322576021
H	0.22807732978031	-1.69223437238796	2.34474264853886
Ti	-0.21600591100277	0.28952993006531	0.07684743479525
C	0.44974716018598	0.98449436017816	-2.13854598784310
C	1.29315220701308	-0.08570604295527	-1.77103567726623
C	-0.88711672190127	0.50703099148941	-2.17807108139240
C	-0.85628825339765	-0.87856563304131	-1.85686668746997
C	0.48816611853342	-1.24229191043801	-1.57752244455511
H	-1.76685527042430	1.09091969887984	-2.41324096133624
H	0.75950891547043	2.00553778330024	-2.29781570214485
H	2.36175787516361	-0.02230369956554	-1.62268355932996
H	-1.71180458996697	-1.53726521250733	-1.81800643103928
H	0.83522537934276	-2.22239440860690	-1.28161925120389
H	-3.20593736502581	-0.42814809801260	-0.01316736676188
H	-1.50572586411406	-2.46890404894616	0.45867670099486
Cl	-0.06677930837711	2.74687986387302	0.34121032806135
C	3.04811697045504	-2.95751189052414	0.77926064131283
C	2.15198490912125	-4.00355063055818	1.00211638328310
C	3.79655314303961	-2.42467060335313	1.83412232067118
C	1.99446722967815	-4.52667645254492	2.28631569776752
C	2.74318699583790	-4.00463999705592	3.34427194701541
C	3.63859068819489	-2.95933072345841	3.11913359759982
H	3.16747051657610	-2.54675600109304	-0.21998771571705
H	1.29435463074550	-5.33813370660661	2.46266262198268
H	1.57592102849143	-4.40797035226582	0.17455633137566
H	4.21596296194685	-2.55285039368088	3.94636084817007
H	2.62910572422106	-4.41160868608978	4.34498417762512
C	4.69797346997473	-1.23330330808833	1.60206642370853
C	3.99701128688770	0.05029509592206	2.12044512676412
H	4.91251506429052	-1.12201012274658	0.53569031228159
H	5.64323373082755	-1.32455685599191	2.14168924260708
S	4.99549506156691	1.59531130323303	1.81718381167721
C	2.69007255113406	0.22038019530050	1.53332344444832
H	3.90959073557665	0.03374021159603	3.21319385050522
N	1.66673463802183	0.27770395958537	0.98768598382049
O	4.34249791239261	2.65636782000784	2.57197721008227
O	6.38250074648743	1.24343666567582	2.09401732958054
C	4.79156872056624	1.90640298186802	0.08608529188141
C	5.68961500604789	1.33392058710602	-0.81989432399031
C	3.71835289097783	2.69937773023737	-0.33124312345302
C	3.55171348761173	2.92721526778121	-1.69498743031405
C	5.50258528990315	1.56709914733053	-2.18094840996433
H	6.52074332335503	0.73556408592301	-0.46236738662499
H	3.02821739243277	3.12080578662669	0.39067801368255
C	4.43751895162167	2.36135859483989	-2.61474671845465
H	6.19092603099861	1.13452112251163	-2.90002563748291
H	4.29833908184834	2.54094998657399	-3.67656414958499
H	2.72667348818328	3.54417406974322	-2.03512971367937

6-Cp*

C	-0.89245873130346	0.90333586169782	2.26110177062422
C	-1.99951485748622	1.33289930993961	1.46727734172543
C	-2.63674583157132	0.18173254916826	0.93562151802946
C	-1.93740614088754	-0.97314408298536	1.41483324615681
C	-0.86418727582605	-0.52760831801779	2.23001776338565
Ti	-0.36965119356442	0.27741262434818	0.02024116082493
C	0.22638315073651	0.50635374141552	-2.37020578600008
C	1.07631417202133	-0.46842839495694	-1.78885577204568
C	-1.11554354108932	0.05168258630167	-2.26071908383250
C	-1.08660112751049	-1.25693205315539	-1.67557357918991
C	0.26439225105510	-1.56048592175591	-1.33815596342749
Cl	0.25058846207736	2.65381109255066	-0.21461105758024
C	4.46373650255512	-3.32632197990987	-0.04132081513688
C	3.98742477192879	-4.62238196646481	-0.23925388738741
C	4.19508351888285	-2.63865173601775	1.14812894242414
C	3.22554313002024	-5.24621849203993	0.74949234083064
C	2.95068102295301	-4.56822783292975	1.93826488886356
C	3.43527708514730	-3.27529522573287	2.13726334221681
H	5.04724846862164	-2.83883437909194	-0.81855226062892
H	2.84338812990938	-6.25080694393899	0.59286317833485
H	4.20148656734536	-5.13870517227272	-1.17071367093103
H	3.21394885686842	-2.76679111007794	3.07157367546354
H	2.35509327414500	-5.04325352622897	2.71266942275381
C	4.75240392357086	-1.24318133414185	1.33885915935535
C	3.78141938693551	-0.29737748976056	2.07224572949203
H	5.00320069844252	-0.81549451394194	0.36389347263634
H	5.67235147863080	-1.27444872240971	1.93219441041387
S	4.58228423866871	1.37342171349814	2.36571723887976
C	2.51169432212991	-0.12555283344500	1.41316213679119
H	3.61544127961253	-0.62217372384611	3.10681574908252
N	1.48573078052910	0.03866416692120	0.88148117105814
O	3.60963128427684	2.16784150505972	3.10502559164324
O	5.89069241378623	1.09616296167536	2.95146420486923
C	4.78931935754756	2.05681351655830	0.74880232276975
C	5.99510853069888	1.85555766674222	0.06899839879862
C	3.71933786764581	2.74913250478322	0.17564868591417
C	3.87191244635976	3.25973994610510	-1.11205673437211
C	6.12921948902877	2.37379147585795	-1.21707164993139
H	6.80709979981650	1.31708823145648	0.54564927207916
H	2.78361315826627	2.87355023391470	0.70883759263533
C	5.07037692154414	3.07388290351029	-1.80397126586164
H	7.05945100680897	2.23413326133141	-1.75874509869018
H	5.18176171683040	3.47519913692426	-2.80694219016255
H	3.04685161095522	3.79580092101266	-1.56944260729997
C	0.01849308779797	-1.42853675968290	3.04206317314339
H	0.42695406425365	-2.24889734222940	2.44361370772238
H	-0.55160558367699	-1.87384567320206	3.86873251775129
H	0.85383034161710	-0.87737132403334	3.48061721308227
C	-0.01108017910418	1.79327189242191	3.08248452263154
H	0.19042719331577	2.72987031083764	2.55531522483200
H	0.95046242371567	1.32211604208226	3.30015267828409
H	-0.49046297956486	2.03305985072192	4.04167441837024
C	-2.51489521106508	2.73442335398540	1.34649305062316
H	-2.88755014066035	2.94247670771464	0.33901709315566
H	-1.73274153489571	3.46141871090390	1.57078176500864
H	-3.34559648519803	2.88973691912654	2.04986673940512

C	-3.96390299437119	0.20877903401837	0.23736818223320
H	-4.16833917226407	-0.71486230921653	-0.30655275236436
H	-4.03576696549948	1.04477874846539	-0.46330666644075
H	-4.76715182248659	0.33570097145558	0.97663306144296
C	-2.41620281403720	-2.39505727989805	1.38440699638125
H	-3.24168166836891	-2.53542104642712	0.68611905752842
H	-2.77814104270235	-2.67146533539899	2.38444971610804
H	-1.62192501241426	-3.10232433713118	1.12743605323686
C	-2.19812760849366	-2.25804966223135	-1.77501906410158
H	-2.25123036078754	-2.61371073577728	-2.81355315352792
H	-3.17641614368768	-1.84084017638765	-1.53084328443799
H	-2.02312010125600	-3.12863045586890	-1.14252506460642
C	0.78320106695004	-2.85143820924118	-0.77774872873453
H	1.18497500994870	-3.49451055134388	-1.57212221139057
H	-0.00495889134297	-3.41162291325081	-0.26849332341659
H	1.59131980341202	-2.68326628896641	-0.05985206563508
C	2.57434971840978	-0.43117744122455	-1.80231899465806
H	2.95952552366772	0.54801297789876	-1.50310250598901
H	2.95017859313806	-0.64311563794051	-2.81220147326494
H	2.99037687706069	-1.18792588494619	-1.13344278933980
C	0.67863258784049	1.71762549655353	-3.12148868758755
H	-0.05949397278342	2.52084353509308	-3.07082694565512
H	0.82805215217687	1.45514605099301	-4.17911928444820
H	1.62250022773347	2.10063076881546	-2.72841226100506
C	-2.30893633917692	0.73911998505359	-2.85789782542342
H	-2.33120233808577	1.80190583022052	-2.59364742214482
H	-3.24214775777037	0.28459907232296	-2.51894453563679
H	-2.28732392845858	0.67413094806327	-3.95449843670651

7-Cp

C	-1.08861325515481	-2.37337904610779	1.33593638476675
C	-1.78771030686536	-2.93179296792776	0.23008978802143
C	-1.89923871538859	-4.32201597559158	0.44361800890543
C	-1.28464063880118	-4.62930759716290	1.69358670185695
H	-2.12284297712203	-2.39390814907313	-0.64517855097870
C	-0.79347949069169	-3.42720072874273	2.24814558557905
H	-0.80418333091106	-1.33738665530194	1.44920782834833
H	-0.25066095602517	-3.33192838585576	3.17884450236375
Ti	0.42791118789743	-3.79444464645523	0.25521978513715
C	1.98243149377991	-4.75472468501832	-1.31592261395320
C	2.44818244837996	-5.11486039305699	-0.03300690092435
C	0.69455060848001	-5.33219022389147	-1.50001306809805
C	0.38527194498184	-6.06529311275612	-0.32210961389875
C	1.45701823173093	-5.90584669617527	0.60083195882128
H	0.07316620791588	-5.23969316746146	-2.38049069174529
H	2.49203300252744	-4.11399645142446	-2.02080483274489
H	3.37907033079871	-4.79710895585108	0.41617738176271
H	-0.51509956747099	-6.63671795180425	-0.15019868028044
H	1.51349788940461	-6.32546796316073	1.59644709451714
H	-2.35131353980864	-5.03397713637704	-0.23372107904336
H	-1.18718528948442	-5.61547062981787	2.12674011230555
Cl	0.76524033751794	-1.88059417141432	-1.29347564739257
C	6.30944736582079	-0.74313001378978	-1.35441890587466
C	6.89970507213426	-0.23801030989117	-2.51454474863095
C	5.00000586391984	-1.23404418932736	-1.38264544231663
C	6.18250481995196	-0.21759446736663	-3.71189248850870
C	4.87281749413505	-0.70388709503196	-3.74507020681436

C	4.28371327897579	-1.20854934857087	-2.58636087854343
H	6.86873832948681	-0.75964230445701	-0.42227522596840
H	6.64137282412536	0.17399889638197	-4.61531783967253
H	7.91837357639858	0.13792801155889	-2.48206705467223
H	3.26151954975888	-1.57983963380114	-2.60703606041817
H	4.31055708244860	-0.69201152112598	-4.67457847866737
C	4.35286561914946	-1.76678140774448	-0.12480878532726
C	3.35932869222911	-0.73929688586889	0.46331218787076
H	5.11216444570730	-2.01262156625961	0.62193392668592
H	3.76734787307767	-2.66193404417146	-0.34491759832961
S	2.44039561896458	-1.43324576870856	1.91383642488985
C	3.97636906325048	0.51290572861086	0.85851006761715
H	2.53551583804917	-0.56257748727696	-0.24557308483922
N	4.48959717280038	1.50166054606327	1.18259097239860
O	1.48135324288850	-0.43324528216257	2.34853594324807
O	1.97832387276550	-2.77007331263425	1.49964882938685
C	3.65112851371407	-1.70532347973311	3.16727042227048
C	4.28647941918017	-2.94753415718832	3.25280855879533
C	3.95861635354844	-0.64876316857230	4.02998535887548
C	4.93486277335153	-0.85044628021037	5.00300414056185
C	5.25794497018950	-3.13079679511798	4.23382502133349
H	4.01241392311989	-3.75109564010266	2.57906609260190
H	3.43800933327547	0.29843170041565	3.94600385186978
C	5.58195670977848	-2.08471556724790	5.10173619817767
H	5.75743587012512	-4.09016914299936	4.32171866501537
H	6.34048150194581	-2.23428334173881	5.86406844647256
H	5.18593832004224	-0.04457698150248	5.68500823718633

7-Cp*

C	-1.15266859408799	-2.25323977930958	0.97906523137778
C	-1.77619756934321	-2.97715928108480	-0.08615438967844
C	-1.99492138346203	-4.30819700268087	0.35448512272961
C	-1.47893789613358	-4.42504569504585	1.68386664982643
C	-0.96203550395161	-3.15965891120617	2.06773152858057
Ti	0.42028685523234	-3.86521209256720	0.22509334411581
C	2.04592489674580	-4.95986203668453	-1.30209374977424
C	2.53788270478440	-5.15620477177609	0.01128009241232
C	0.76420942141625	-5.57746060604954	-1.39754906736782
C	0.49713704710968	-6.21486901035641	-0.14418028664577
C	1.56422469838793	-5.89323434942205	0.75285788771214
Cl	1.01928458886139	-2.05744538039175	-1.35466168962045
C	6.21461219165547	-0.12351168305654	-1.18826927596159
C	6.75086127866089	0.56754666780861	-2.27615258512438
C	4.96242148078294	-0.73959570331530	-1.28690966832587
C	6.03651867858489	0.64933527243344	-3.47281493365997
C	4.78541303898563	0.03599277944978	-3.57708327664365
C	4.25000096094233	-0.65435212830557	-2.48996419860707
H	6.77220746414299	-0.18703773742457	-0.25696645597869
H	6.45251775109857	1.18702718654153	-4.32018235313999
H	7.72475704114284	1.04078974327179	-2.18830048202762
H	3.27529578908001	-1.13002564479860	-2.56532608975767
H	4.22636902226099	0.09374143224531	-4.50689410709232
C	4.37458701391159	-1.46634762109801	-0.09906107731460
C	3.29401525515386	-0.61589252787640	0.60303709913448
H	5.16127273033062	-1.72717639665148	0.61378581444203
H	3.86570200676746	-2.37806403632390	-0.41686829734722
S	2.58578986172840	-1.46120801425348	2.08890474941131

C	3.72183666162509	0.72680911334095	0.95187272567876
H	2.39837055873978	-0.57246703571268	-0.04094788039338
N	4.07770073220057	1.79921523626912	1.21583831744058
O	1.80029044279235	-0.46180608677005	2.79659106293641
O	1.94541509014327	-2.69010936693554	1.60192139299181
C	3.93373197292157	-1.96682073664230	3.12509444301681
C	3.94397427034587	-3.27473029846554	3.61356259313494
C	4.90181482237906	-1.03077544839221	3.50283820092922
C	5.91964909225620	-1.43429663632216	4.36390398056518
C	4.95722917636324	-3.65591975831832	4.49061550281680
H	3.16926416465244	-3.97064566430084	3.31682697184161
H	4.86611548411321	-0.00876985979312	3.14255819383429
C	5.94708877120785	-2.74167221351659	4.85631888171550
H	4.97327379370369	-4.66786995915637	4.88259357065842
H	6.74014977818102	-3.04618840033003	5.53226231765400
H	6.68529216387217	-0.72272265740205	4.65558251914829
C	-0.02003952588390	-5.69950006403115	-2.67250603615630
H	-0.15543294856344	-4.72310867548986	-3.15035252819603
H	-1.00787727672380	-6.13109265025186	-2.49822565345978
H	0.49955860865479	-6.34823736582580	-3.39094505554294
C	2.80050797542307	-4.38612581349049	-2.46092782454996
H	2.12866997490412	-3.89351619067361	-3.16675232456471
H	3.32470395039653	-5.19205496169219	-2.99436495950737
H	3.54677345529205	-3.65250140186092	-2.14862563712168
C	3.92962437798449	-4.85735957984336	0.48079722334266
H	4.57339446465785	-4.58013495167056	-0.35771767218678
H	4.36239618825911	-5.74834807991320	0.95287449747578
H	3.97603160293482	-4.05453598284610	1.21952417437743
C	1.77415382671545	-6.45091577171300	2.13356228545609
H	1.04823736162657	-7.23781021485856	2.35374609430942
H	1.68201611147384	-5.69066364176135	2.91863093812037
H	2.77652506799050	-6.88869329315453	2.22488381696212
C	-0.47629964494468	-7.33843648207854	0.05518969361983
H	-1.27418468990169	-7.32094250193655	-0.68729312733973
H	-0.92600399220855	-7.35346506730131	1.04771921427626
H	0.06373765786358	-8.28742630257582	-0.07073961003175
C	-1.68730857860993	-5.55404068760369	2.65242703973697
H	-2.28831520376583	-6.35462891418626	2.21647181027993
H	-2.22607959508179	-5.18180851951244	3.53360105465104
H	-0.75019416062089	-5.98811739248317	3.01293397884114
C	-0.48410149850824	-2.84278222831624	3.45415505479955
H	-0.08746240676813	-1.82954594690731	3.52174909760207
H	0.29761774261060	-3.53483052168547	3.78647957920181
H	-1.31683203190249	-2.93020641718885	4.16582481465992
C	-0.96136774452821	-0.76662132974745	0.99591338595006
H	-0.48978616863091	-0.41816131739754	0.07219237711844
H	-0.34482529257734	-0.44827962978536	1.83693845399205
H	-1.93664093333804	-0.26833602438790	1.08428928042374
C	-2.29256733380179	-2.39308644373429	-1.36628338852560
H	-2.25861472697648	-3.12231165236110	-2.18149787012106
H	-1.70348994210095	-1.52315458812777	-1.66482514059036
H	-3.33885493211946	-2.07515407599739	-1.24889308306999
C	-2.90054102271929	-5.27433408990045	-0.35207415757496
H	-2.60454404983256	-5.45063829229334	-1.38977101536162
H	-3.91583745297054	-4.85621805972601	-0.37830042200063
H	-2.95364501999517	-6.23605777531244	0.15877331106007

Coordinates of Optimized Structures in Toluene

1a

C	-4.21938356706201	3.94055856687111	-1.38443083516344
C	-4.87851013186707	5.14734742536179	-1.14755491515431
C	-4.76484946150761	2.73397663535582	-0.93200721701422
C	-6.09093508426807	5.15937818091218	-0.45623459353581
C	-6.64276344852447	3.95913129931025	-0.00340319201964
C	-5.98170326475960	2.75420746753971	-0.23934170806680
H	-3.27565605282043	3.93473615335819	-1.92381640073664
H	-6.60532687910092	6.09853243693078	-0.27388005542610
H	-4.44513249514303	6.07733773755553	-1.50425691283104
H	-6.41508079000791	1.81994289941293	0.11092798732991
H	-7.58935369296522	3.96068679993956	0.52959949885440
C	-4.04010869567260	1.42618648030241	-1.15167935808737
C	-3.11501581174171	1.09440714381899	0.04519971386254
H	-3.43948157499427	1.46326115920527	-2.06459822984541
H	-4.75390796619857	0.60266506254894	-1.23761273389714
S	-2.51864301008534	-0.66611132590944	-0.00425294606253
C	-1.97372347699332	1.98746923869725	0.14950352700548
H	-3.67786567562997	1.10857061045051	0.98661605899328
N	-1.05505615017557	2.69444662917068	0.19358701736399
O	-1.67069238089296	-0.85288065040430	1.16209441380571
O	-3.71843473799725	-1.47049190221028	-0.18811543523109
C	-1.52601224804318	-0.76104250977309	-1.46954236948167
C	-2.12483172159847	-1.14527816098190	-2.67231258320623
C	-0.16826274212938	-0.44052639283765	-1.38697444136919
C	0.60355584569289	-0.50487806337869	-2.54505186480714
C	-1.33830829874915	-1.20637261618621	-3.82068536687415
H	-3.17750131555661	-1.40534928723576	-2.69566819532642
H	0.26761016503495	-0.16008986344187	-0.43477228177031
C	0.01953913118327	-0.88411583923262	-3.75599929202541
H	-1.78380617835454	-1.51025096642534	-4.76280533983092
H	0.62784751423194	-0.93464200655213	-4.65416590532532
H	1.66069419669633	-0.26321234217266	-2.49976604412695

Cp_2TiCl

C	-2.31232506380365	0.41499562256674	0.85742988101882
C	-1.84932345695270	1.18471361304013	-0.24506069532683
C	-1.45029405279747	0.27511352255890	-1.26112382276401
C	-1.63600532421407	-1.04869543779111	-0.77539883813029
H	-1.82758761678594	2.26452668227048	-0.30838547329279
C	-2.17868132045133	-0.95736002275648	0.53058366489844
H	-2.65138658936185	0.80641107930673	1.80584912525638
H	-2.39938488022762	-1.78814875231685	1.18783108753964
Ti	-0.00003495586436	-0.00002857395025	0.57390553899434
C	1.63594643932428	1.04869578403116	-0.77534021765994
C	2.17861055770250	0.95737110517309	0.53065009509866
C	1.45028666198433	-0.27511040594390	-1.26108645819093
C	1.84931018827731	-1.18470032340309	-0.24501906710100
C	2.31225027444510	-0.41499117069330	0.85750346544831
H	1.05504429606241	-0.54725622313390	-2.22935067049041
H	1.40066179115559	1.96185041050338	-1.30596314294736
H	2.39941313754059	1.78826709374823	1.18774809049490
H	1.82769202062580	-2.26450845080389	-0.30838620516012
H	2.65169021304907	-0.80643010982193	1.80579191681431
Cl	-0.00006303747801	-0.00004164077517	2.91330813114516

H	-1.05500704525712	0.54714927181906	-2.22939312960820
H	-1.40081223697287	-1.96182307362803	-1.30609337603703

Cp^*_2TiCl

C	-0.68063081327853	1.87381489440576	1.42806593592391
C	-1.89367025972615	2.18767455644267	0.74765467944098
C	-2.78313976833221	1.08439961263297	0.89523043730121
C	-2.11789583026197	0.09135630737696	1.68093111911264
C	-0.79907619243564	0.55892504627133	1.96837402590222
Ti	-0.91726326605146	0.32549917798426	-0.37808860179505
C	-0.70452016600874	-0.67919457674615	-2.54127325883332
C	0.18470471733653	-1.36045134336327	-1.65993130865782
C	-2.03708871464841	-0.88075247877554	-2.07517231179399
C	-1.97133648153736	-1.74693891314021	-0.94057048673972
C	-0.59396907672715	-2.01168297889132	-0.65934813244267
Cl	0.60559015782737	1.84120339520765	-1.35171730699164
C	-0.33216031848804	0.04426751639552	-3.79912846677345
H	-0.95914111616555	0.92666299656069	-3.95714522043497
H	-0.46647532032950	-0.62092489352706	-4.66340867487430
H	0.70873676961200	0.37238054368161	-3.77781807428689
C	-3.28721068795403	-0.44605898020141	-2.78488255431526
H	-3.17189315723146	0.54796090069241	-3.22847221901601
H	-4.14303604036573	-0.41643864474761	-2.10610472703786
H	-3.53535599122161	-1.14127851145927	-3.59929448604392
C	-3.14422771376146	-2.50435615834161	-0.39323351303860
H	-4.02189413573778	-1.87301687243677	-0.23363263895899
H	-2.90770991478133	-3.00986606423094	0.54347377447992
H	-3.43333244985181	-3.27729880637838	-1.11863340852593
C	-0.05874147713067	-2.95187115105062	0.38144806240096
H	0.07965885340259	-3.95770124388319	-0.03840362486150
H	-0.73795381292114	-3.04389896091016	1.23253242386153
H	0.91251011687202	-2.61945391099223	0.76036999598974
C	1.67878347777014	-1.41063582928324	-1.77832290535031
H	2.06518513133063	-0.50593424754895	-2.25410353025187
H	1.99025026450632	-2.27474385810919	-2.38076298219872
H	2.15315311494383	-1.50558150759925	-0.79644735285644
C	0.20215612150408	-0.11713517215737	2.86056999463965
H	0.08134080928917	-1.20359094086872	2.84608760718069
H	0.08532701887457	0.21398493072786	3.90228058044533
H	1.22793826515919	0.11358670890213	2.55715892099697
C	-2.76448996882326	-1.06777061466366	2.37908220902615
H	-3.72882921312132	-1.32724726032570	1.94115349714880
H	-2.94370313312775	-0.79238308280532	3.42747609827905
H	-2.13643888295367	-1.96229562273224	2.38736999213728
C	-4.21901748752547	1.06644771887360	0.45851991660052
H	-4.35437813427591	1.57910715185014	-0.49837602091007
H	-4.85243895642434	1.57715384348106	1.19667393010176
H	-4.59890716830424	0.04771182067608	0.35190715494027
C	-2.19211109979228	3.46842656329307	0.02623447314568
H	-2.90659562100754	3.30970212431634	-0.78771403030012
H	-1.28260202989447	3.89842604210208	-0.40140758143192
H	-2.62988567998098	4.20749122244405	0.71097757033916
C	0.47980301595182	2.79538654318611	1.64750249754759
H	0.52059198906401	3.57559128722013	0.88516827602162
H	1.43212761698728	2.25761984482094	1.62776116647253
H	0.38693263974757	3.27817187562397	2.63021907928518

6-Cp

C	-0.92609807599855	0.35132040467790	2.28184553932623
C	-2.03817261353200	0.76559078750196	1.50257217940548
C	-2.42692728362744	-0.31865851086509	0.68627871982788
C	-1.56898830290479	-1.42081945510645	0.97733728394855
H	-2.46397799367886	1.75799047482399	1.48765085541510
C	-0.64780827483201	-1.01143116522989	1.96447063585481
H	-0.36987207255933	0.97290893078116	2.96921415863968
H	0.14788007133270	-1.61620776629403	2.37788335039674
Ti	-0.21029331500690	0.30478425714276	0.04063654316392
C	0.48683866465847	0.90406990277175	-2.19604558639310
C	1.29938481891379	-0.17339645419045	-1.78521867829233
C	-0.86084787527038	0.46053212379906	-2.22892949132588
C	-0.86864457838548	-0.91313259122893	-1.85981952207639
C	0.46329426984692	-1.30190430379997	-1.55784097003051
H	-1.72259961472081	1.06047725332514	-2.48815988459137
H	0.82189214096869	1.91195801666714	-2.38271918800976
H	2.36802211500528	-0.13059231361269	-1.62953897607314
H	-1.74129658037093	-1.54783269560350	-1.80576987126041
H	0.78251779704452	-2.27994777971010	-1.22589464761699
H	-3.22518046587513	-0.30992169693401	-0.04386492541526
H	-1.60133824336222	-2.39414112950997	0.50757229763893
Cl	0.00424550325634	2.74651603612701	0.21712707781214
C	3.05319827390682	-2.99060524618060	0.79626932988355
C	2.18146722003036	-4.05394974713473	1.03099876997104
C	3.82739406283751	-2.46206995293978	1.83435471857222
C	2.07424919568820	-4.60010929171267	2.31041392439117
C	2.84939233886208	-4.08376062270381	3.35134215086770
C	3.72025658739061	-3.02097281801331	3.11403823716340
H	3.13330356161483	-2.56319446875513	-0.19988313951532
H	1.39415469323807	-5.42637055177502	2.49571487269076
H	1.58679614173678	-4.45616317653845	0.21557051245293
H	4.32060406530085	-2.62087558736146	3.92812373906741
H	2.77602606804818	-4.50970012668776	4.34789510204664
C	4.70300108908698	-1.25376501811984	1.59425084390782
C	3.98620988302683	0.01984384109095	2.11383653085758
H	4.90900221991137	-1.13809379090377	0.52637840704707
H	5.65486343188037	-1.32675906388435	2.12521944934257
S	4.99183725230321	1.57305285443415	1.84064434307340
C	2.68561779616690	0.18896950762356	1.51427152239222
H	3.88658228682774	-0.00539638889868	3.20537000059152
N	1.66390902223950	0.25490179965927	0.96522035884804
O	4.34654818189811	2.61283878085798	2.62630054289306
O	6.37792849147195	1.20130832188253	2.09015927232152
C	4.76863921114796	1.92064834669541	0.11786819398022
C	5.66673009422019	1.38164349110937	-0.80816453436903
C	3.67847470156939	2.70327136605655	-0.27236691141008
C	3.49224633377212	2.95252722244979	-1.62991328522510
C	5.46163526848816	1.63757758995466	-2.16230459823127
H	6.51259373292228	0.79394996305247	-0.46744991869189
H	2.98754544349128	3.09890717752248	0.46304919070079
C	4.37730554275974	2.42008847393590	-2.56940275727194
H	6.15063406768497	1.23304513847446	-2.89716929666715
H	4.22331895623806	2.61689364082893	-3.62628560549872
H	2.65051469333626	3.55951601044832	-1.94616686652655

6-Cp*

C	-0.87926454574905	0.92595204759691	2.24805357752718
C	-1.98467563656628	1.35355921025597	1.45141917152303
C	-2.62609078594238	0.20086871604535	0.92830460216301
C	-1.93152370557467	-0.95310822099675	1.41616719532238
C	-0.85732228118604	-0.50512024214189	2.22835669883130
Ti	-0.35880700411213	0.27817327557575	0.01119380781588
C	0.23804140432456	0.48835210131246	-2.38252062559684
C	1.08427766823588	-0.48470348879475	-1.79377551791557
C	-1.10506649566771	0.03908621990501	-2.26956832868067
C	-1.08066842672923	-1.26519998613413	-1.67474992932132
C	0.26909638410334	-1.57147937853439	-1.33595856034430
Cl	0.26235222735395	2.63723385658452	-0.24492329813728
C	4.46672637495589	-3.34781683388403	-0.05289049431308
C	3.99395466464171	-4.64477638402219	-0.25138900113745
C	4.20040464821303	-2.66195023879527	1.13780021107167
C	3.23782206212214	-5.27249614042060	0.73872698663247
C	2.96512544397804	-4.59704250984986	1.92909927440339
C	3.44570779093649	-3.30297464773357	2.12796864624125
H	5.04575792142224	-2.85769053012653	-0.83187153149673
H	2.85898934454280	-6.27829231411156	0.58233307507740
H	4.20695692998221	-5.15913027438692	-1.18413501766731
H	3.22510633478301	-2.79577991619376	3.06322606768566
H	2.37444781259403	-5.07558371989407	2.70507611843456
C	4.75344281657327	-1.26514725355127	1.32940989830491
C	3.78526879367840	-0.32173631910876	2.06833022626227
H	4.99853295499236	-0.83358382481547	0.35444248752100
H	5.67677159733261	-1.29301754752795	1.91807559284800
S	4.59841414105464	1.34309718652361	2.38714414304904
C	2.51777130810709	-0.14011767138734	1.40896003863407
H	3.61562800959549	-0.65547857981855	3.09948073618451
N	1.49299421352398	0.03026572659217	0.87606154176212
O	3.64043524621027	2.11433967154392	3.16513662272454
O	5.91952035479510	1.04294408004410	2.92817400560079
C	4.76576862647569	2.06091921967695	0.77953978593737
C	5.95729864002252	1.87987879670514	0.06963846708763
C	3.67882792063986	2.75448798526098	0.24206248122030
C	3.79690073788866	3.28387741432855	-1.04188899956848
C	6.05841646276264	2.41863992092464	-1.21069070945415
H	6.78350455370599	1.34330346842961	0.52372642479107
H	2.75620465286682	2.86773258058681	0.79954872388730
C	4.98030127798162	3.11755378978796	-1.76315396470241
H	6.97784037375976	2.29684179720223	-1.77500375514192
H	5.06537552995363	3.53409848862132	-2.76264814089171
H	2.95453110973553	3.81634354691698	-1.47063777659144
C	0.01980449073093	-1.40213506051207	3.05044835221649
H	0.42958065087370	-2.22808525444702	2.46034887285828
H	-0.55404849758709	-1.83917571531921	3.87901154642103
H	0.85376981716966	-0.84792757957112	3.48798085943848
C	0.00636156811912	1.82072463275515	3.05923893384404
H	0.21054229704187	2.75101196364759	2.52221789654314
H	0.96709009511058	1.35049170379064	3.28237766491582
H	-0.47165312765097	2.07255714137374	4.01587770406166
C	-2.49224733451176	2.75666023083276	1.31819461730809
H	-2.86545580801272	2.95752657002411	0.30959274512340
H	-1.70558727466268	3.48166218387333	1.53198951967789
H	-3.32000594187681	2.92302783188343	2.02247273982944

C	-3.95326292477684	0.22805394711377	0.23024738643723
H	-4.16206276146565	-0.69904234904840	-0.30647597120761
H	-4.02153587102361	1.05861237990949	-0.47717215661293
H	-4.75635405823870	0.36453071905928	0.96792750122644
C	-2.41571241337676	-2.37327687345490	1.39733080291255
H	-3.24047967337729	-2.51687125844592	0.69875455201893
H	-2.78105280444117	-2.64100103213786	2.39854445481644
H	-1.62364049210254	-3.08568264088859	1.14710297234221
C	-2.19582896890429	-2.26280637207533	-1.76717286409367
H	-2.25124448236825	-2.62694853856924	-2.80260530480019
H	-3.17248868467167	-1.83990948032220	-1.52561766205310
H	-2.02484762376398	-3.12921247000505	-1.12772459505612
C	0.78383739667934	-2.86089300261818	-0.76857248042042
H	1.18303308085459	-3.51042421559272	-1.55903162331271
H	-0.00569931644924	-3.41581729798918	-0.25537067105390
H	1.59260551650787	-2.69165788330645	-0.05162784564182
C	2.58235436408647	-0.44806629776370	-1.80416520801953
H	2.96670010722820	0.53234915937273	-1.50725864971367
H	2.96052481051653	-0.66372100493635	-2.81246111047888
H	2.99638259897482	-1.20255432747547	-1.13145840749765
C	0.69420607343951	1.69477846343990	-3.13911988204890
H	-0.03688071375139	2.50391530555683	-3.08457713525350
H	0.83619237678610	1.42866793616348	-4.19693223423577
H	1.64249494814266	2.07228431960998	-2.75129099504755
C	-2.29548251496891	0.72789060278494	-2.87066002930956
H	-2.31195239565273	1.79248281450206	-2.61357147835774
H	-3.23101841383559	0.28046792373783	-2.52836852323375
H	-2.27537554710947	0.65545974685665	-3.96681525212600

7-Cp

C	-1.07680888849720	-2.35192820773401	1.30230680448070
C	-1.76719682076202	-2.90865692805008	0.19098524163696
C	-1.90019673931757	-4.29537607705062	0.41215424015296
C	-1.30827228204592	-4.60195683484166	1.67298079553832
H	-2.07933782454586	-2.37168569802657	-0.69302951563902
C	-0.80860840734860	-3.40268294736206	2.22559302214855
H	-0.78022263242878	-1.31911709152756	1.41172433577501
H	-0.27993954916292	-3.30658863781331	3.16420250964813
Ti	0.43655386850779	-3.80152439284963	0.25146874240318
C	1.99748882504089	-4.79900879617027	-1.29679863912072
C	2.45001640552806	-5.14600073973337	-0.00608582599508
C	0.70596896882661	-5.36608335153625	-1.48258464010432
C	0.38144958490648	-6.08132175487596	-0.29804428675343
C	1.44715121860984	-5.91992492015099	0.63090633935429
H	0.09278266056447	-5.27714866424965	-2.36889356640806
H	2.51578375275177	-4.17022689074943	-2.00581607135973
H	3.38071675074987	-4.82916630624181	0.44409132929291
H	-0.52366058922422	-6.64548410672695	-0.12722360040982
H	1.49420428261120	-6.33088661109325	1.63056312842236
H	-2.35539747219322	-5.00451706608117	-0.26601939446834
H	-1.23267033255878	-5.58552144306679	2.11622098254140
Cl	0.81371155045880	-1.92037061344147	-1.30115044699285
C	6.27564903750862	-0.71755164914671	-1.34750823434136
C	6.86965478574402	-0.19991984703504	-2.49942081885596
C	4.97537196508399	-1.23085962047869	-1.39099736557803
C	6.16546718248529	-0.18943410440745	-3.70417484538530
C	4.86517651552114	-0.69758660231887	-3.75256475908230

C	4.27169896129226	-1.21480747619875	-2.60191742123955
H	6.82423575664076	-0.72354023491792	-0.40881756089064
H	6.62720655715554	0.21273883088942	-4.60146123945864
H	7.88065029444584	0.19490685985271	-2.45469844240732
H	3.25530165351631	-1.60081772910961	-2.63293391926223
H	4.31234033406675	-0.69215564181962	-4.68771456073514
C	4.32173731700559	-1.77475209068052	-0.14148171835741
C	3.35162646319173	-0.73987538134481	0.47275844129812
H	5.07903381300243	-2.04974628040618	0.59788094328599
H	3.71579541191375	-2.65229862034224	-0.37644600283846
S	2.44162657415344	-1.44537068692229	1.92359556743643
C	3.99318596224525	0.49421488202360	0.88643258638963
H	2.52340149201829	-0.54302135856899	-0.22528076445457
N	4.52697777705909	1.46437522008787	1.23291188229041
O	1.48037960161807	-0.45567102804534	2.37243313789995
O	1.98891057095460	-2.78791551952376	1.51488839801690
C	3.66496514614238	-1.71162745127643	3.16991957702254
C	4.30321033876055	-2.95177017690602	3.25764214475578
C	3.97558262476474	-0.65248388403220	4.02747301774508
C	4.95790635935263	-0.84832938106878	4.99554140418330
C	5.28041844247262	-3.13030604154144	4.23378238760259
H	4.02363830127091	-3.75705776180147	2.58809316181857
H	3.45276253539803	0.29315613890637	3.94051197395693
C	5.60779723100830	-2.08058005038401	5.09570063805266
H	5.78210722522072	-4.08858261323574	4.32319540393559
H	6.37109656565020	-2.22583458053348	5.85423638760085
H	5.21226087286491	-0.03888804034023	5.67214911545203

7-Cp*

C	-1.14999215821318	-2.25024138953009	0.98217742896997
C	-1.77342991003036	-2.97571834389781	-0.08178755697301
C	-1.98837946781994	-4.30714624639945	0.35928575053047
C	-1.46769474969377	-4.42308458093847	1.68681780069497
C	-0.95447365305416	-3.15609774310349	2.06992547638503
Ti	0.42536744128608	-3.86345943047206	0.22275095938703
C	2.04365888478076	-4.95544559404948	-1.32156351441450
C	2.54459836047078	-5.15616218588716	-0.01265090704164
C	0.76071944237363	-5.57057765437865	-1.40917497244358
C	0.50149583916956	-6.21145184212221	-0.15594160184103
C	1.57529938823926	-5.89407729979447	0.73405189976692
Cl	1.02017983992936	-2.05636959074516	-1.34050982201160
C	6.20812234835465	-0.17788633057751	-1.19549099546509
C	6.75652579339967	0.50343567696578	-2.28274161708511
C	4.94441131202490	-0.76950880583146	-1.29319761320272
C	6.04278994309266	0.60016417245359	-3.47809875704758
C	4.78007821914594	0.01213400196662	-3.58131213954547
C	4.23220941796238	-0.66888655256508	-2.49498077398328
H	6.76422911099520	-0.25063093641354	-0.26393139856075
H	6.46810168461960	1.13099059218374	-4.32515545825164
H	7.73894524779669	0.95869474003706	-2.19514137269189
H	3.24809785531309	-1.12483630397866	-2.56925355059565
H	4.22053966498517	0.08274279546764	-4.50986907065923
C	4.34298535746893	-1.48727584358135	-0.10700570595175
C	3.29373004666932	-0.61265241182594	0.61172261114210
H	5.12613349315986	-1.77668616494570	0.59949357137406
H	3.80315885584856	-2.37945376930556	-0.42924664510085
S	2.57950770106083	-1.45826107631815	2.09466475307083

C	3.76815509559364	0.71052503829043	0.97621199067829
H	2.39411280369511	-0.53752133717212	-0.02258130073853
N	4.16674008605507	1.76324624333006	1.25761422918867
O	1.78421032070545	-0.46502109825792	2.79655012941680
O	1.95892049107845	-2.69857354715468	1.60886959369806
C	3.92607184533498	-1.94772424168642	3.14487707767638
C	3.98737328677513	-3.27120430614030	3.58397196202990
C	4.83964033853605	-0.98349316187930	3.58339200718784
C	5.85691845154111	-1.37378436101117	4.45115658766407
C	4.99861229736776	-3.64011067135947	4.46872716669873
H	3.24962304134170	-3.98770050432627	3.24542942906928
H	4.75838111688014	0.04930542287497	3.26427145235414
C	5.93669516642362	-2.69704601846500	4.89196988485474
H	5.05278797156079	-4.66473394304580	4.82298496426317
H	6.72845186442922	-2.99118296134026	5.57423004580726
H	6.57996179715266	-0.63893140938213	4.79043007784513
C	-0.03237896777148	-5.68477337137305	-2.67911385130277
H	-0.16904116286126	-4.70540116908782	-3.15024578453260
H	-1.01974727913694	-6.11570271273135	-2.50040268939023
H	0.48070087723828	-6.33053052873489	-3.40485129566022
C	2.78739259472214	-4.37576747170546	-2.48420674270159
H	2.11240641162187	-3.85687529277576	-3.16774949862865
H	3.28605071138114	-5.18170847946465	-3.04160976156053
H	3.55332130682856	-3.66186022424923	-2.17365673441343
C	3.94005881141868	-4.86069706344734	0.44775207454544
H	4.57944248561695	-4.58829854414817	-0.39566782982980
H	4.37318554418638	-5.75176552176747	0.91963173406182
H	3.99373479529077	-4.05434171981803	1.18207781666510
C	1.79428879859268	-6.45579147476041	2.11159907126758
H	1.06710856760933	-7.24028193009879	2.33635443738752
H	1.71103425813378	-5.69638534608202	2.89872922928860
H	2.79534450908683	-6.89879352857535	2.19440612839384
C	-0.47037545828431	-7.33560680154625	0.04716551227953
H	-1.27107202493548	-7.31869507808963	-0.69234376150355
H	-0.91723928462086	-7.35006059087300	1.04121563123086
H	0.06872312899927	-8.28489430269711	-0.08063377081971
C	-1.67062446692268	-5.55283641957240	2.65578534278451
H	-2.27866004939392	-6.35052011610893	2.22379525547348
H	-2.20041274579841	-5.18146882238935	3.54270576750653
H	-0.73186808826154	-5.99152689274780	3.00693880523993
C	-0.47803988486173	-2.83751030046053	3.45632256601530
H	-0.08800499814483	-1.82201645485241	3.52547502051311
H	0.30825101882929	-3.52486298684433	3.78817214138750
H	-1.31027751776806	-2.93062608700330	4.16792353380449
C	-0.96472422878462	-0.76307559543028	0.99657070624680
H	-0.48688386600976	-0.41541954903217	0.07581349259634
H	-0.35854647673049	-0.43797589902890	1.84232616302023
H	-1.94362771460158	-0.27031145587940	1.07346273438274
C	-2.29037852436134	-2.39133752447089	-1.36143560273455
H	-2.27547834249639	-3.12641993144809	-2.17181899319145
H	-1.68816003703370	-1.53469853244594	-1.67160257453654
H	-3.32985037415256	-2.05421084769602	-1.23761555844428
C	-2.89791112653244	-5.27330065664097	-0.34196069311664
H	-2.61052445935656	-5.44743490574459	-1.38247321315378
H	-3.91391995283422	-4.85660035408611	-0.35937087991834
H	-2.94603807170778	-6.23623654177844	0.16728399519826

References

[1] J. Weweler, S. L. Younas, J. Streuff, *Angew. Chem. Int. Ed.* **2019**, *58*, 17700–17703.
[2] L. M. Schneider, V. M. Schmiedel, T. Pecchioli, D. Lentz, C. Merten, M. Christmann, *Org.Lett*. **2017**, *19*, 2310–2313.
[3] J. Gordon, S. Hildebrandt, K. R. Dewese, S. Klare, A. Gansäuer, T. V. RajanBabu, W. A. Nugent, *Organometallics* **2018**, *37*, 4801–4809.
[4] a) K. S. Pandit, R. V. Kupwade, P. V. Chavan, U. V. Desai, P. P Wadgaonkar, K. M. Kodam, *ACS Sustainable Chem. Eng.* **2016**, *4*, 3450–3464; b) Y.-Q. Yang, Z. Lu, *Chin. J. Chem.* **2014**, *32*, 650–653.
[5] T. Takuwa, T. Minowa, H. Fujisawa, T. Mukaiyama, *Chem. Pharm. Bull.* **2005**, *53*, 476–480.
[6] W. Ma, S. Cui, H. Sun, W. Tang. D. Xue, C. Li, J. Fan, J. Xiao, C. Wang, *Chem. Eur. J.* **2018**, *24*, 13118–13123.
[7] C. Wang, Q. Shang, R. Qi, H. Chai, H. Wang, M. Guo, Z. Xu, *Chem. Commun.* **2019**, *55*, 9991–9994.
[8] X. Yang, D. Nath, M. R. Gau, O. W. Steward, F. F. Fleming, *Angew. Chem. Int. Ed*. **2017**, *56*, 7257–7260.
[9] M. Fujii, K. Nakamura, H. Mekata, S. Oka, A. Ohno, *Bull. Chem. Soc. Jpn*. **1988**, *61*, 495–500.
[10] A. R. Katritzky, A. A. A. Abdel-Fattah, A. V. Vakulenko, H. Tao, *J. Org. Chem*. **2005**, *70*, 9191–9197.
[11] T. Sakamoto, E. Katoh, Y. Kondo, H. Yamanaka, *Chem. Pharm. Bull*. **1990**, *38*, 1513–1517.
[12] D. B. Ramachary, G. B. Reddy, *Org. Biomol. Chem.* **2006**, *4*, 4463–4468.
[13] D. Nath, F. F. Fleming, *Angew. Chem. Int. Ed.* **2011**, *50*, 11790–11793.
[14] A. Gansäuer, M. Behlendorf, D. von Laufenberg, A. Fleckhaus, C. Kube, D. V. Sadasivam, R. A. Flowers II, *Angew. Chem. Int. Ed.* **2012**, *51*, 4739–4742.
[15] a) J. Streuff, *Chem. Eur. J.* **2011**, *17*, 5507–5510; b) P. Bichovski, T. M. Haas, D. Kratzert, J. Streuff, *Chem. Eur. J.* **2015**, *21*, 2339–2342; c) P. Bichovksi, T. M. Haas, M. Keller, J. Streuff, *Org. Biomol. Chem.* **2016**, *14*, 5673–5682.
[16] a) A. Fürstner, A. Hupperts, *J. Am. Chem. Soc.* **1995**, *117*, 4468–4475; b) A. Gansäuer, *Chem. Commun.* **1997**, 457–458; c) A. Rosales, J. L. Oller-López, J. Justicia, A. Gansäuer, J. E. Oltra, J. M. Cuerva, *Chem. Commun.* **2004**, 2628–2629; d) J. Streuff, M. Feurer, P. Bichovski, G. Frey, U. Gellrich, *Angew. Chem. Int. Ed.* **2012**, *51*, 8661–8664.
[17] D. Zhang, Y. Huang, E. Zhang, R. Yi, C. Chen, L. Yu, Q. Xu, *Adv. Synth. Catal.* **2018**, *360*, 784–790.
[18] S. Kubosaki, H. Takeuchi, Y. Iwata, Y. Tanaka, K. Osaka, M. Yamawaki, T. Morita, Y. Yoshimi, *J. Org. Chem*. **2020**, *85*, 5362–5369.
[19] T. Moriya, K. Shoji, S. Yoneda, R. Ikeda, T. Konakahara, N. Sakai, *Synthesis* **2013**, *45*, 3233–3238.
[20] X. Mo, T. D. R. Morgan, H. T. Ang, D. G. Hall, D. G. *J. Am. Chem. Soc.* **2018**, *140*, 5264–5271.
[21] R. A. Gardner, J.-G. Delcros, F. Konate, F. Breitbeil III, B. Martin, M. Sigman, M. Huang, O. Phanstiel IV, *J. Med. Chem*. **2004**, *47*, 6055–6069.
[22] B. Anxionnat, D. G. Pardo, G. Ricci, J. Cossy, *Org. Lett*. **2011**, *13*, 4084–4087.
[23] R. Lerebours, C. Wolf, *Org. Lett*. **2007**, *9*, 2737–2740.
[24] A. M. Cardarelli, M. Fagnoni, M. Mella, A. Albini, *J. Org. Chem*. **2001**, *66*, 7320–7327.
[25] C. Chiappe, D. Pieraccini, P. Saullo, *J. Org. Chem*. **2003**, *68*, 6710–6715.
[26] R. Ding, Y. Liu, M. Han, W. Jiao, J. Li, H. Tian, B. Sun, *J. Org. Chem*. **2018**, *83*, 12939–12944.
[27] T. Wang, Y.-N. Wang, R. Wang, B.-C. Zhang, C. Yang, Y.-L. Li, X.-S. Wang, *Nat. Commun.* **2019**, *10,* 5373.
[28] A. Guđmundsson, K. E. Schlipköter, J.-E. Bäckvall, *Angew. Chem. Int. Ed.* **2020**, *59*, 5403–5406.

[29] The glassware was not put in an HCl bath after the base bath.
[30] The presence of Coll•HCl greatly facilitated the reduction, probably by activation of the Zn surface. Without Coll•HCl, the reduction of $Cp*_2TiCl_2$ to $Cp*_2TiCl$ in toluene was capricious and could not always be achieved.
[31] The $Cp*_2TiCl_2$ does not fully dissolve in toluene at room temperature (23 ºC). It dissolves, however, at elevated temperature to give a dark red solution.
[32] J. W. Pattiasina, H. J. Heeres, F. van Bolhuis, A. Meetsma, J. H. Teuben, A. L. Spek, *Organometallics* **1987**, *6*, 1004–1010.
[33] a) M. Kobayashi, K. Tanaka, H. Minato, *Bull. Chem. Soc. Jpn.* **1972**, *45*, 2906–2909; b) F. Bertrand, F. Le Guyader, L. Liguori, G. Ouvry, B. Quiclet-Sire, S. Seguin, S. Z. Zard, *C. R. Acad. Sci., Ser. IIc: Chim.* **2001**, *4*, 547–555.
[34] a) C. M. M. da Silva Corrêa, A. S. Lindsay, W. A. Waters, *J. Chem. Soc. C* **1968**, 1872–1874; b) J. E. Bennett, G. Brunton, B. C. Gilbert, P. E. Whittall, *J. Chem. Soc., Perkin Trans. II* **1988**, 1359–1364.
[35] See also: a) D. Marković, P. Vogel, *Org. Lett.* **2004**, *6*, 2693–2696; b) D. Marković, A. Varela-Álvarez, J. Angel Sordo, P. Vogel, *J. Am. Chem. Soc.* **2006**, *128*, 7782–7795.
[36] a) F. Neese, *WIREs* **2012**, *2*, 73–78; b) F. Neese, *WIREs* **2018**, *8*, e1327.
[37] a) K. Eichkorn, O. Treutler, H. Öhm, M. Häser, R. Ahlrichs, *Chem. Phys. Lett.* **1995**, *240*, 283–290; b) K. Eichkorn, F. Weigend, O. Treutler, R. Ahlrichs, *Theor. Chem. Acc.* **1997**, *97*, 119–124; c) R. Bauernschmitt, M. Häser, O. Treutler, R. Ahlrichs, *Chem. Phys. Lett.* **1997**, *264*, 573–578; d) P. Deglmann, K. May, F. Furche, R. Ahlrichs, *Chem. Phys. Lett.* **2004**, *384*, 103–107; e) C. K. Skylaris, L. Gagliardi, N. C. Handy, A. G. Ioannou, S. Spencer, A. Willets, *J. Mol. Struct. (THEOCHEM)* **2000**, *501*, 229–239.
[38] F. Neese, F. Wennmohs, A. Hansen, U. Becker, *Chem. Phys.* **2009**, *356*, 98–109.
[39] a) S. Grimme, A. Hansen, J. G. Brandenburg, C. Bannwarth, *Chem. Rev.* **2016**, *116*, 5105–5154. b) S. Grimme, J. Antony, S. Ehrlich, H. Krieg, *J. Chem. Phys.* **2010**, *132*, 154104.
[40] a) S. Grimme, S. Ehrlich, L. Goerigk, *J. Comput. Chem.* **2011**, *32*, 1456–1465; b) A. D. Becke, E. R. Johnson, *J. Chem. Phys.* **2005**, *123*, 154101; c) E. R. Johnson, A. D. Becke, *J. Chem. Phys.* **2005**, *123*, 24101.
[41] J. Tao, J. P. Perdew, V. N. Staroverov, G. E. Scuseria, *Phys. Rev. Lett.* **2003**, *91*, 146401.
[42] F. Weigend, R. Ahlrichs, *Phys. Chem. Chem. Phys.* **2005**, *7*, 3297–3305.
[43] F. Weigend, *Phys. Chem. Chem. Phys.* **2006**, *8*, 1057–1065.
[44] M. Cossi, N. Rega, G. Scalmani, V. Barone, *J. Comput. Chem.* **2003**, *24*, 669-681.
[45] Y. Zhao, D. G. Truhlar, *J. Phys. Chem. A* **2005**, *109*, 5656–5667.
[46] a) F. Weigend, F. Furche, R. Ahlrichs, *J. Chem. Phys.* **2003**, *119*, 12753; b) A. Hellweg, C. Hättig, S. Höfener, W. Klopper, *Theor. Chem. Acc.* **2007**, *117*, 587–597.

NMR Spectra

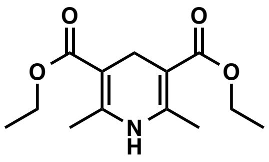

S1

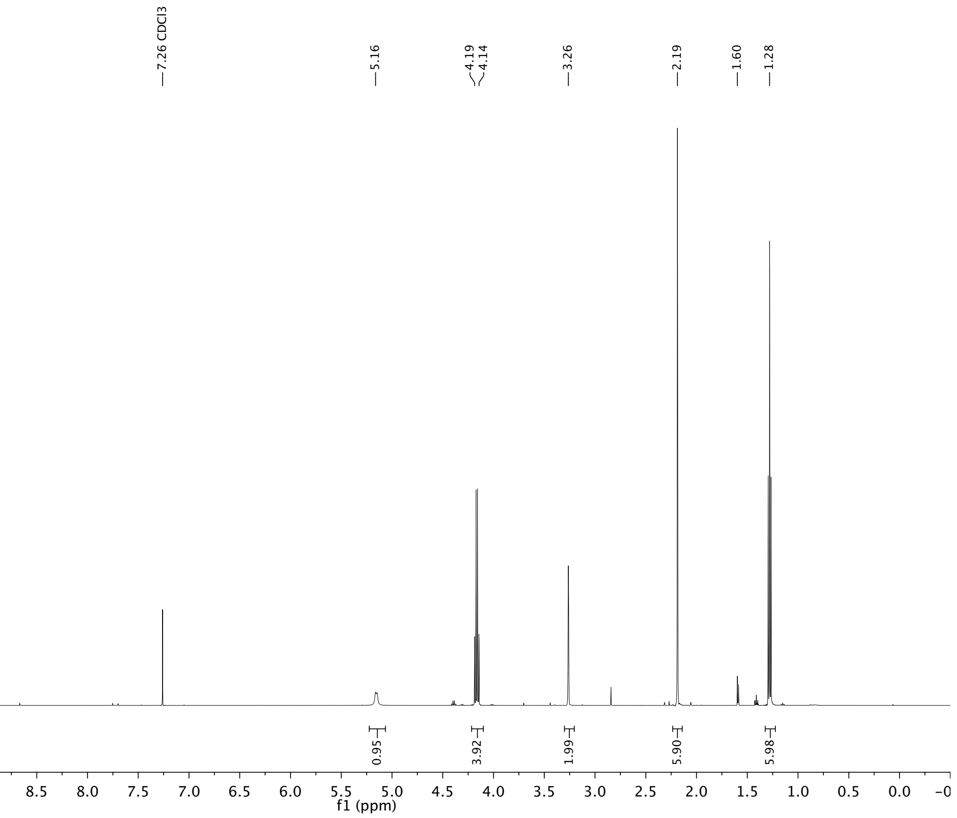

^{1}H NMR (500 MHz, $CDCl_3$) spectrum of compound **S1**.

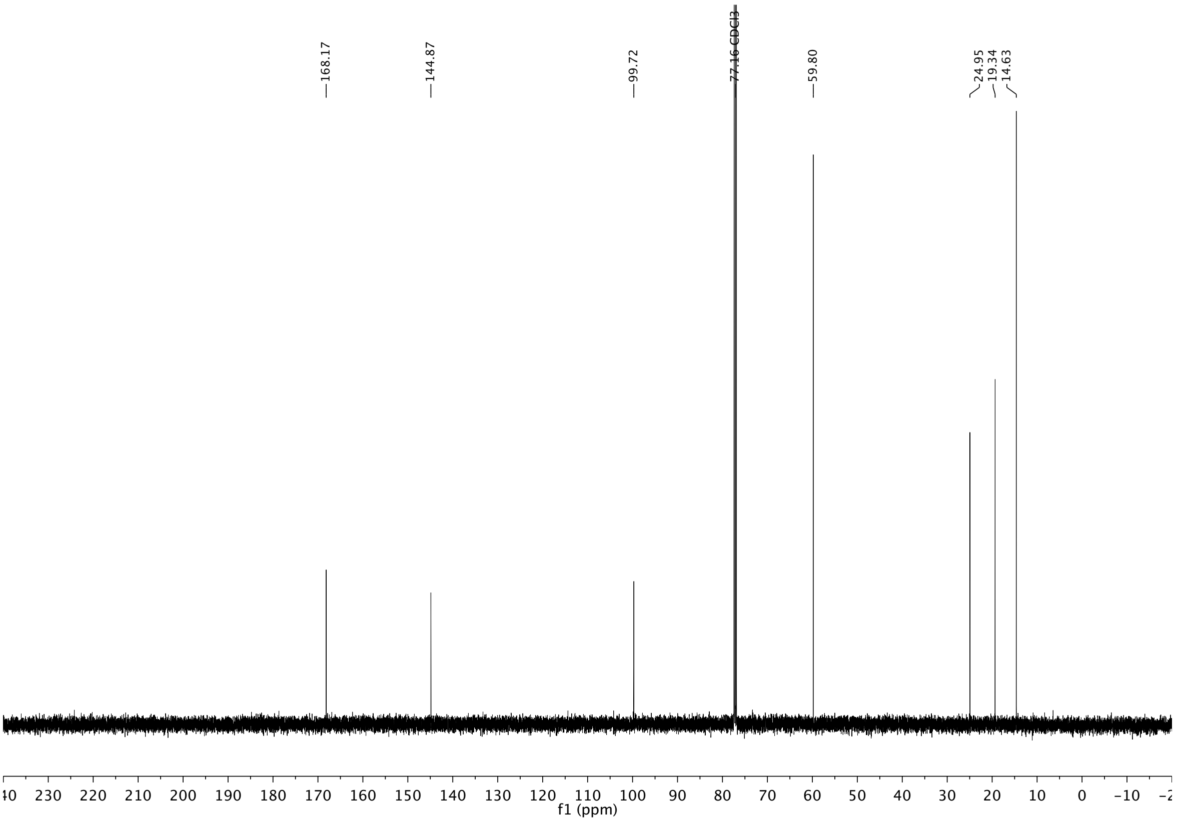

^{13}C NMR (126 MHz, $CDCl_3$) spectrum of compound **S1**.

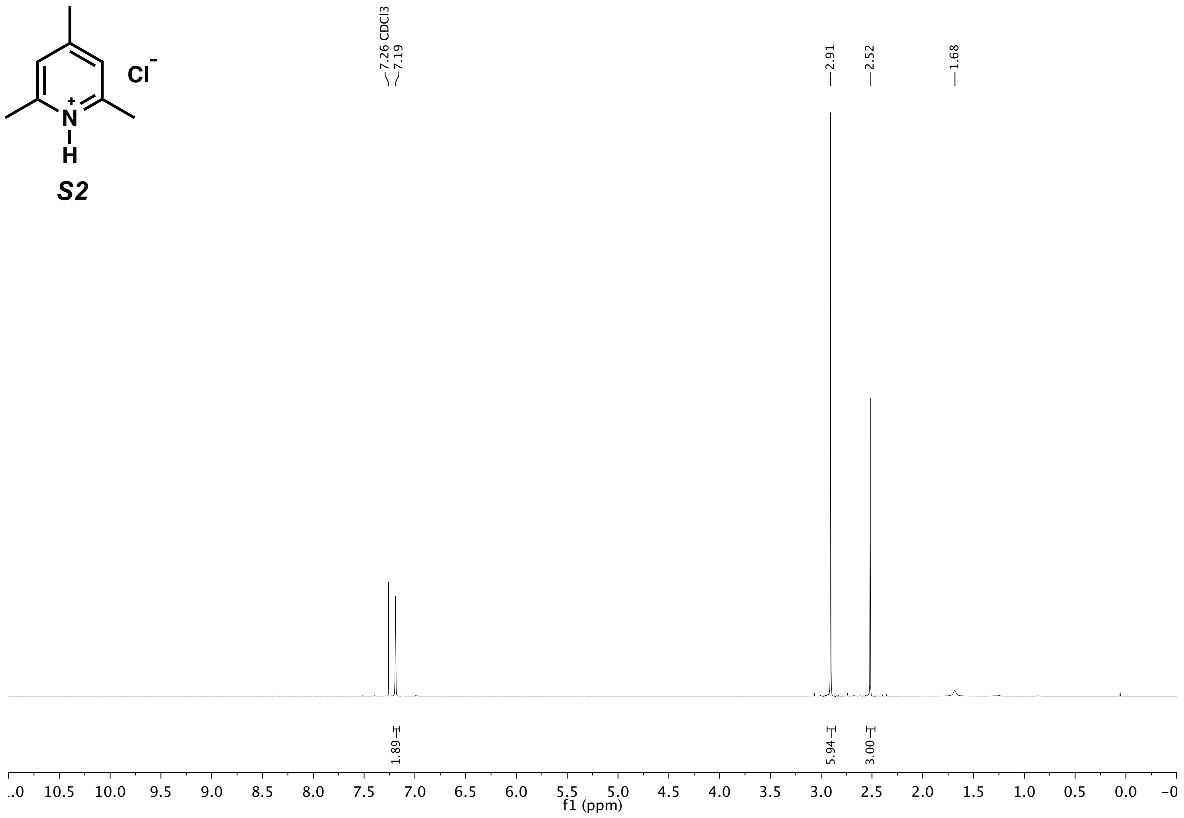

^{1}H NMR (400 MHz, $CDCl_3$) spectrum of compound **S2**.

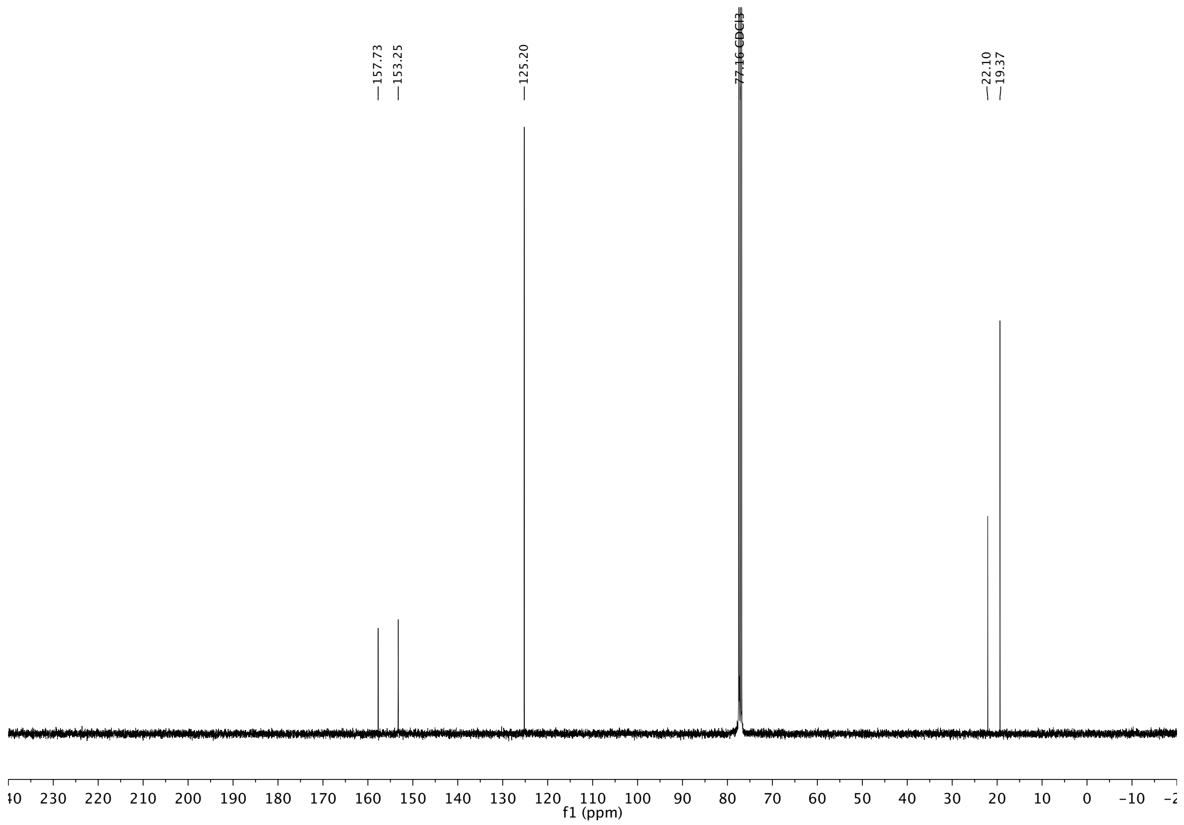

^{13}C NMR (101 MHz, $CDCl_3$) spectrum of compound **S2**.

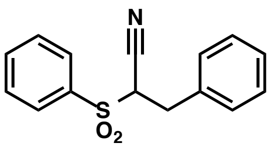

1a

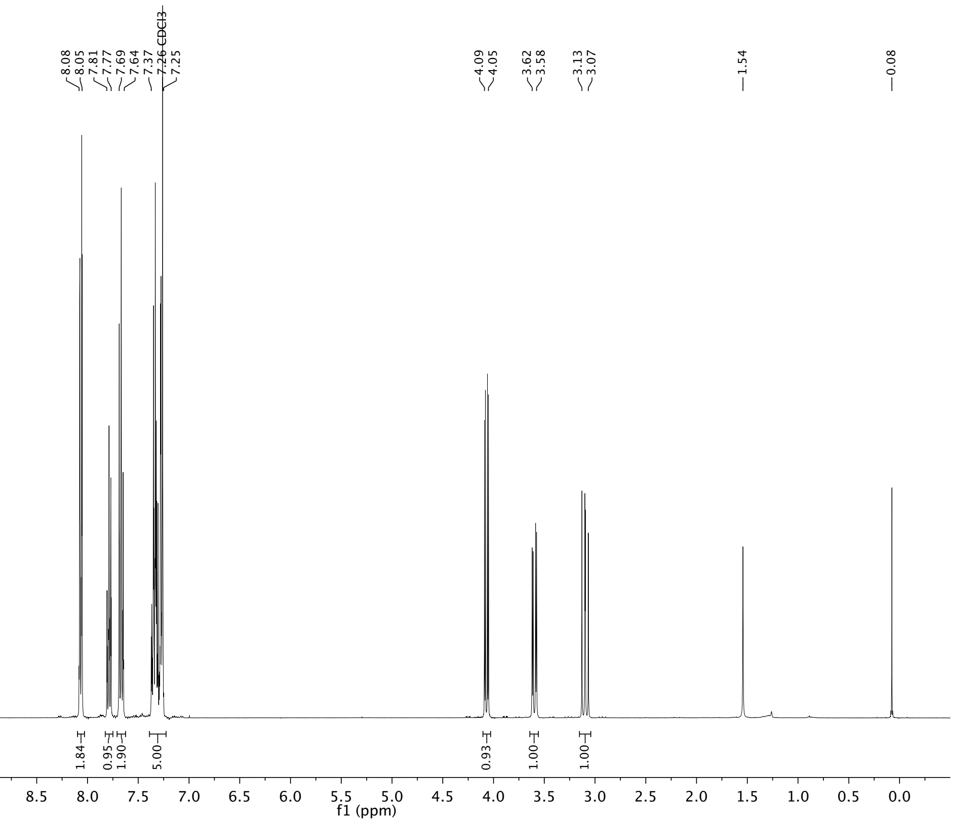

^{1}H NMR (400 MHz, $CDCl_3$) spectrum of compound **1a**.

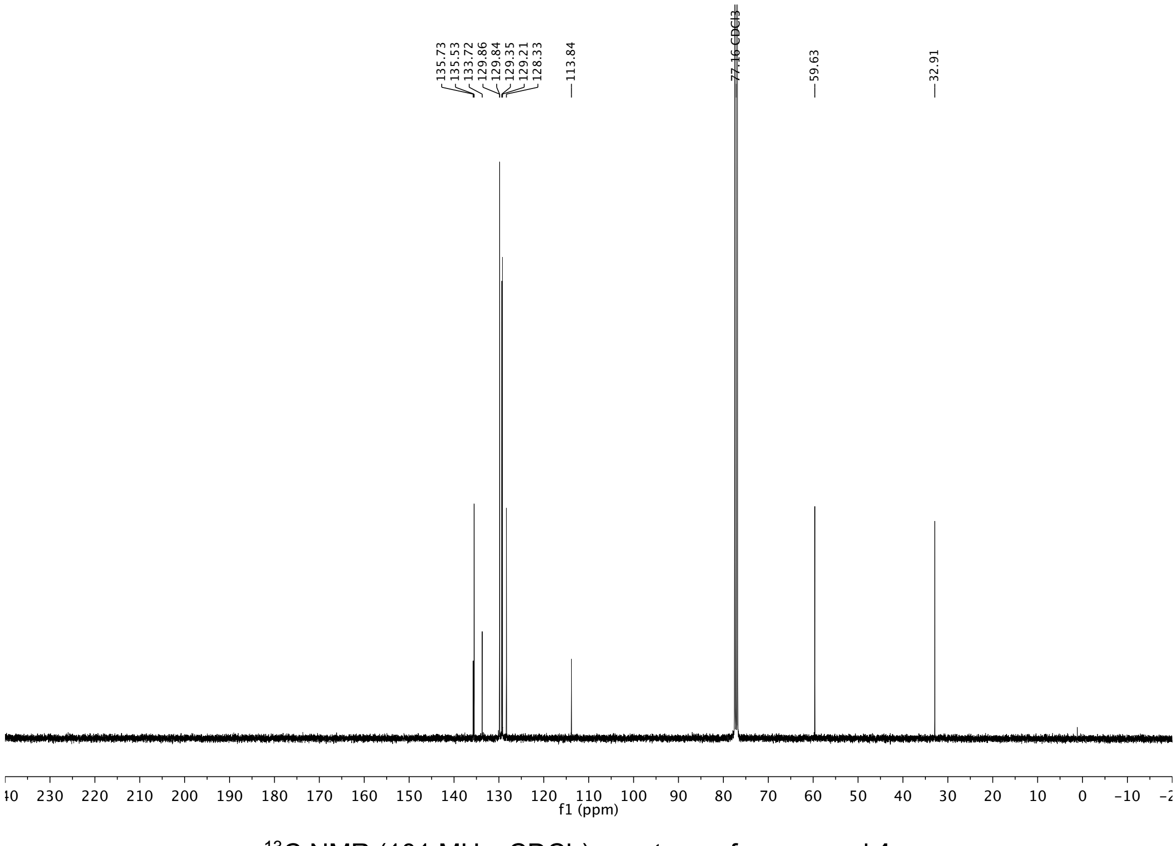

^{13}C NMR (101 MHz, $CDCl_3$) spectrum of compound **1a**.

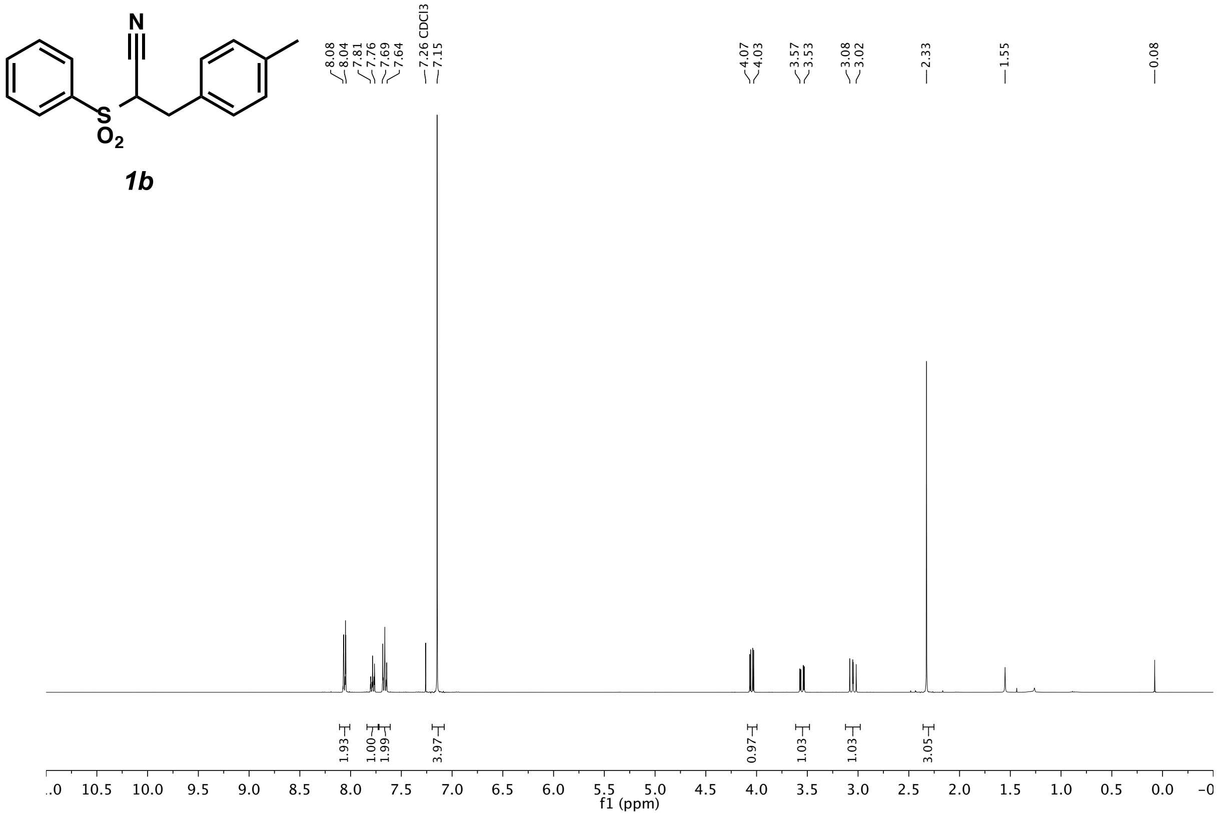

^{1}H NMR (400 MHz, CDCl$_3$) spectrum of compound **1b**.

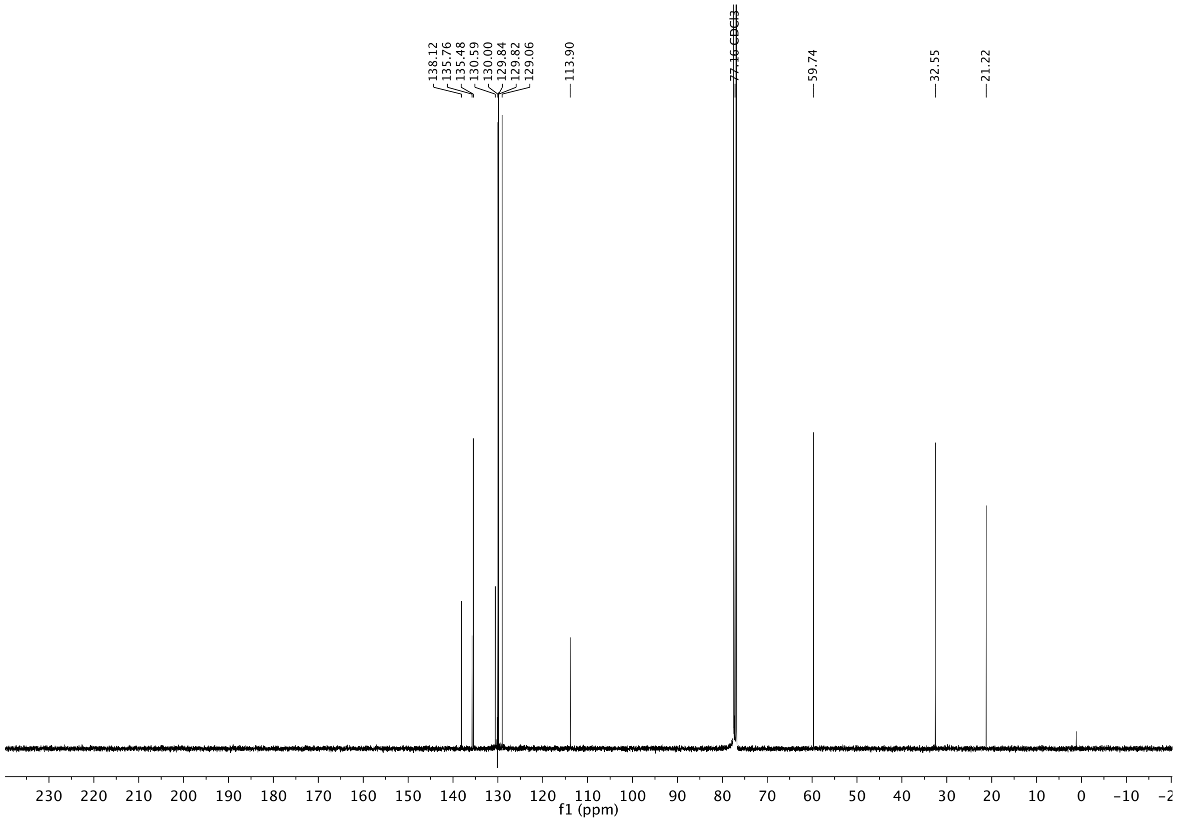

^{13}C NMR (101 MHz, CDCl$_3$) spectrum of compound **1b**.

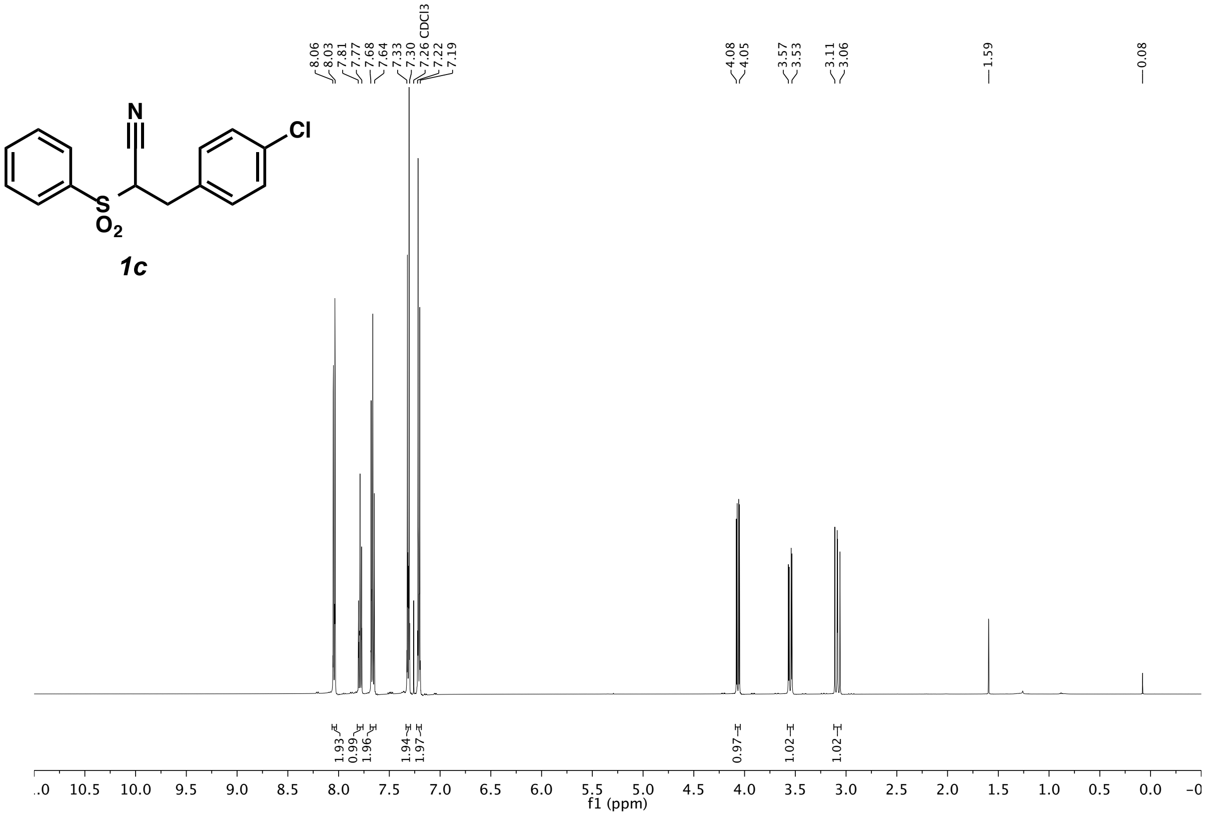

^{1}H NMR (400 MHz, CDCl3) spectrum of compound **1c**.

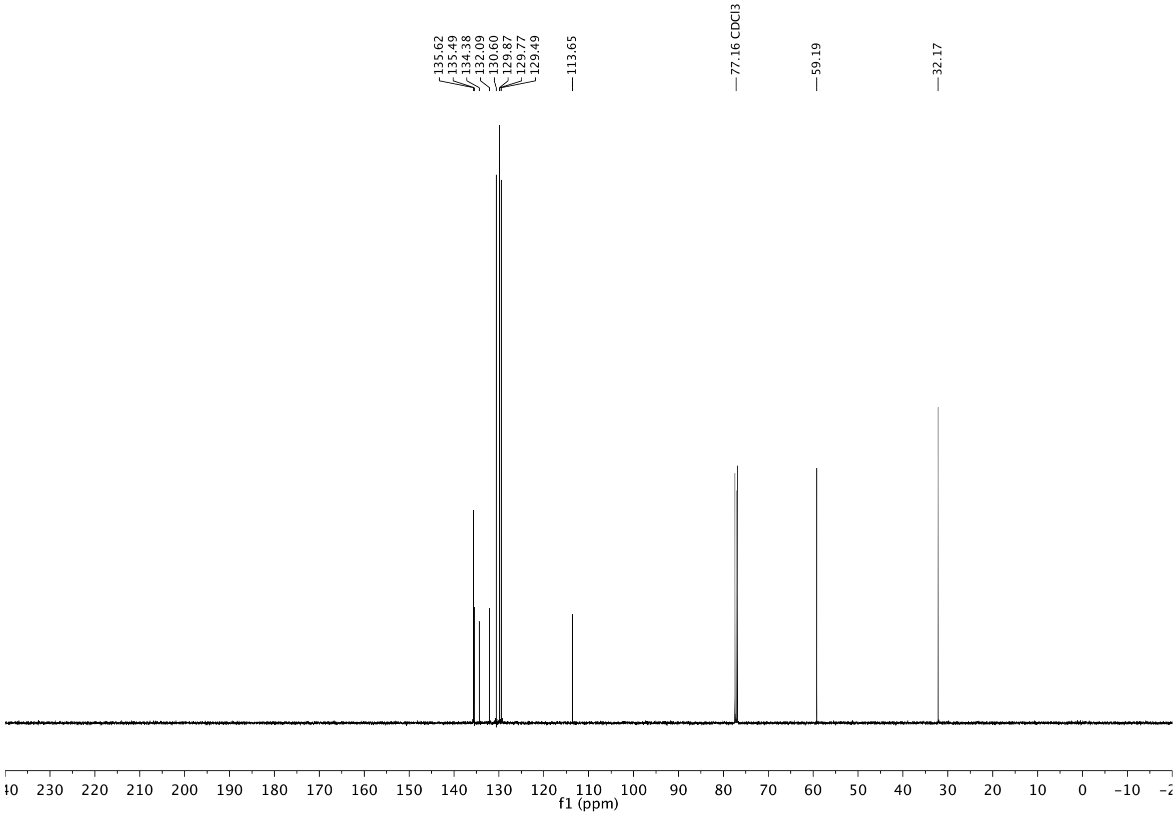

^{13}C NMR (101 MHz, CDCl3) spectrum of compound **1c**.

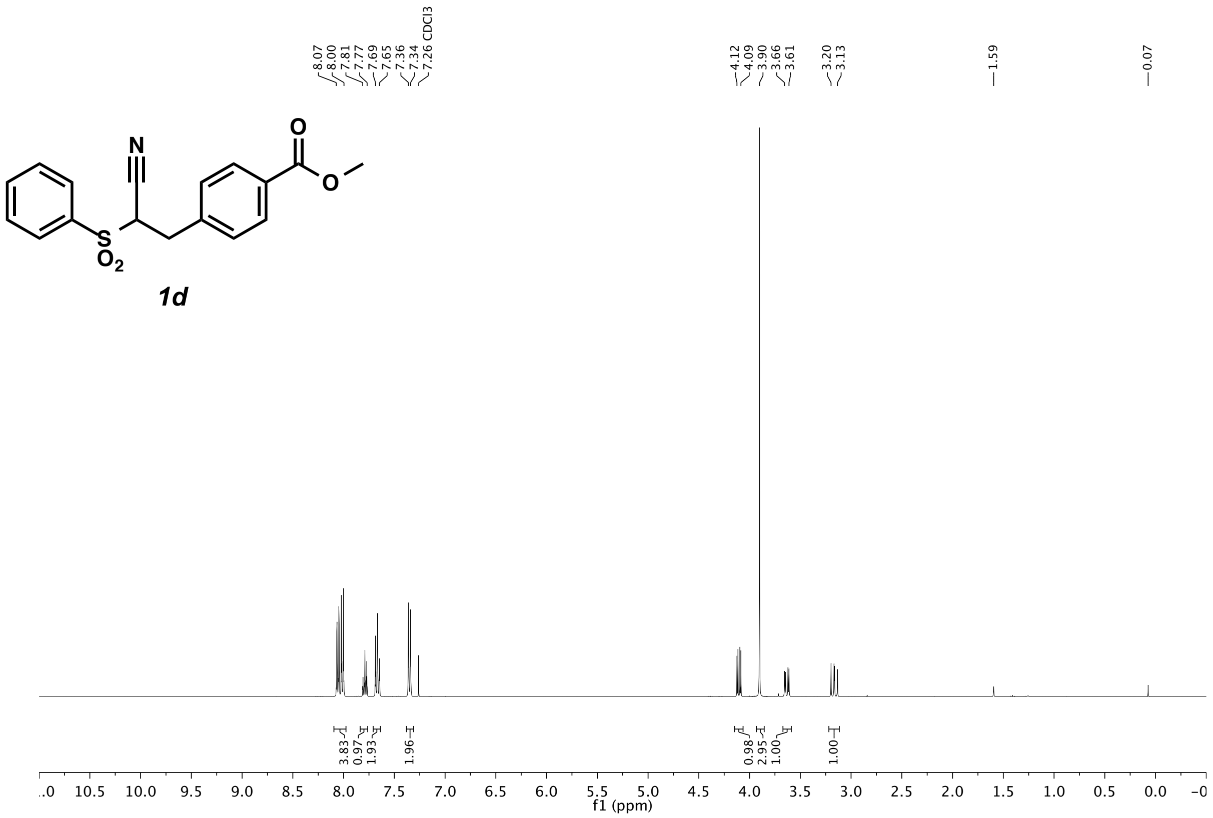

1d

^{1}H NMR (400 MHz, $CDCl_3$) spectrum of compound **1d**.

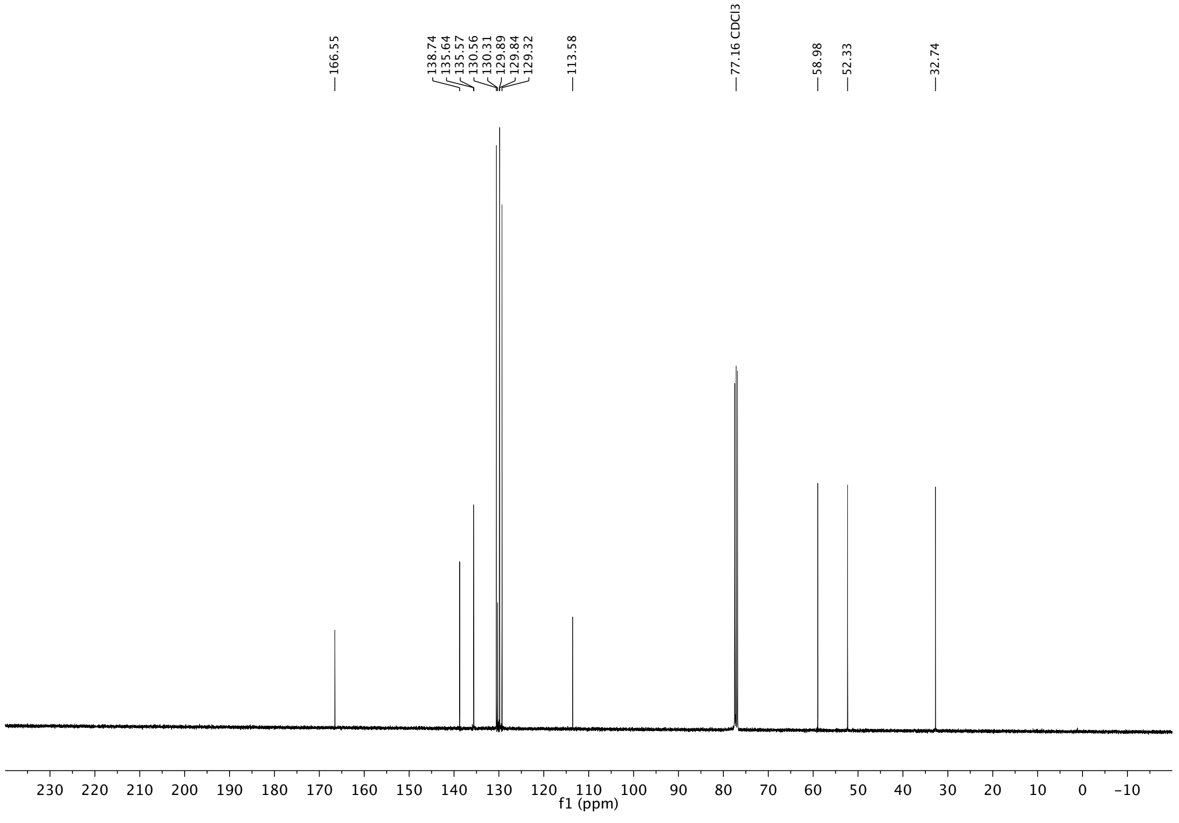

^{13}C NMR (101 MHz, $CDCl_3$) spectrum of compound **1d**.

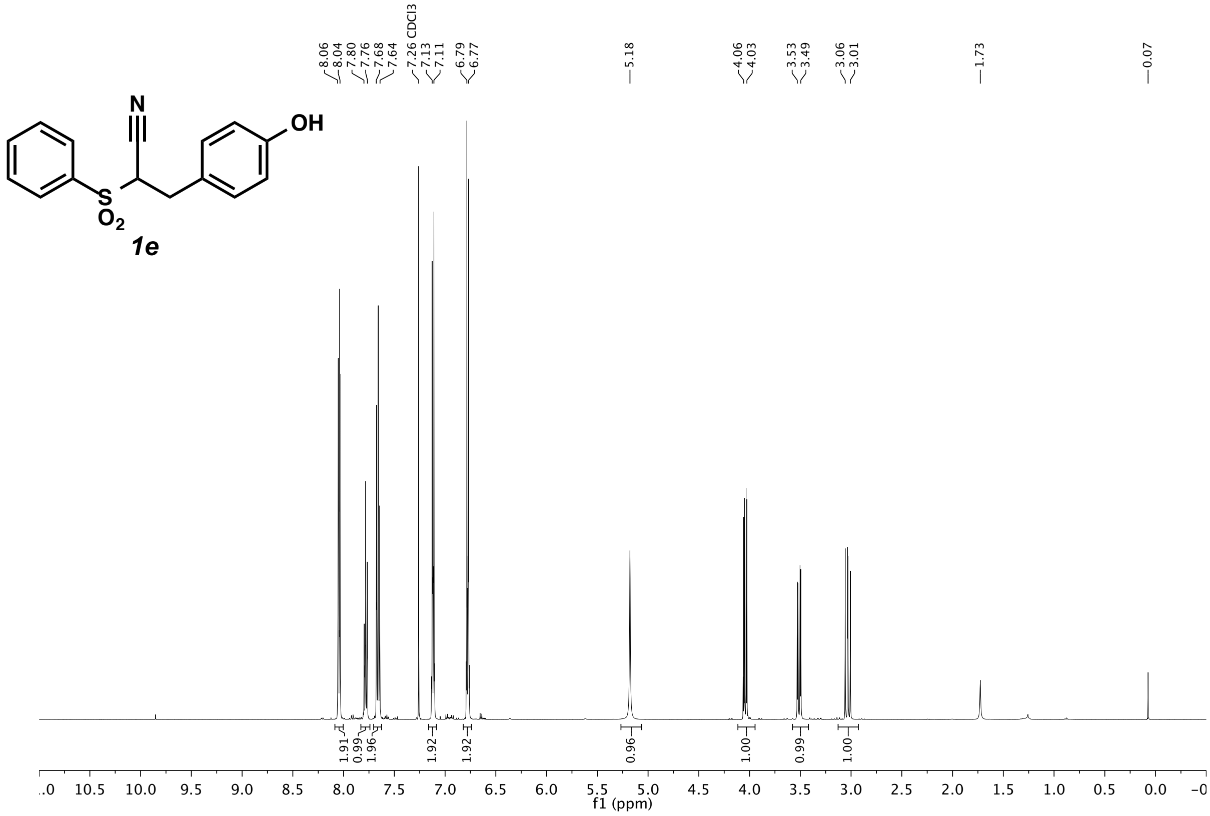

^{1}H NMR (400 MHz, $CDCl_3$) spectrum of compound **1e**.

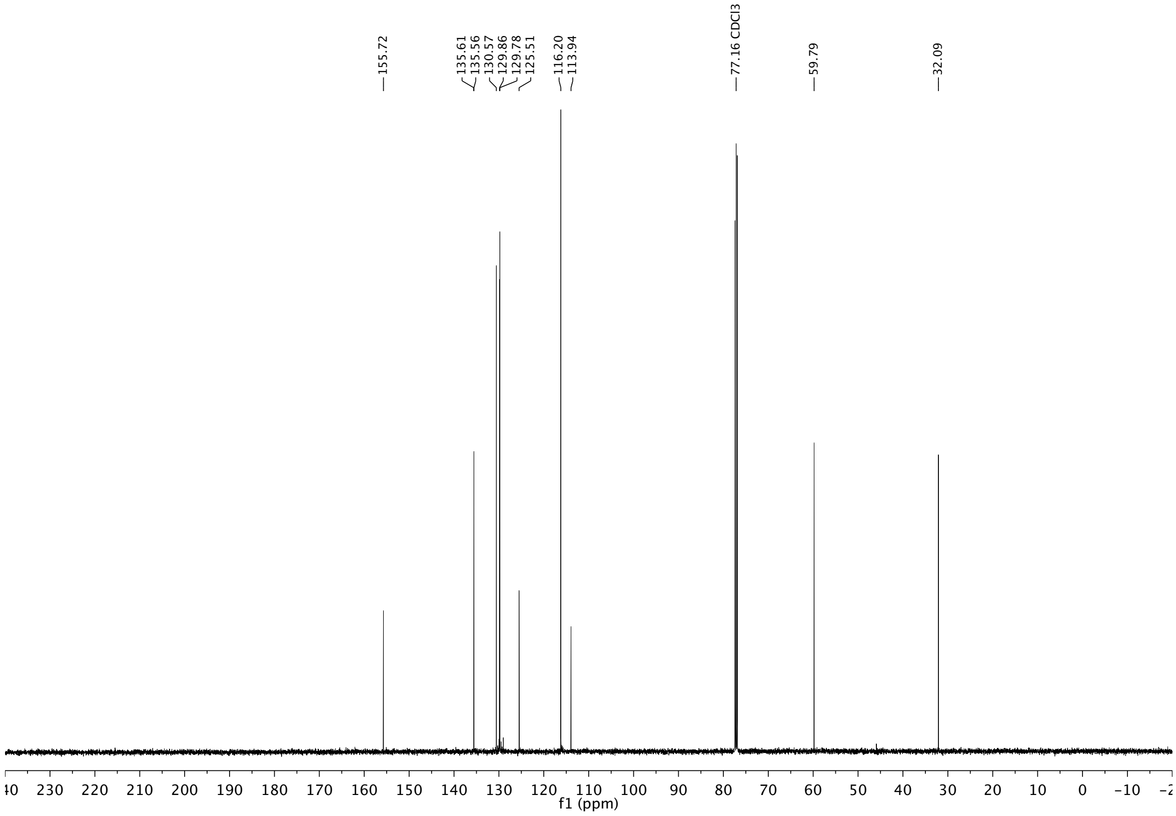

^{13}C NMR (101 MHz, $CDCl_3$) spectrum of compound **1e**.

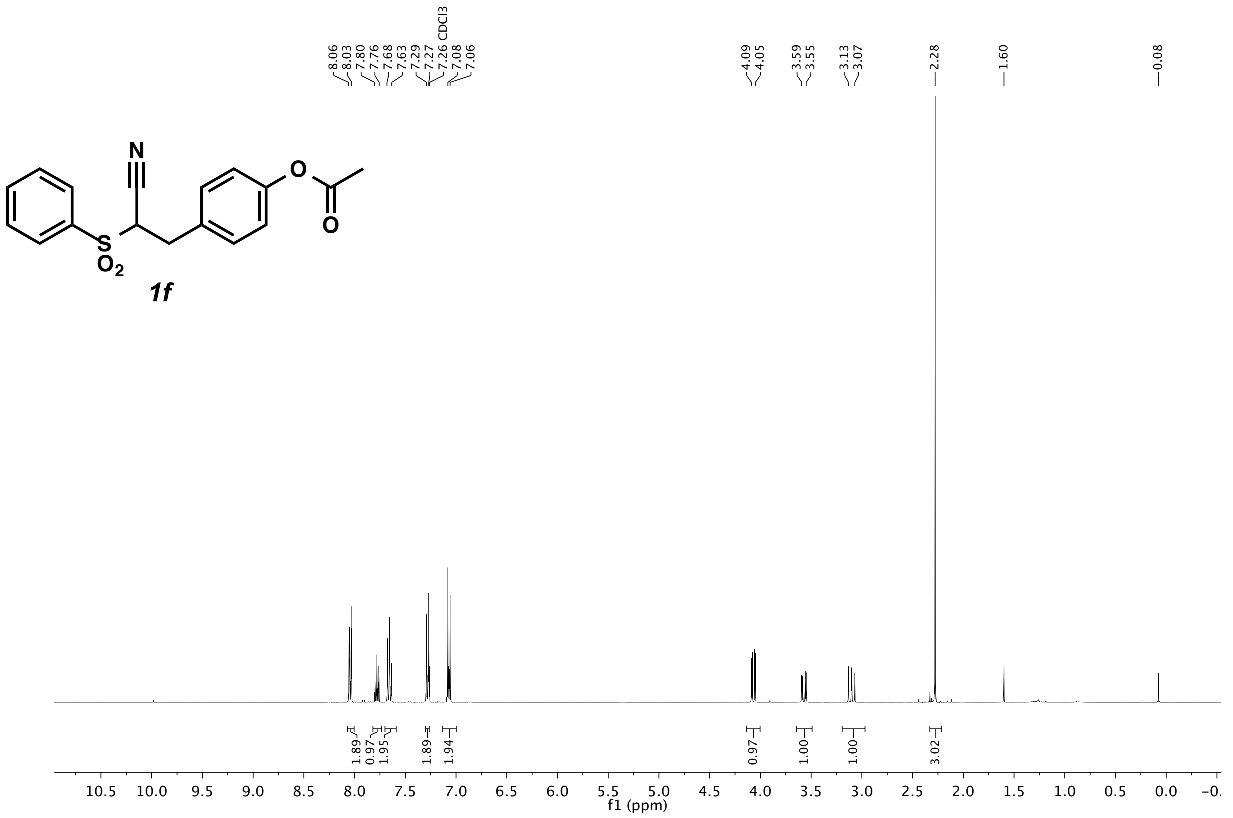

1f

^{1}H NMR (400 MHz, $CDCl_3$) spectrum of compound **1f**.

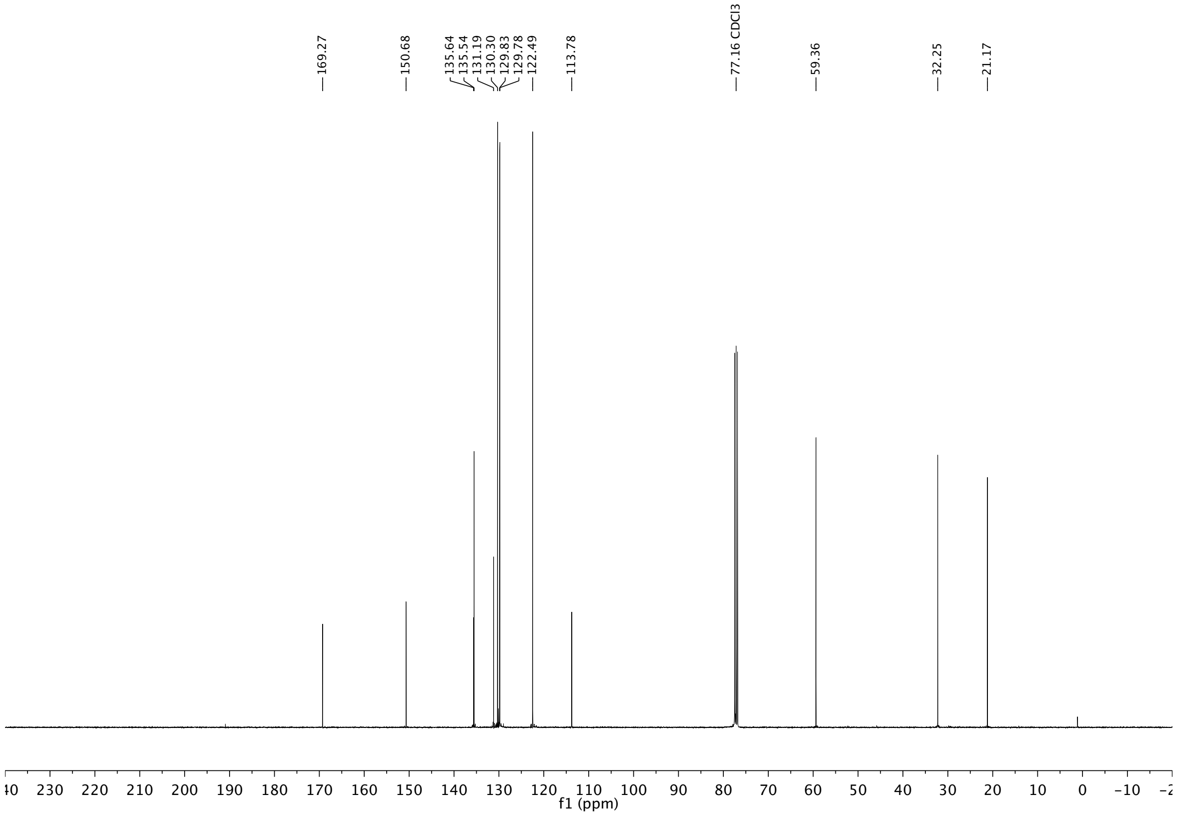

^{13}C NMR (101 MHz, $CDCl_3$) spectrum of compound **1f.**

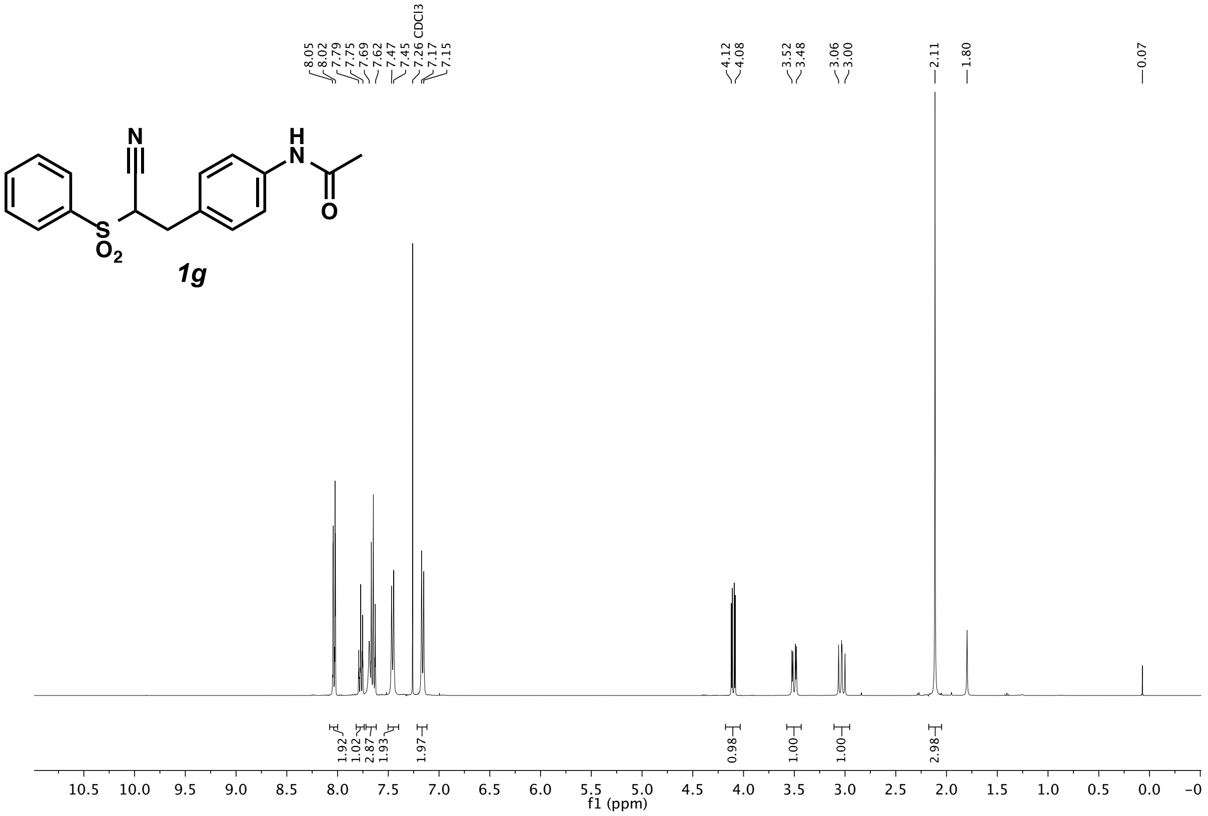

^{1}H NMR (400 MHz, $CDCl_3$) spectrum of compound **1g**.

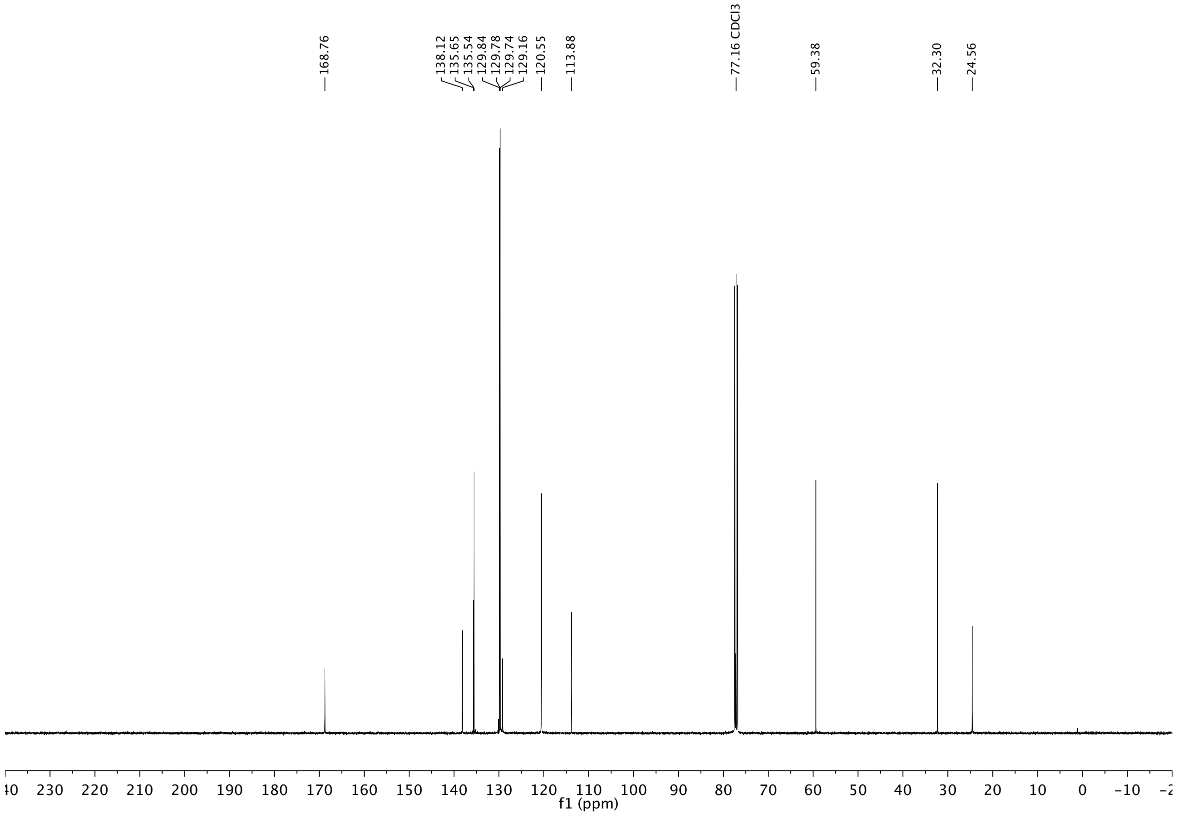

^{13}C NMR (101 MHz, $CDCl_3$) spectrum of compound **1g**.

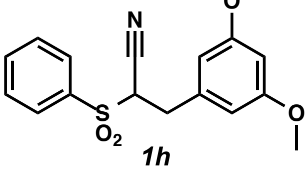

1h

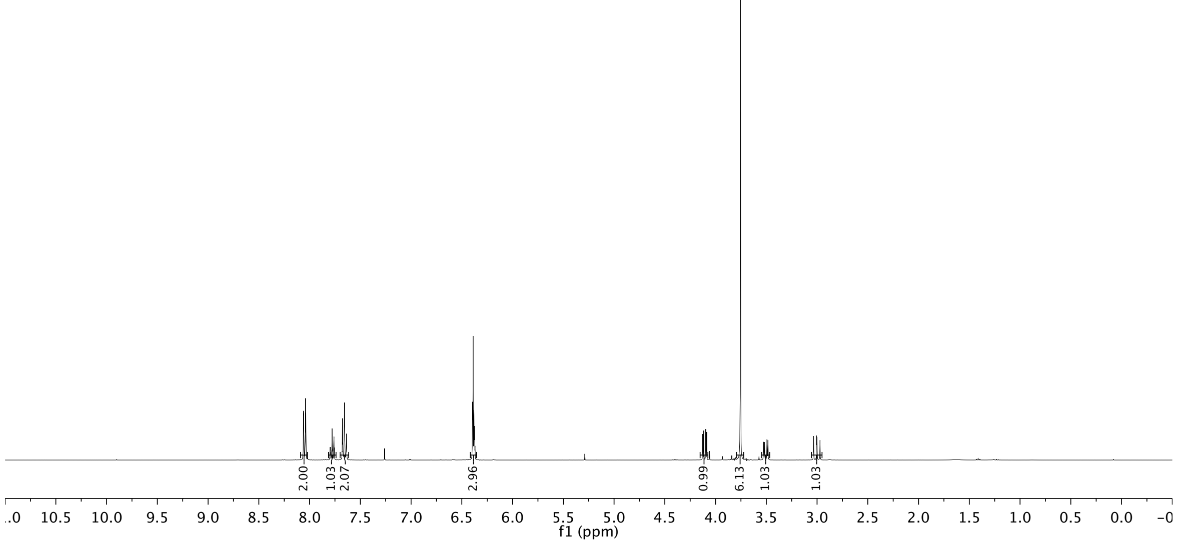

^{1}H NMR (400 MHz, $CDCl_3$) spectrum of compound **1h**.

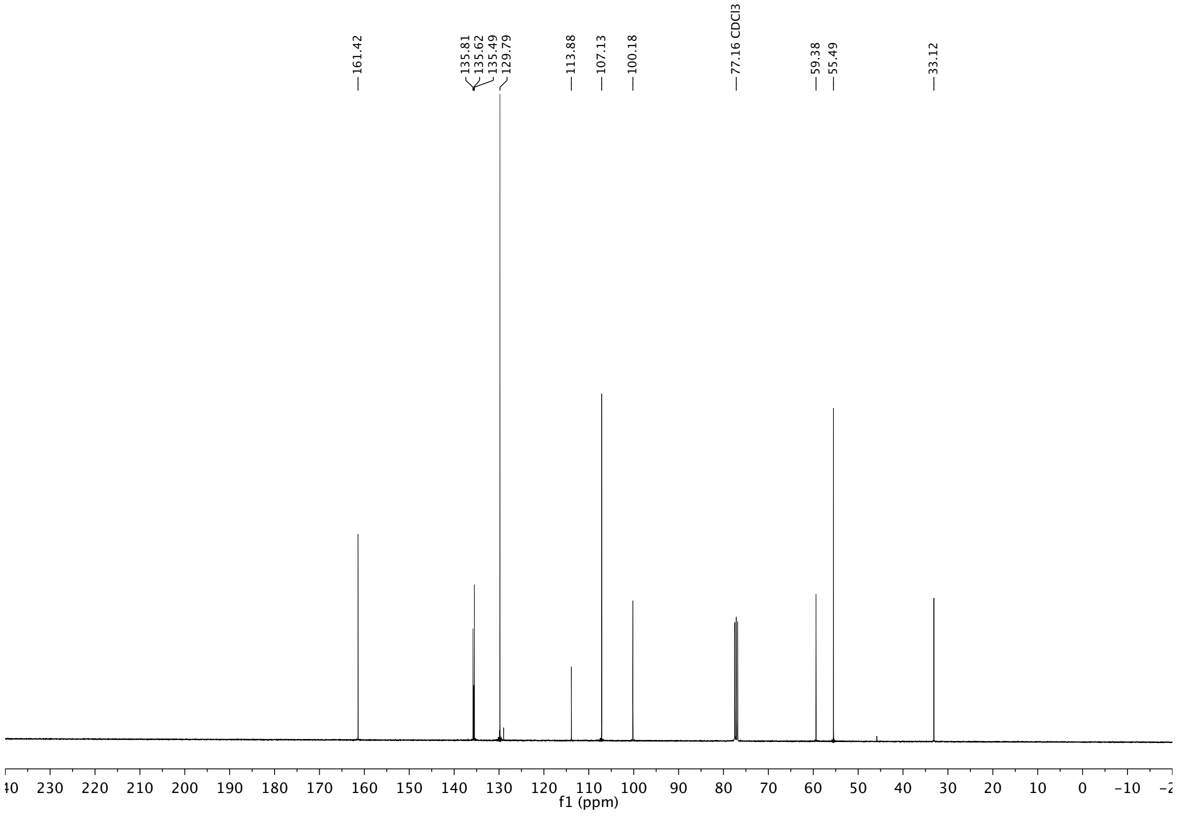

^{13}C NMR (101 MHz, $CDCl_3$) spectrum of compound **1h**.

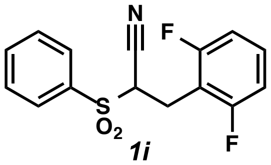

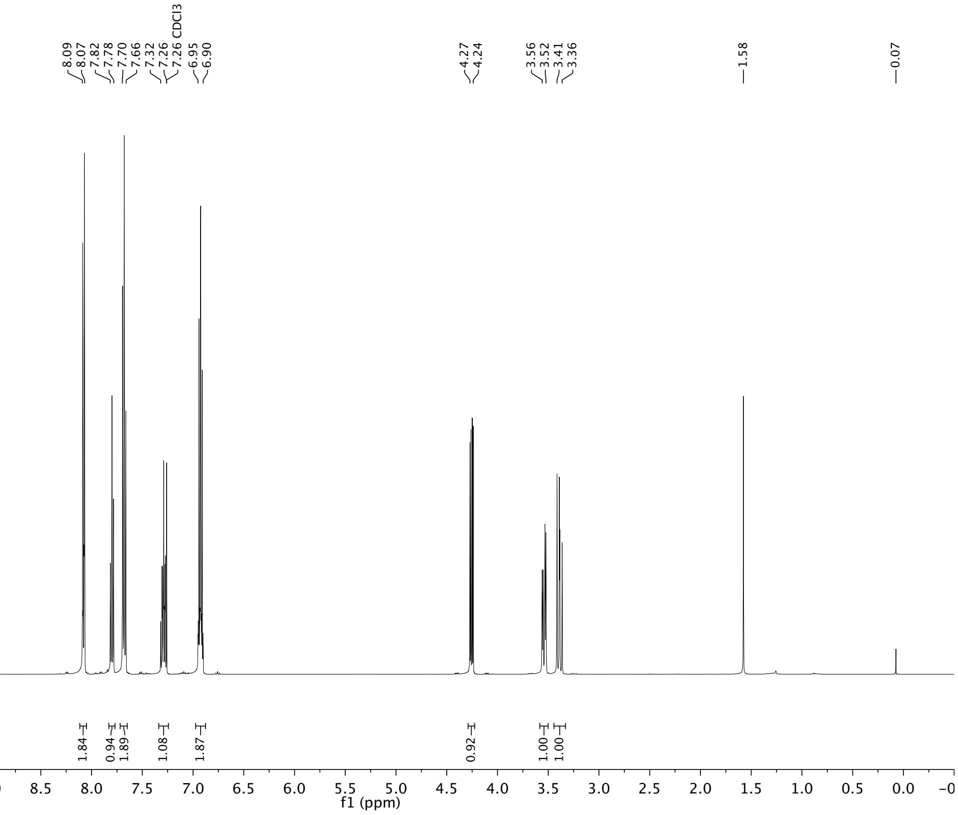

^{1}H NMR (500 MHz, $CDCl_3$) spectrum of compound **1i**.

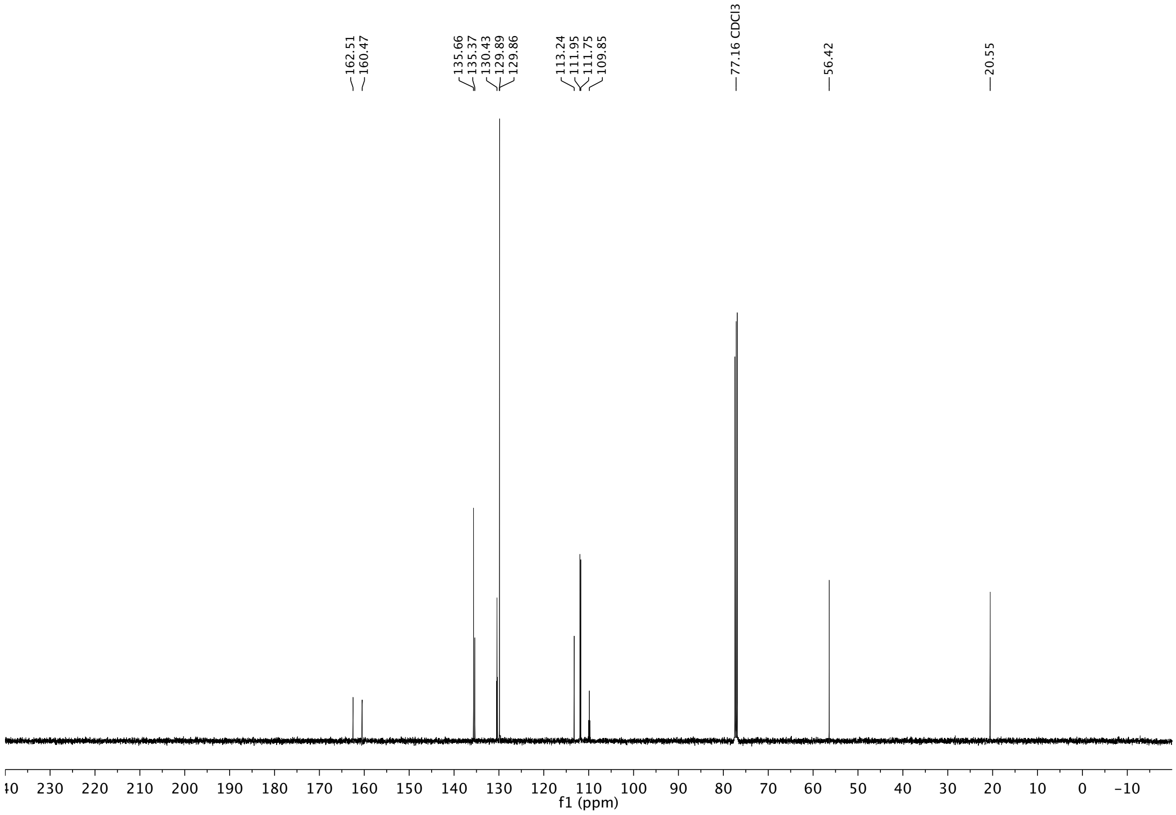

^{13}C NMR (126 MHz, $CDCl_3$) spectrum of compound **1i**.

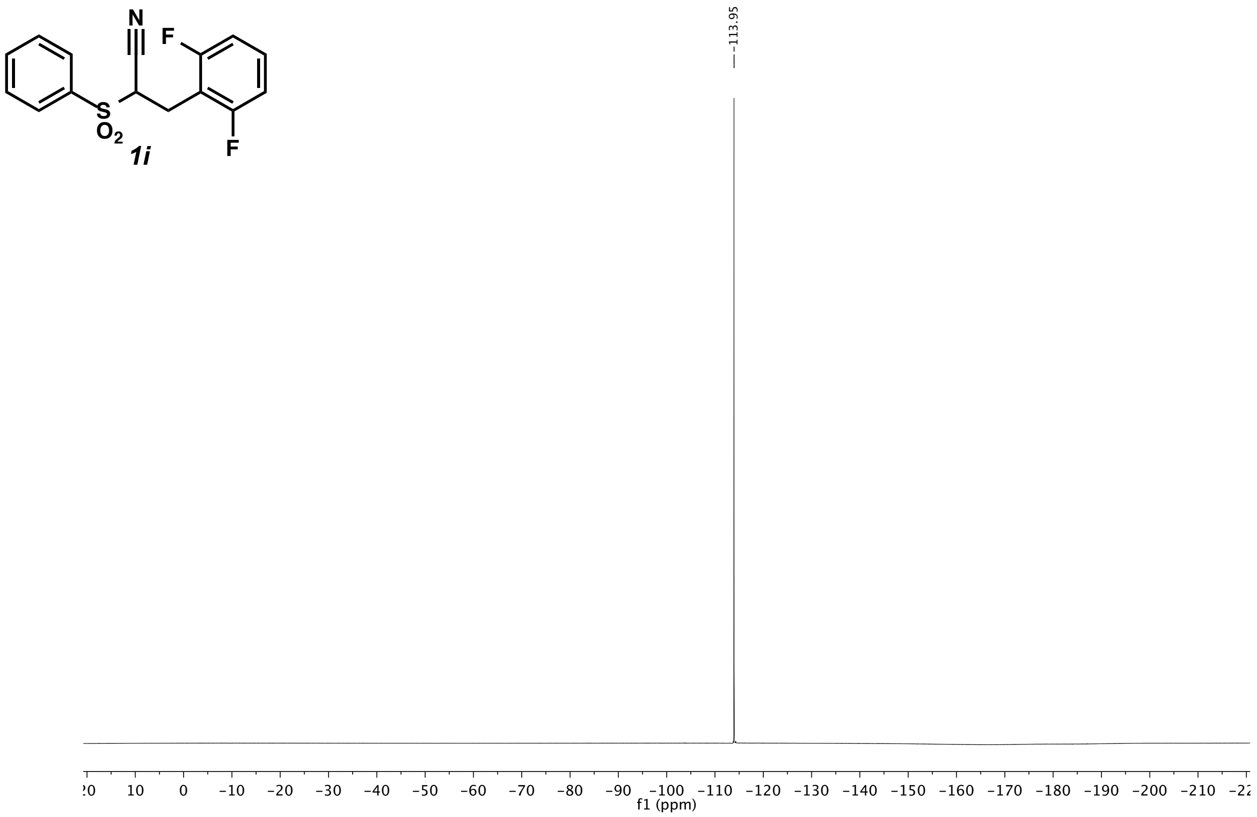

^{19}F NMR (470 MHz, $CDCl_3$) spectrum of compound **1i**.

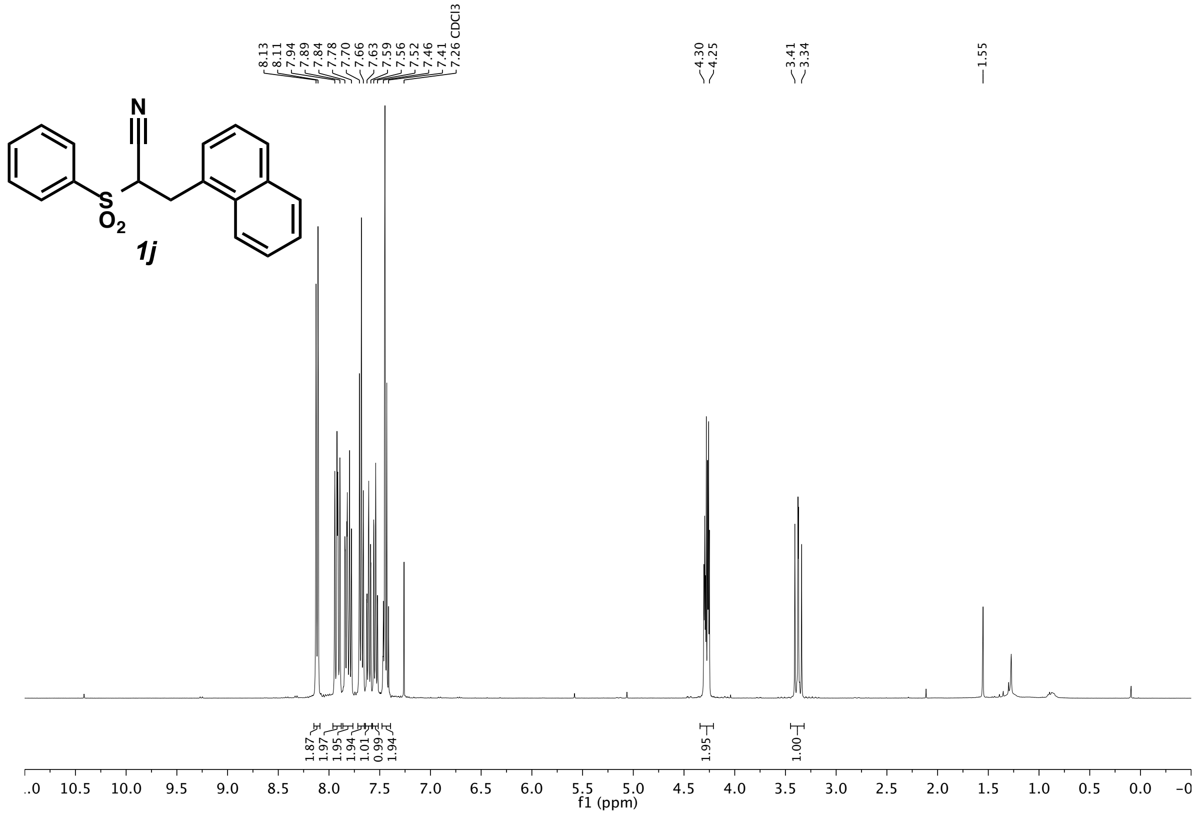

^{1}H NMR (400 MHz, $CDCl_3$) spectrum of compound **1j**.

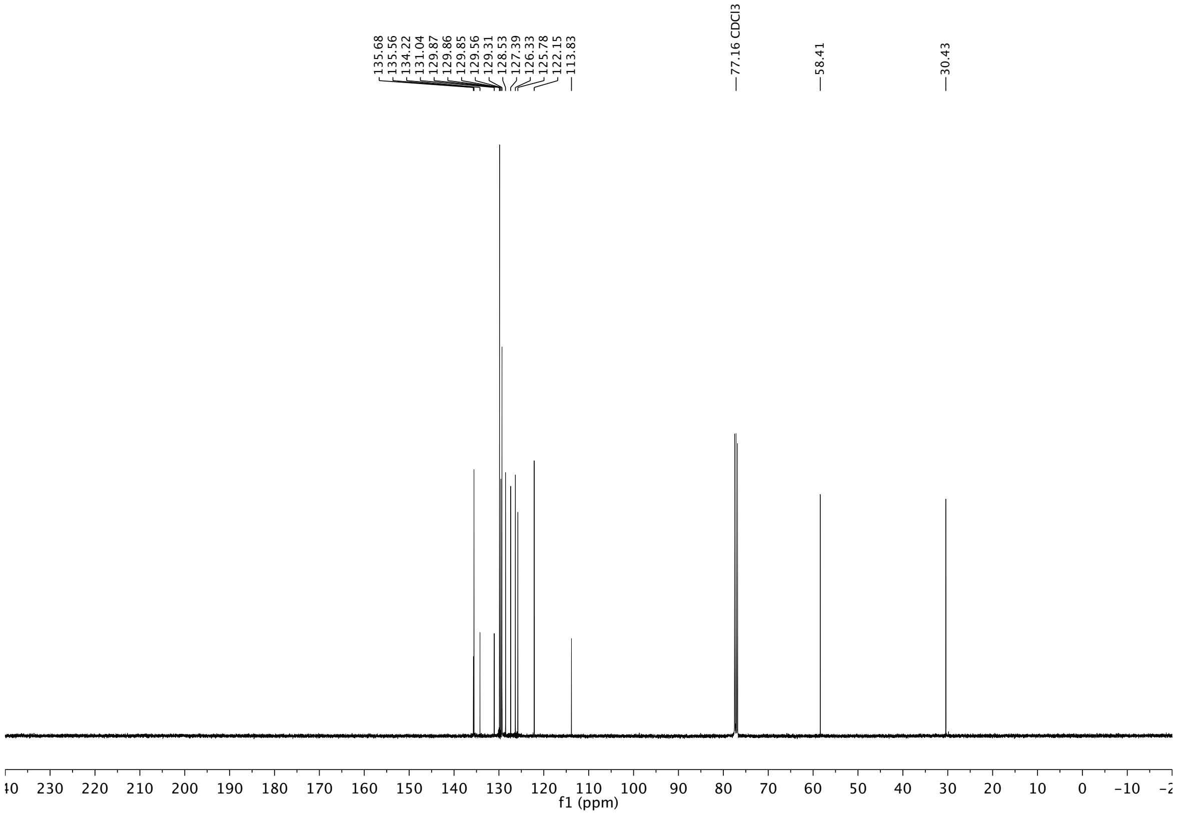

^{13}C NMR (101 MHz, $CDCl_3$) spectrum of compound **1j**.

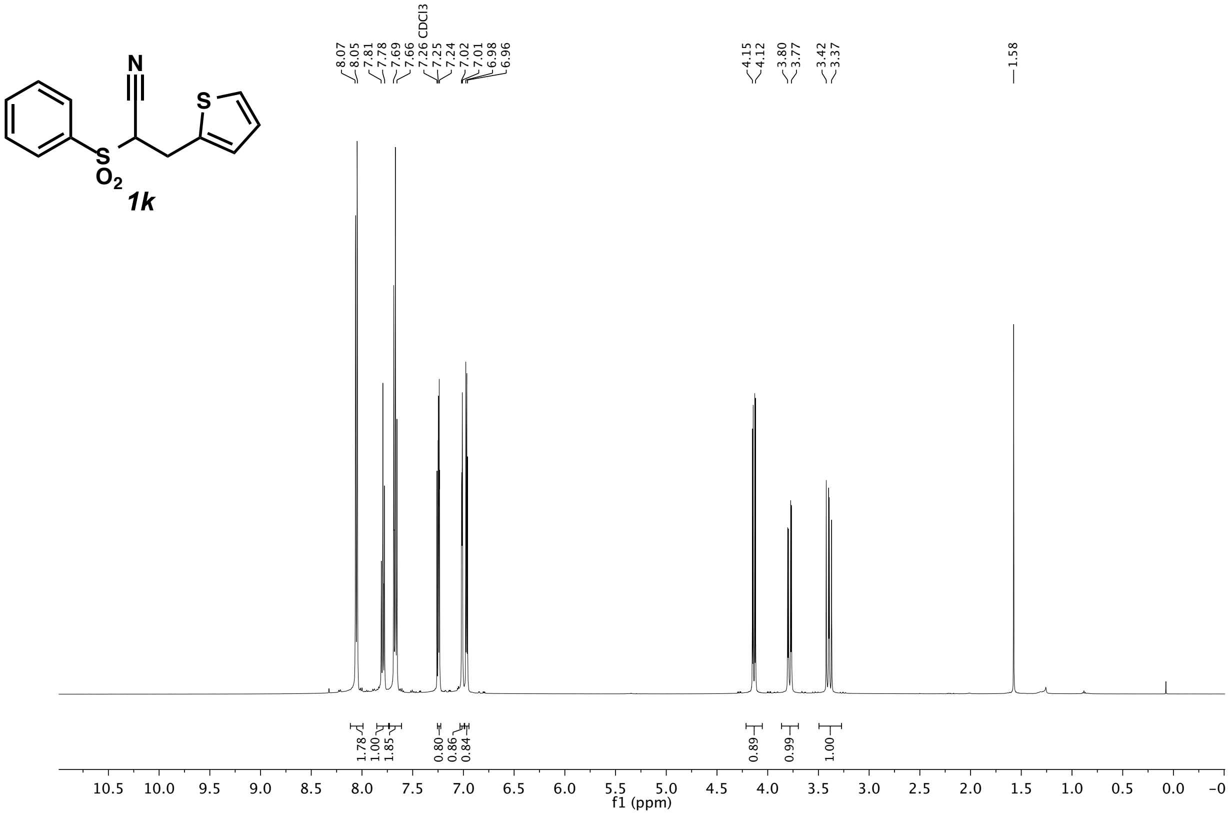

^{1}H NMR (500 MHz, CDCl3) spectrum of compound **1k**.

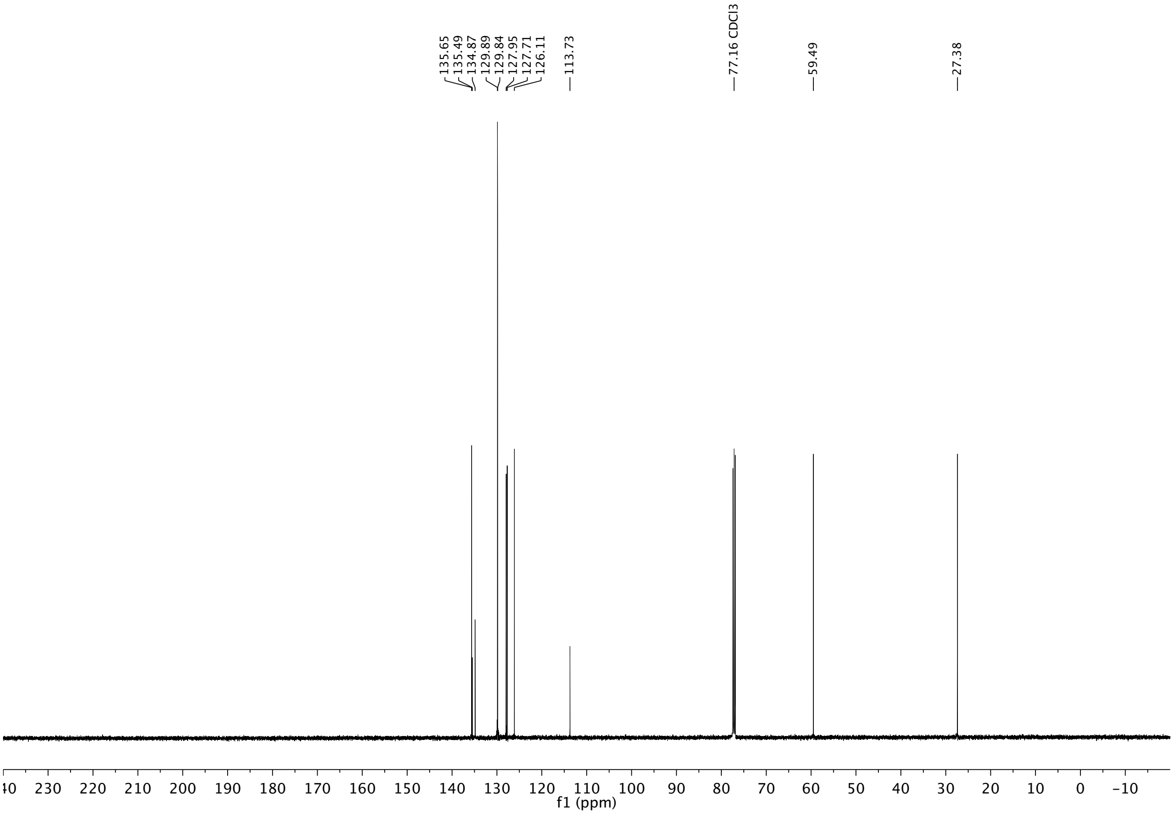

^{13}C NMR (126 MHz, CDCl3) spectrum of compound **1k**.

1l

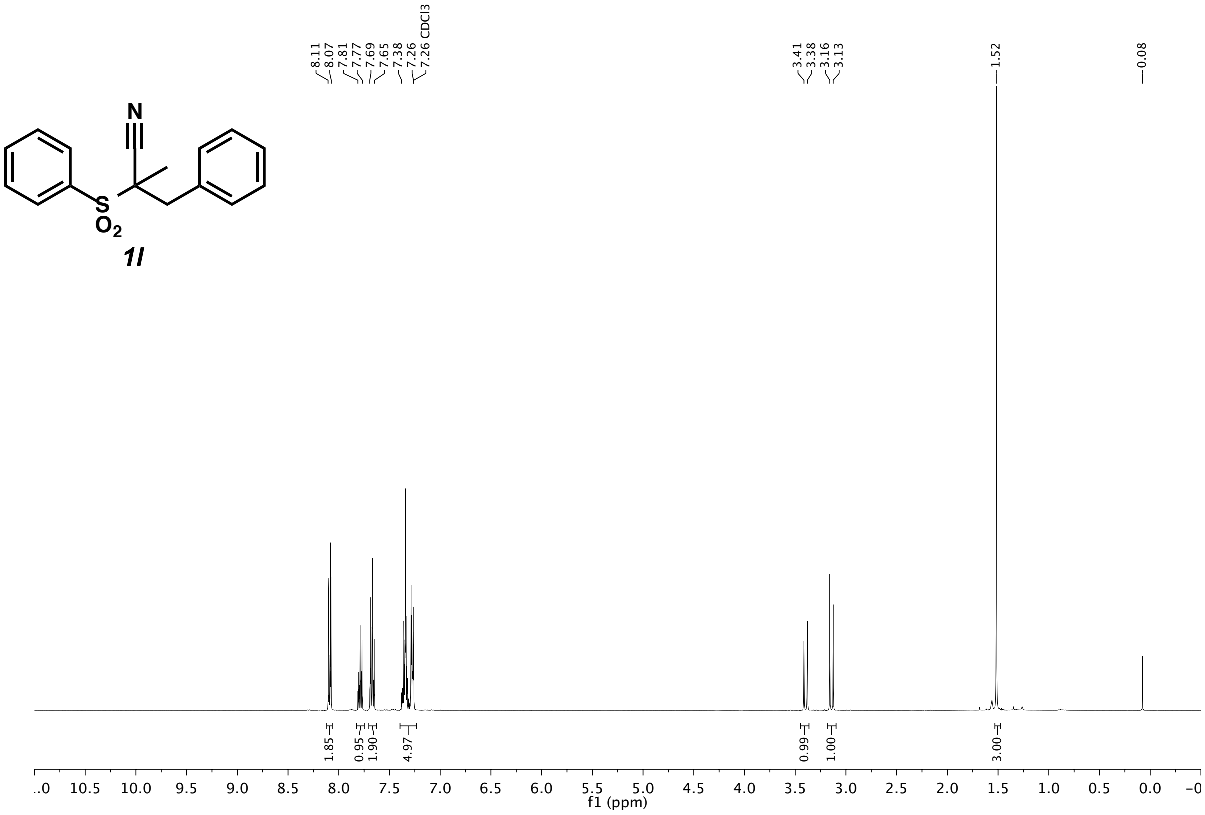

^{1}H NMR (400 MHz, $CDCl_3$) spectrum of compound **1l**.

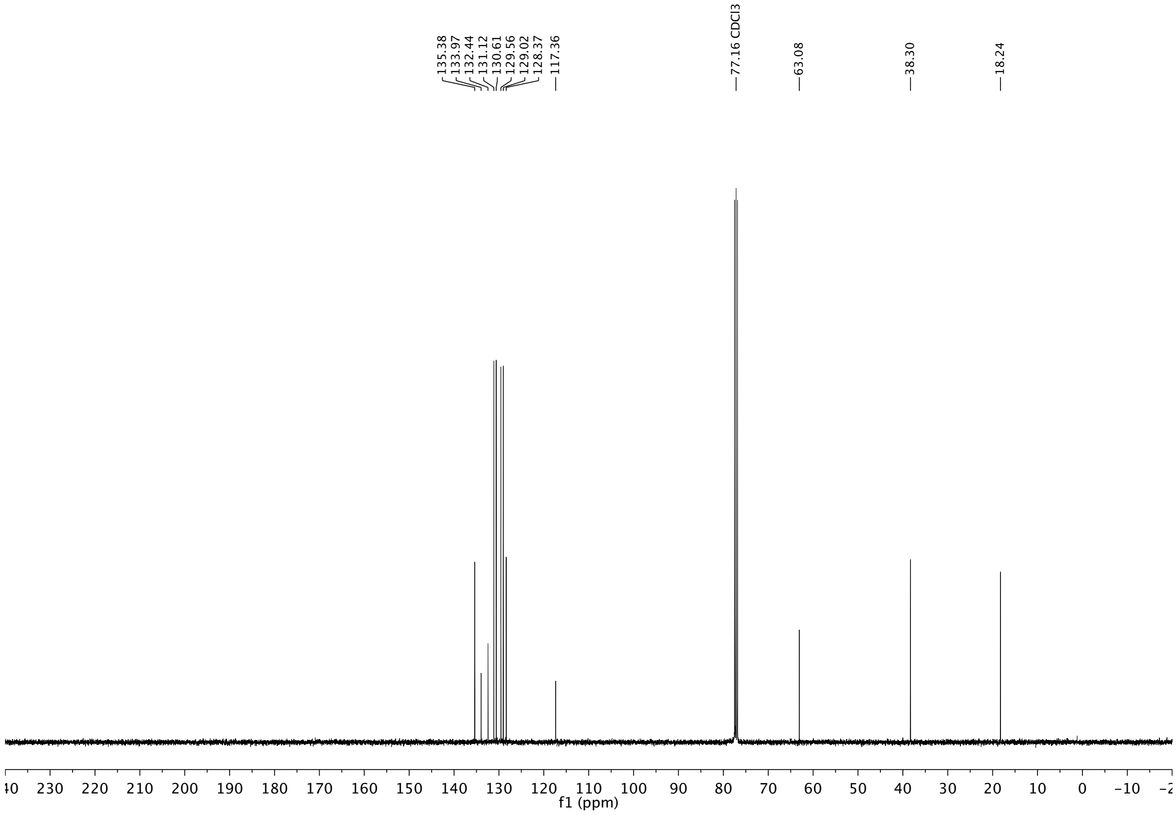

^{13}C NMR (101 MHz, $CDCl_3$) spectrum of compound **1l**.

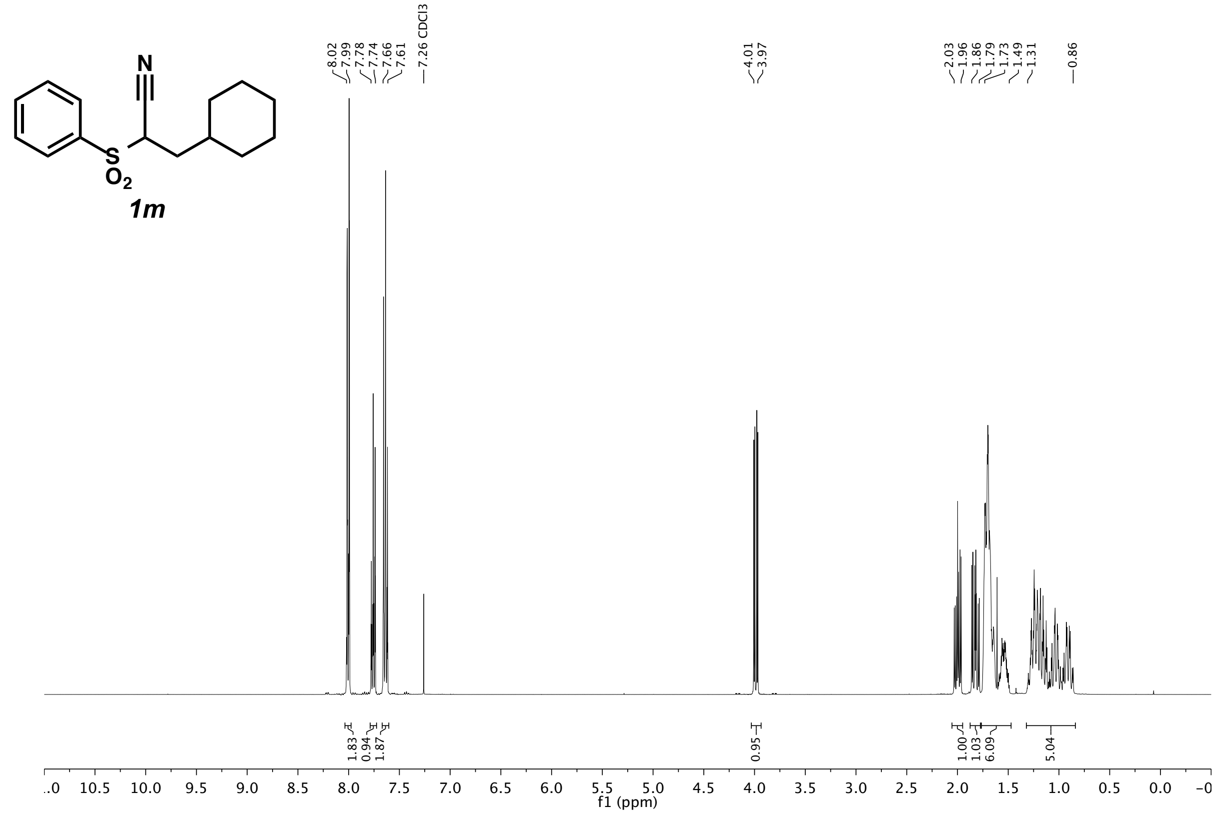

^{1}H NMR (400 MHz, $CDCl_3$) spectrum of compound **1m**.

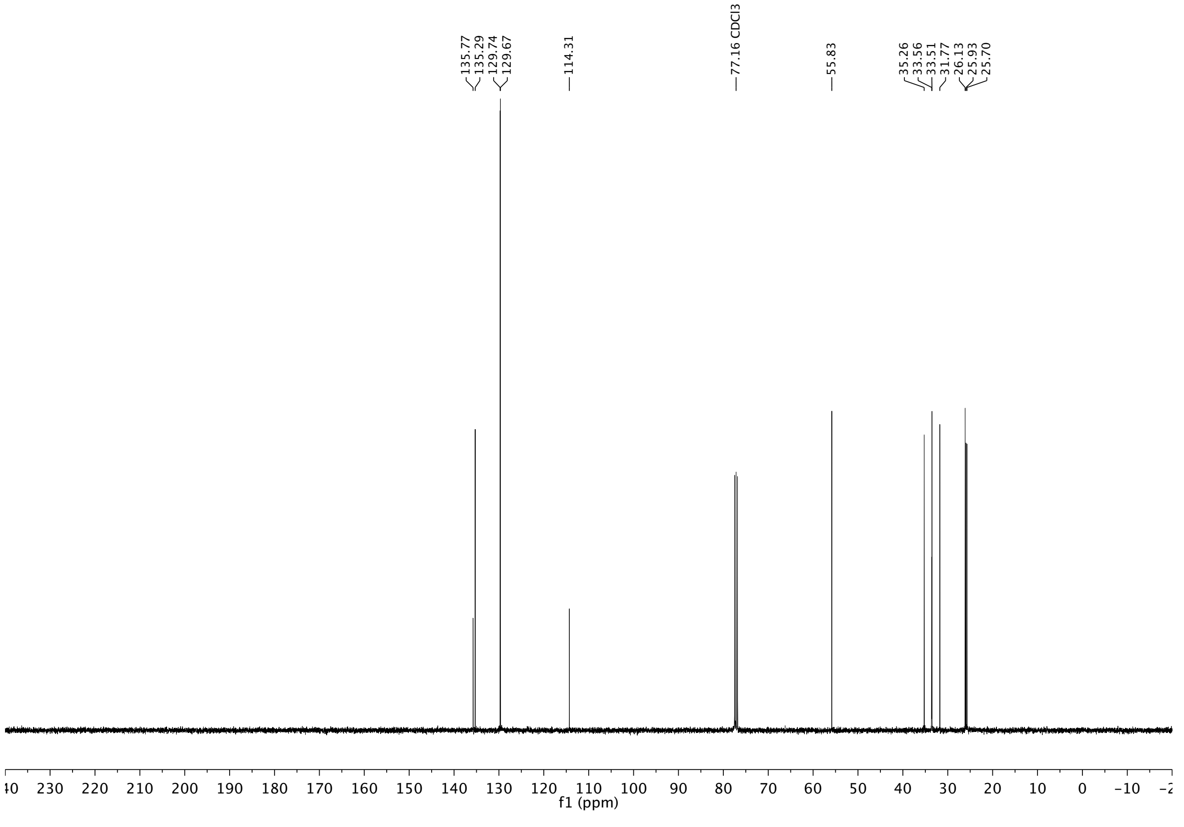

^{13}C NMR (101 MHz, $CDCl_3$) spectrum of compound **1m**.

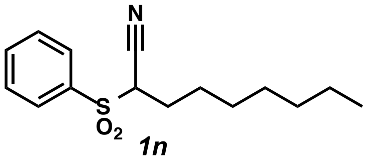

1n

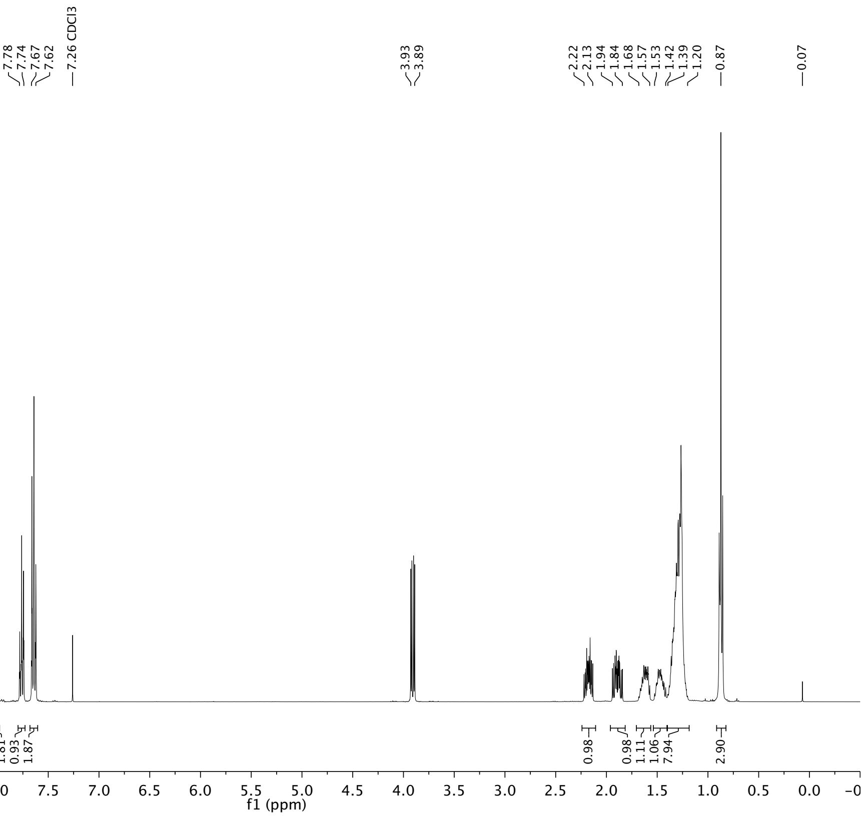

^{1}H NMR (400 MHz, $CDCl_3$) spectrum of compound **1n**.

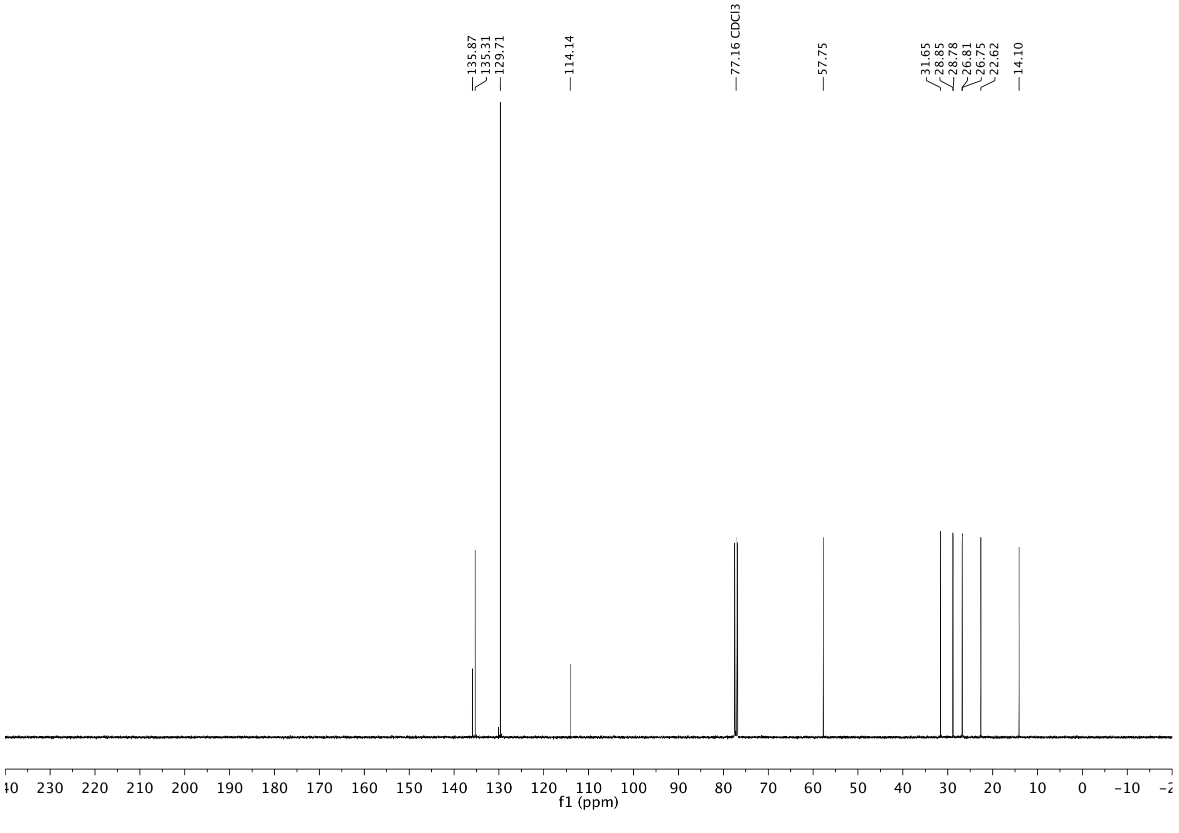

^{13}C NMR (101 MHz, $CDCl_3$) spectrum of compound **1n**.

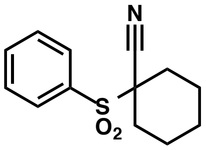

1o

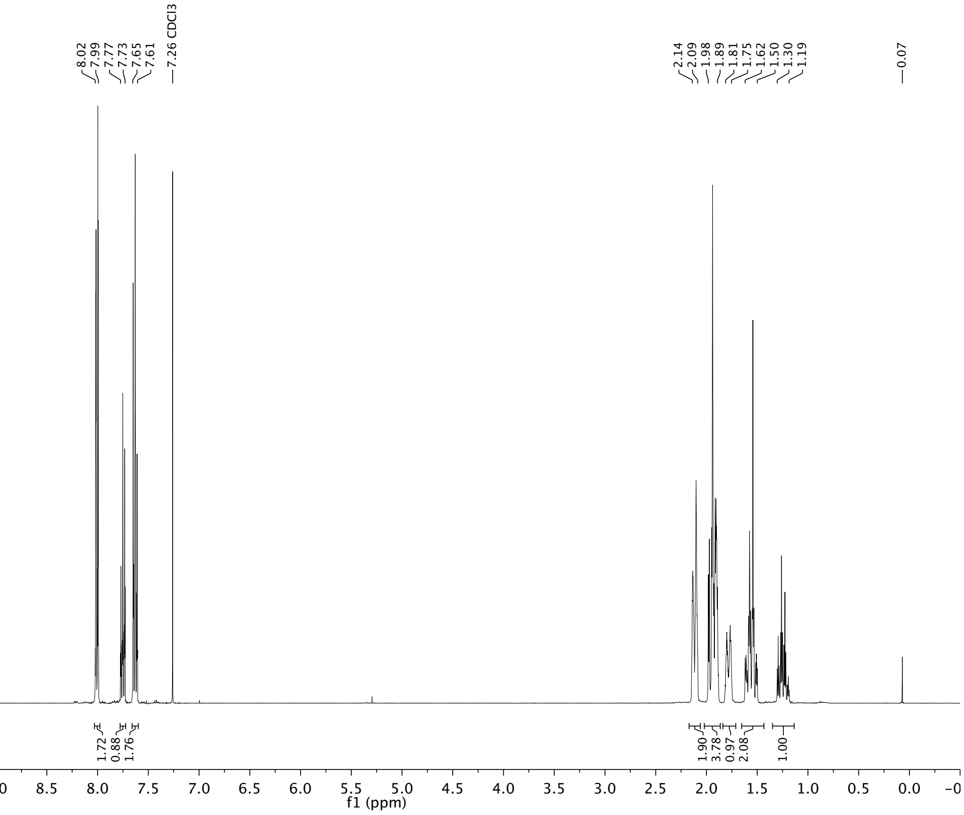

^{1}H NMR (400 MHz, $CDCl_3$) spectrum of compound **1o**.

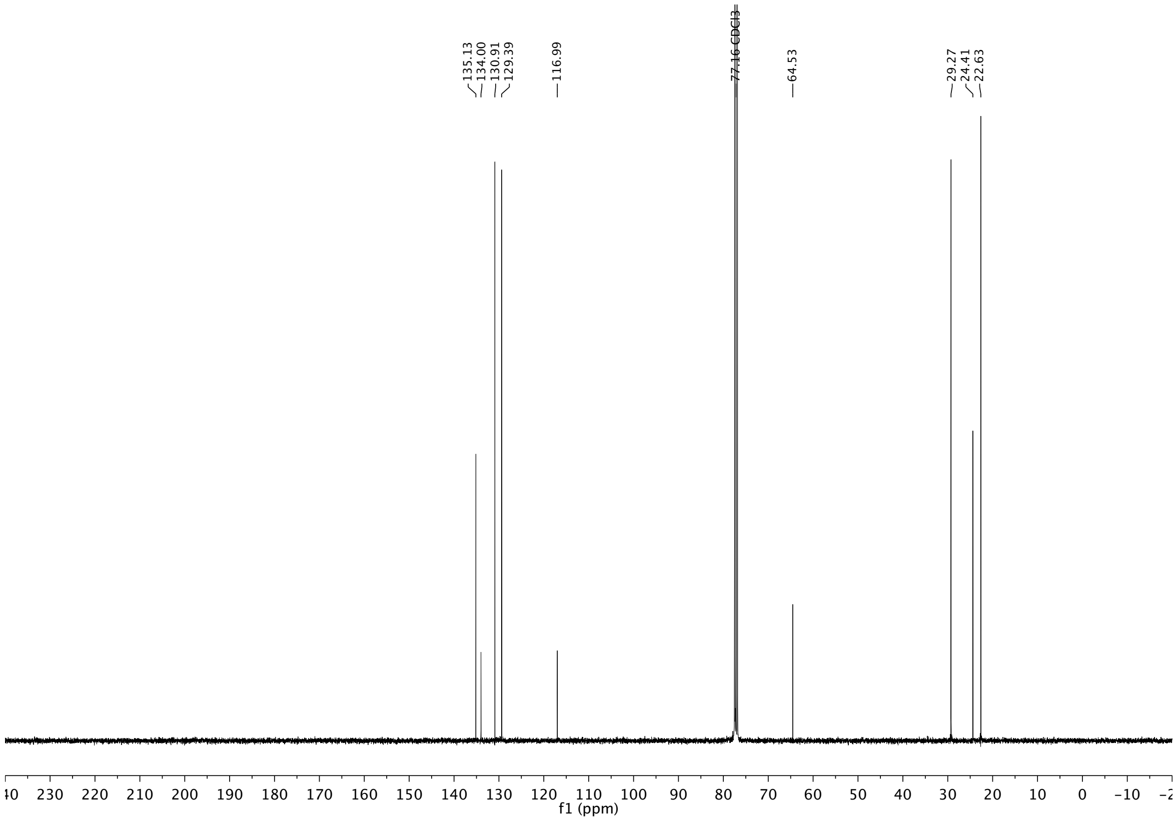

^{13}C NMR (101 MHz, $CDCl_3$) spectrum of compound **1o**.

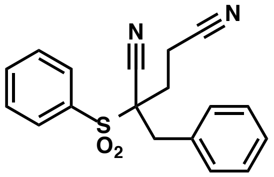

1p

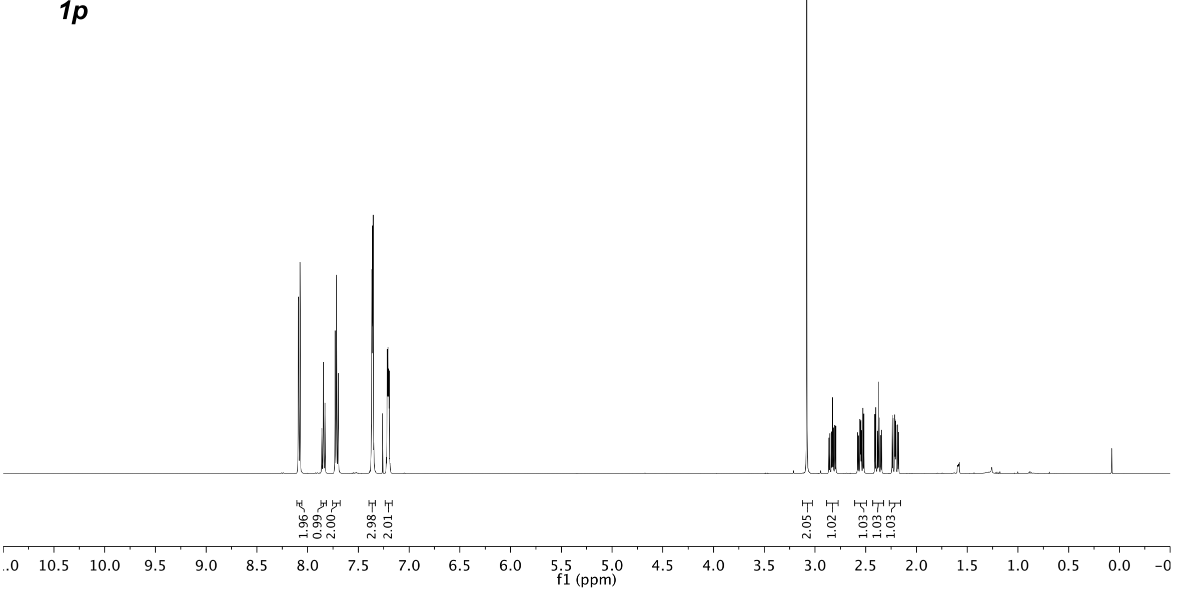

^{1}H NMR (500 MHz, CDCl3) spectrum of compound **1p**.

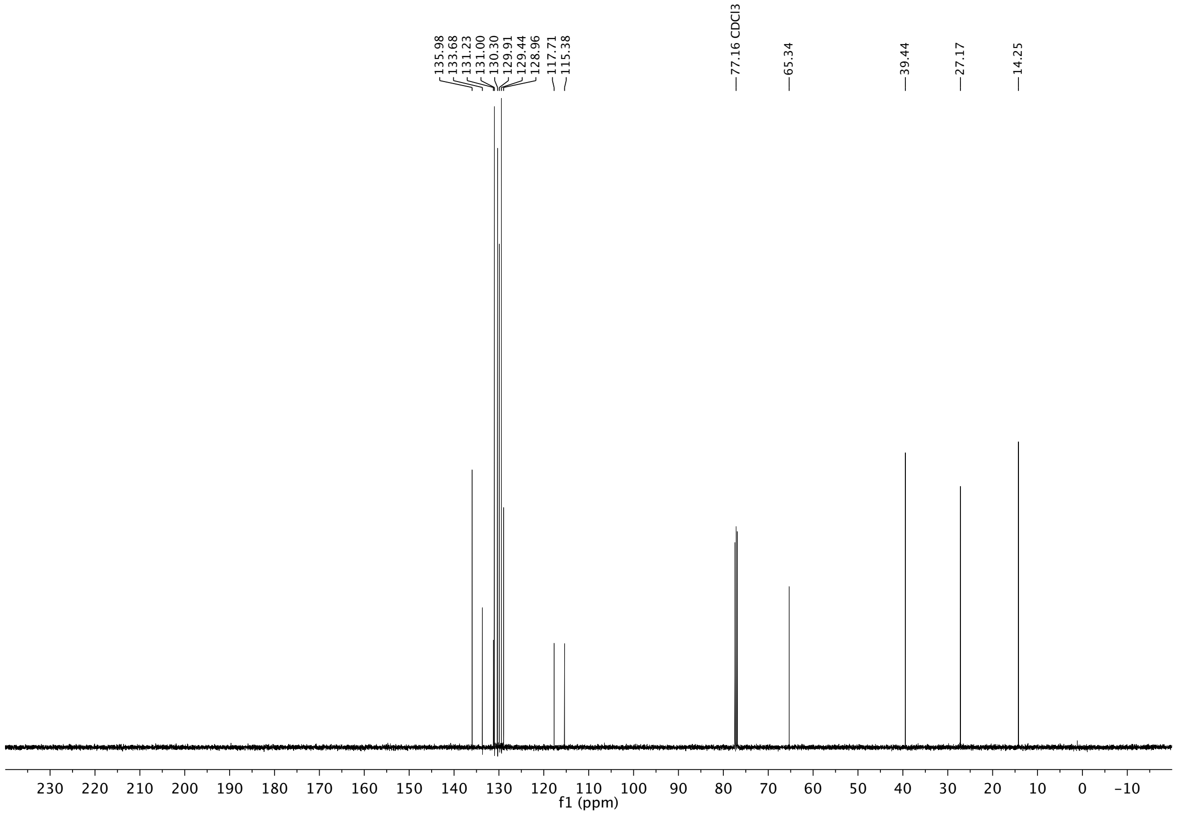

^{13}C NMR (126 MHz, CDCl3) spectrum of compound **1p**.

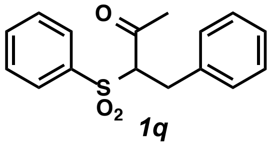

1q

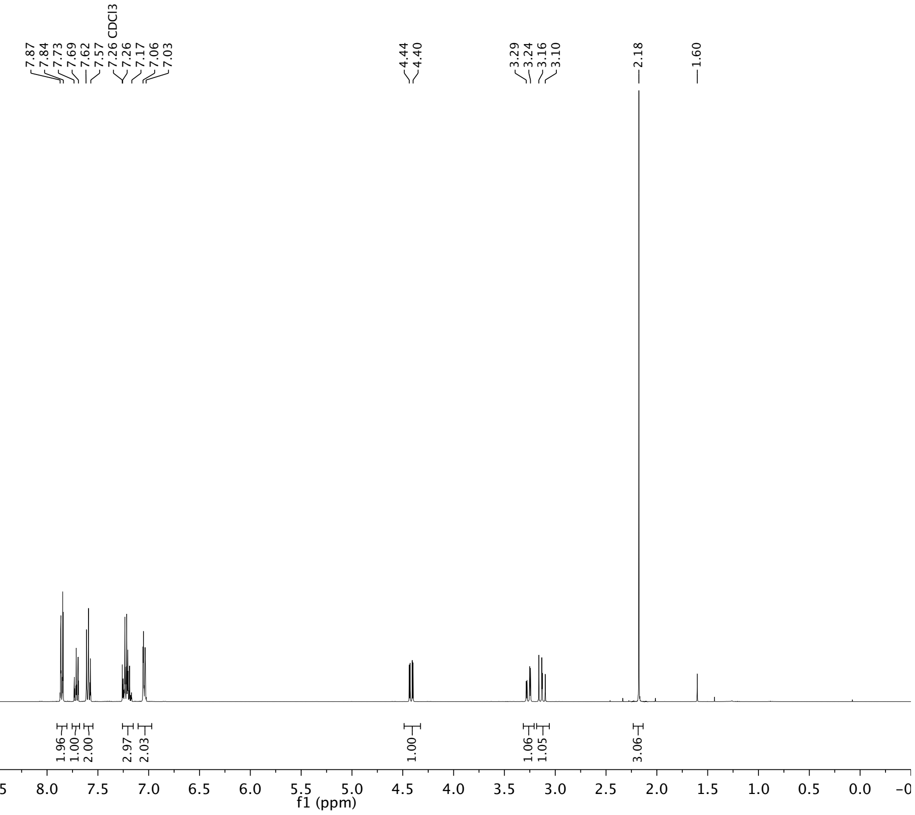

^{1}H NMR (400 MHz, $CDCl_3$) spectrum of compound **1q**.

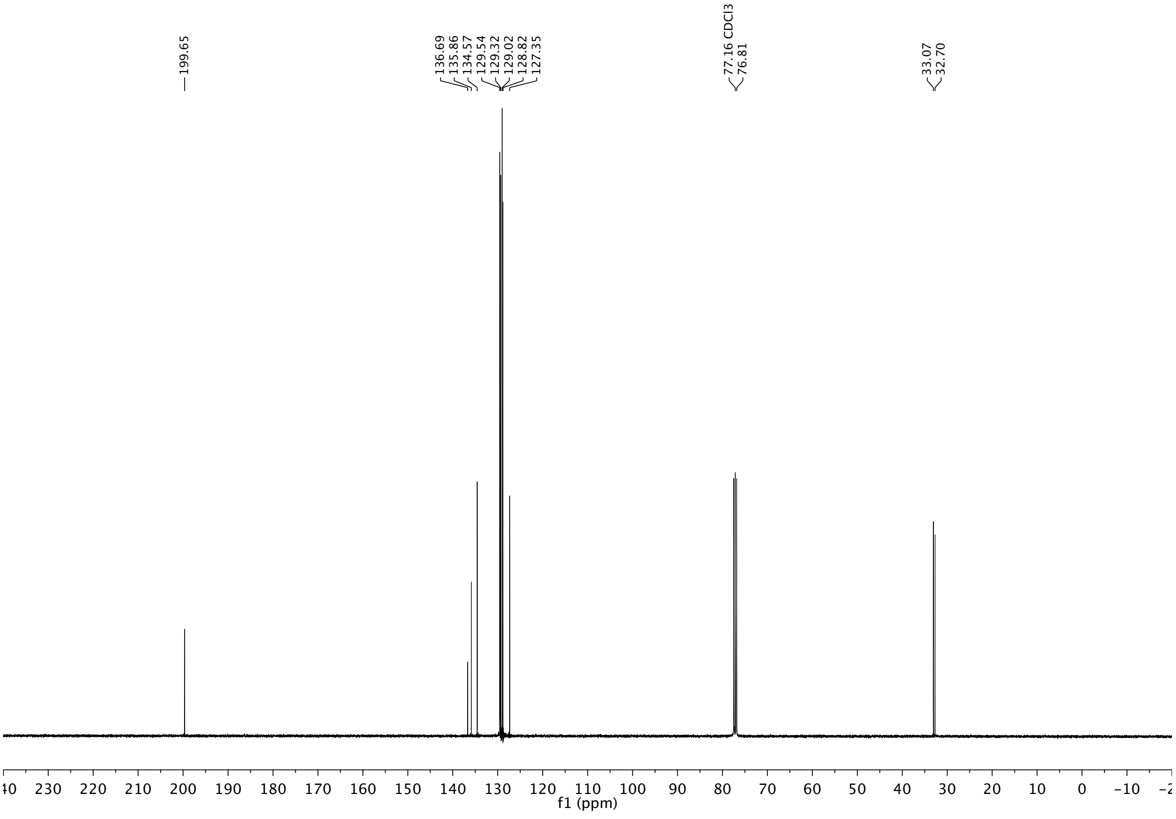

^{13}C NMR (101 MHz, $CDCl_3$) spectrum of compound **1q**.

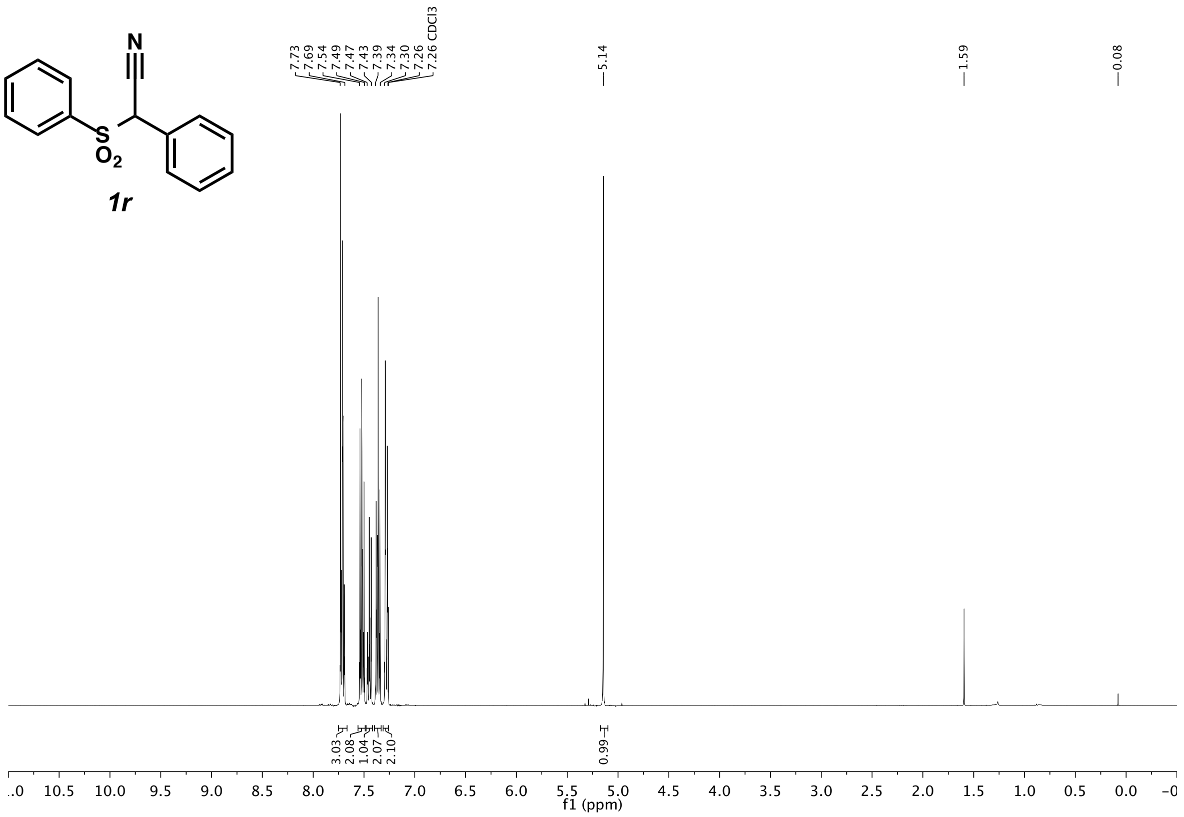

^{1}H NMR (400 MHz, $CDCl_3$) spectrum of compound **1r**.

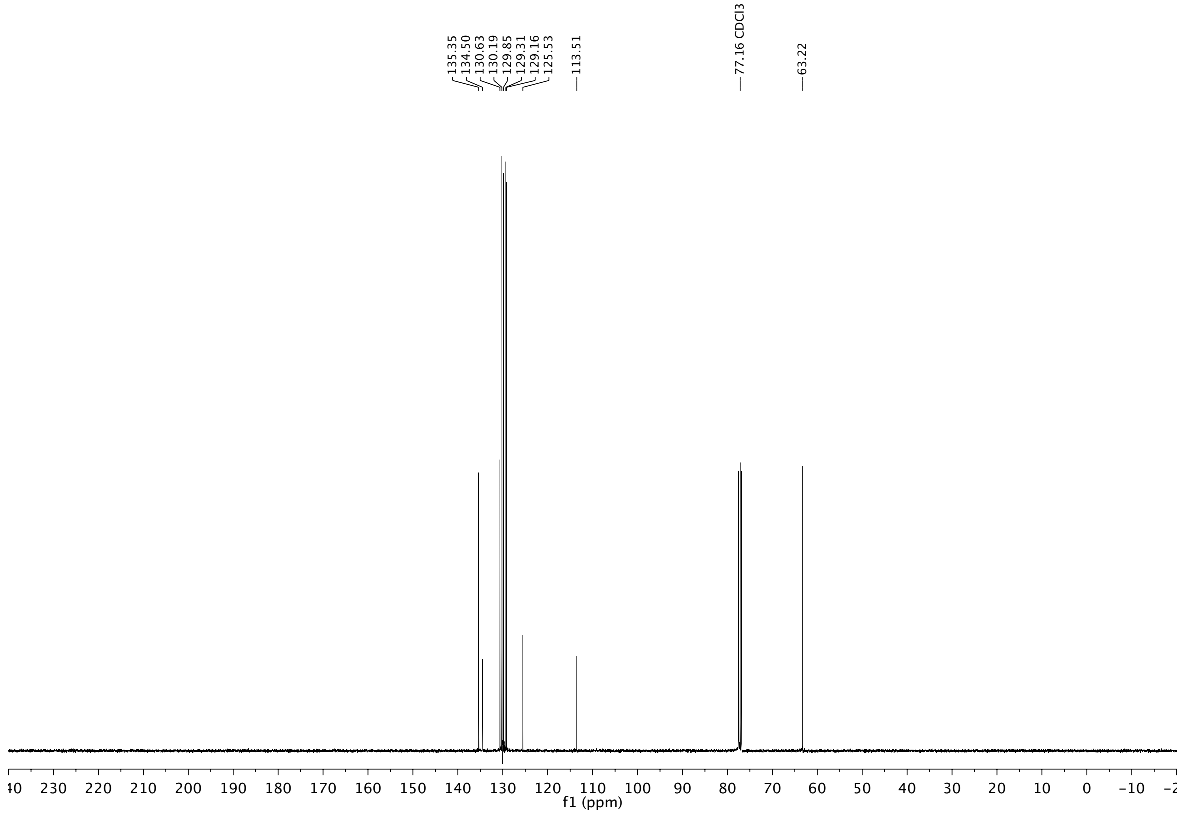

^{13}C NMR (101 MHz, $CDCl_3$) spectrum of compound **1r**.

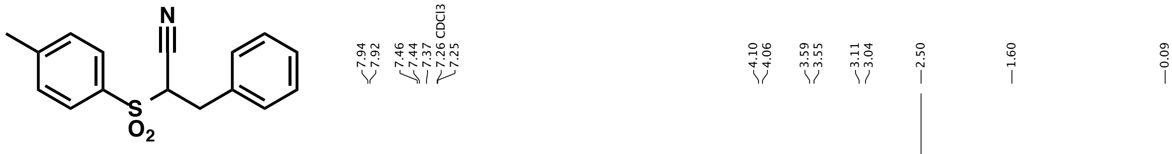

3

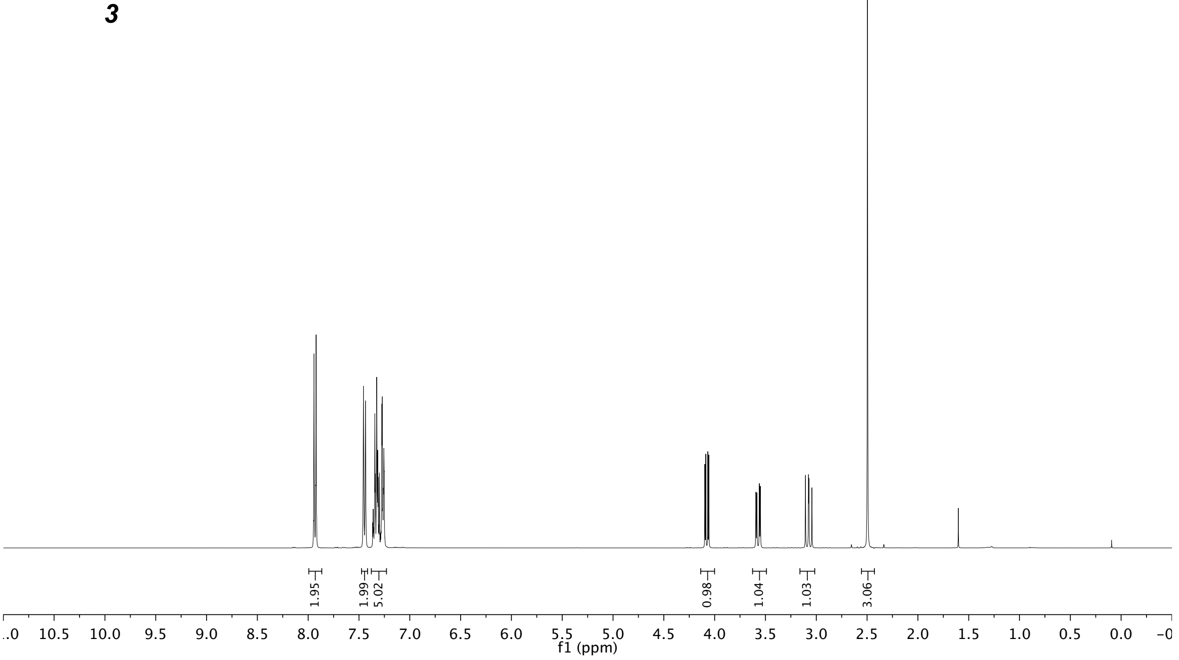

^{1}H NMR (400 MHz, $CDCl_3$) spectrum of compound **3**.

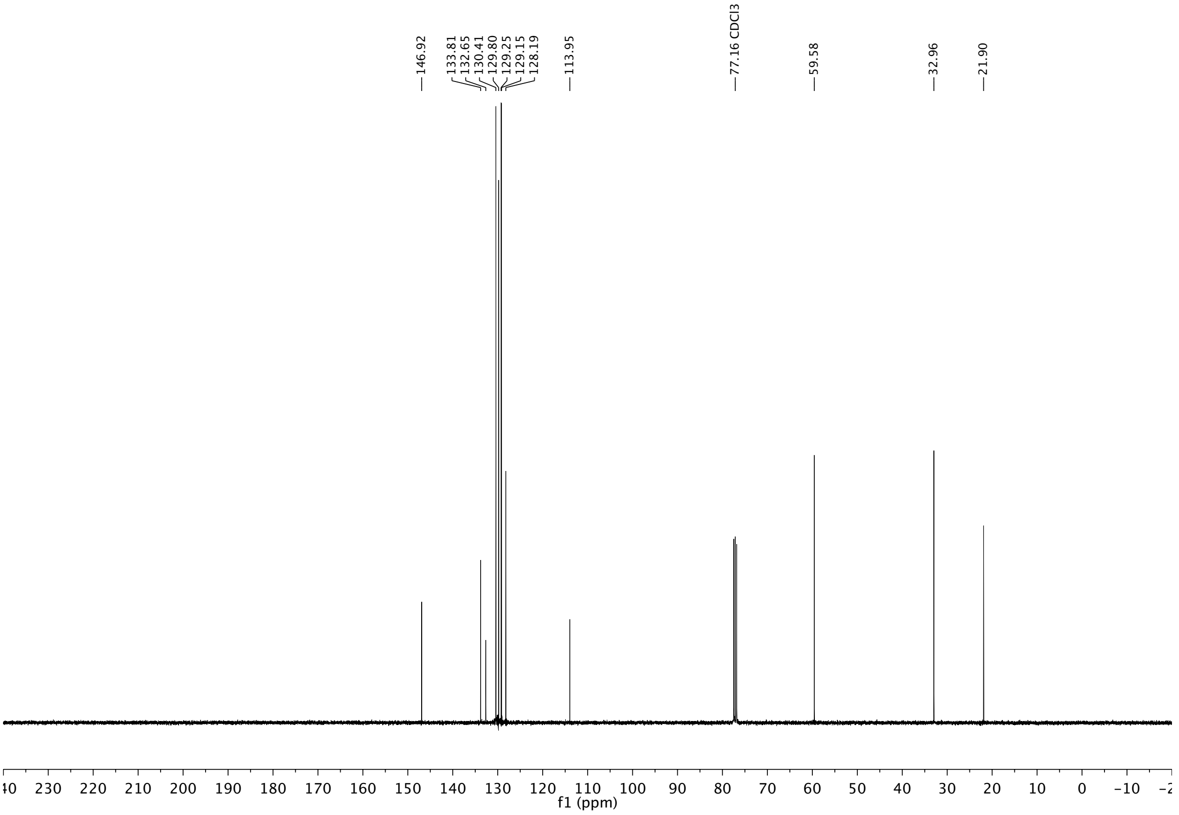

^{13}C NMR (101 MHz, $CDCl_3$) spectrum of compound **3**.

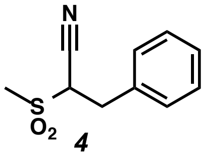

4

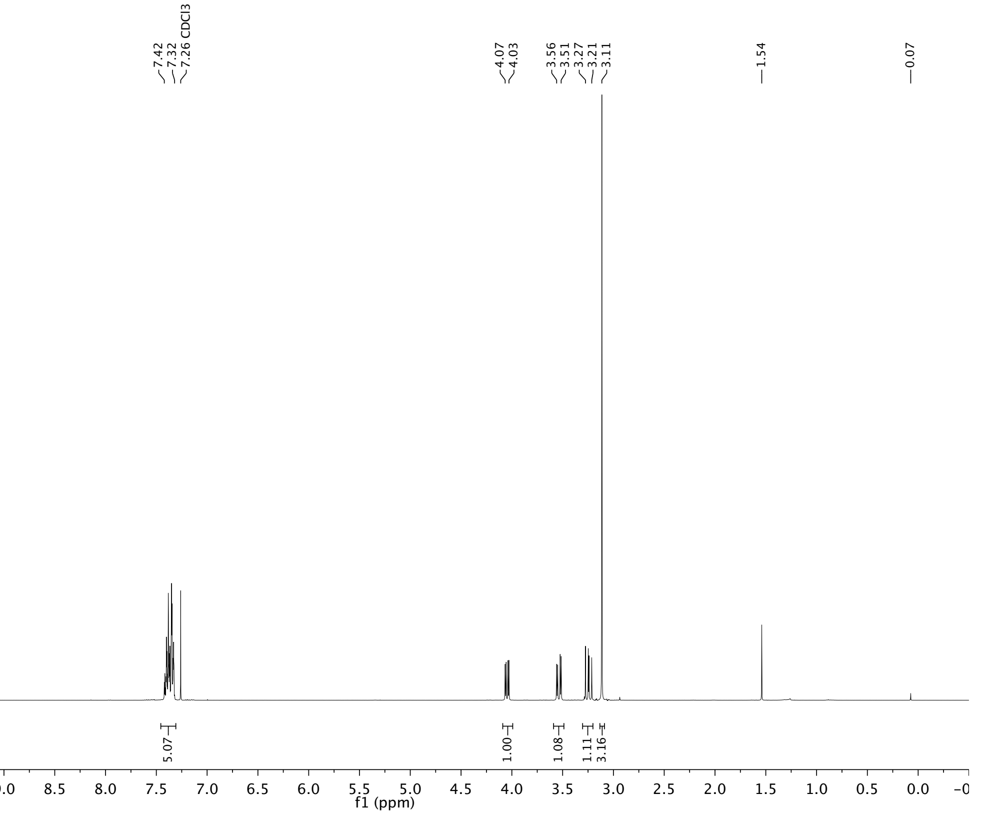

^{1}H NMR (400 MHz, CDCl$_3$) spectrum of compound **4**.

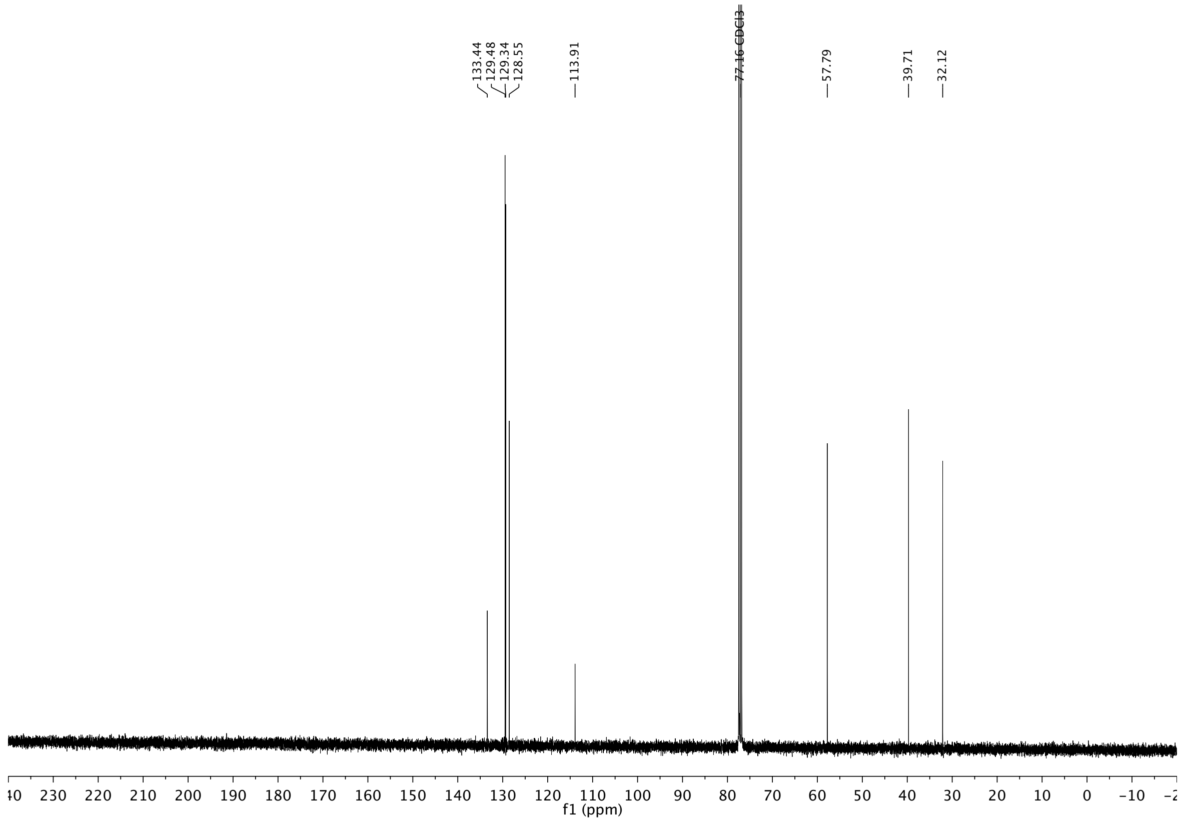

^{3}C NMR (101 MHz, CDCl$_3$) spectrum of compound **4**.

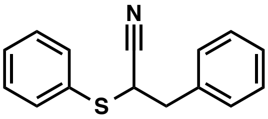

5

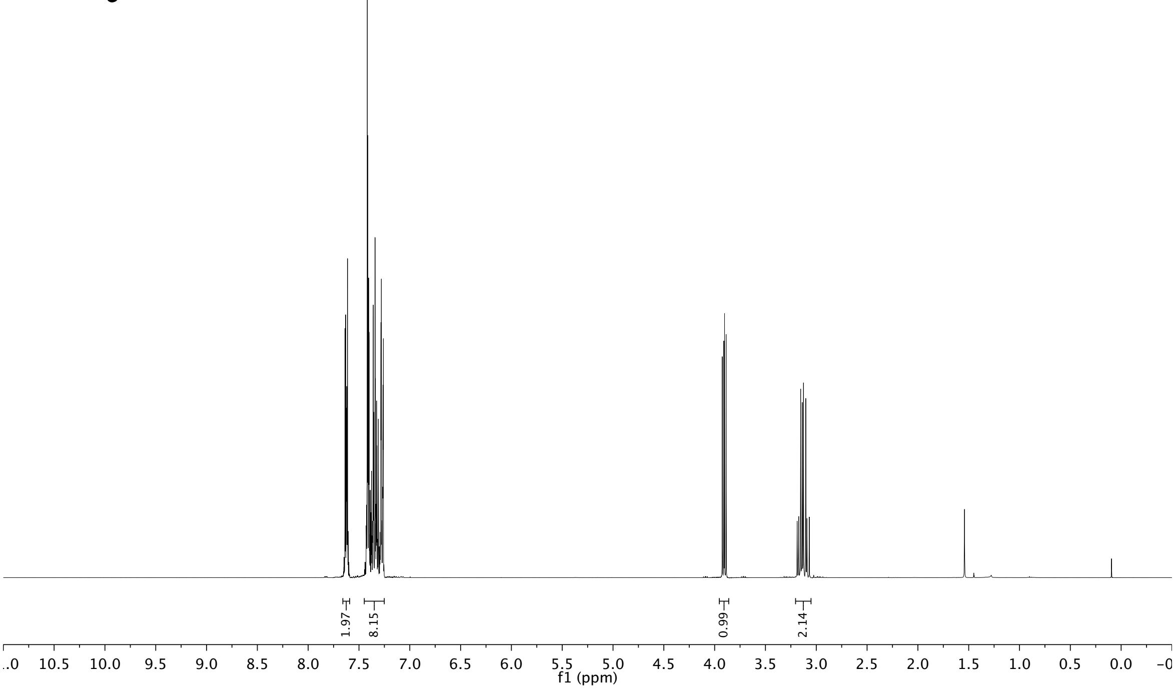

^{1}H NMR (400 MHz, $CDCl_3$) spectrum of compound **5**.

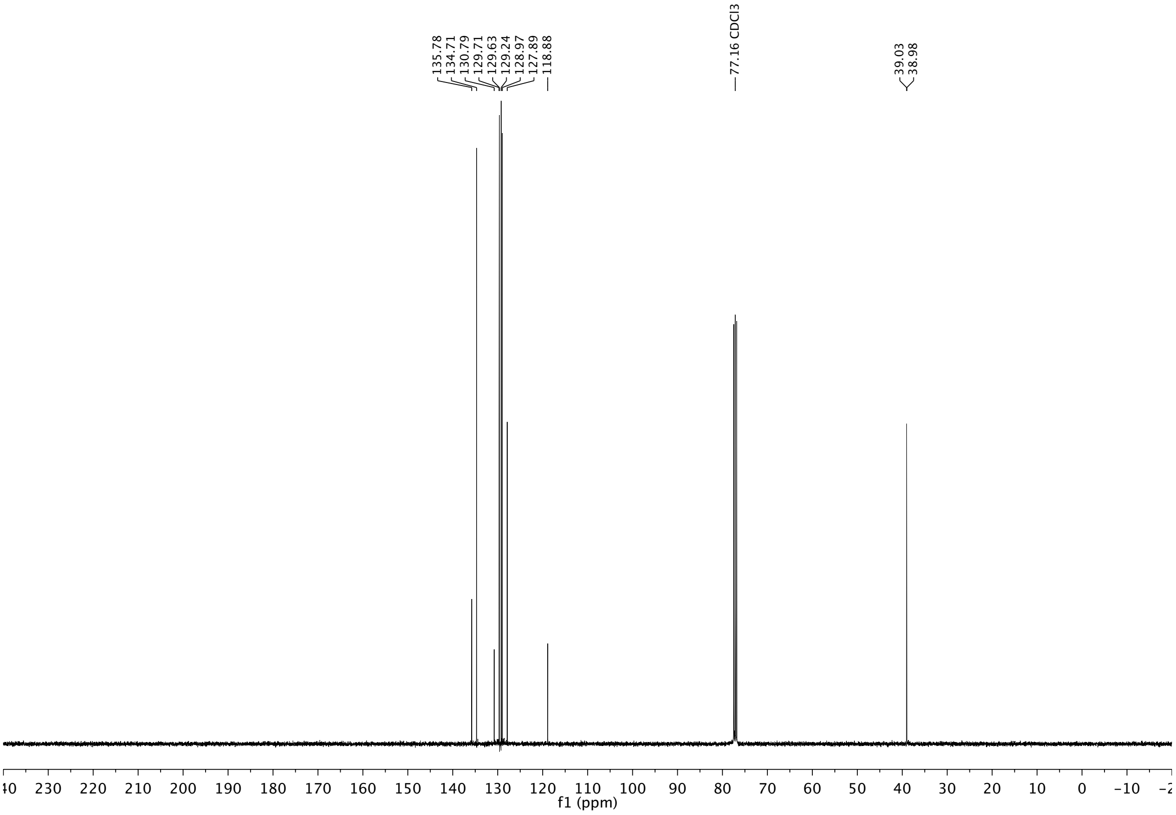

^{13}C NMR (101 MHz, $CDCl_3$) spectrum of compound **5**.

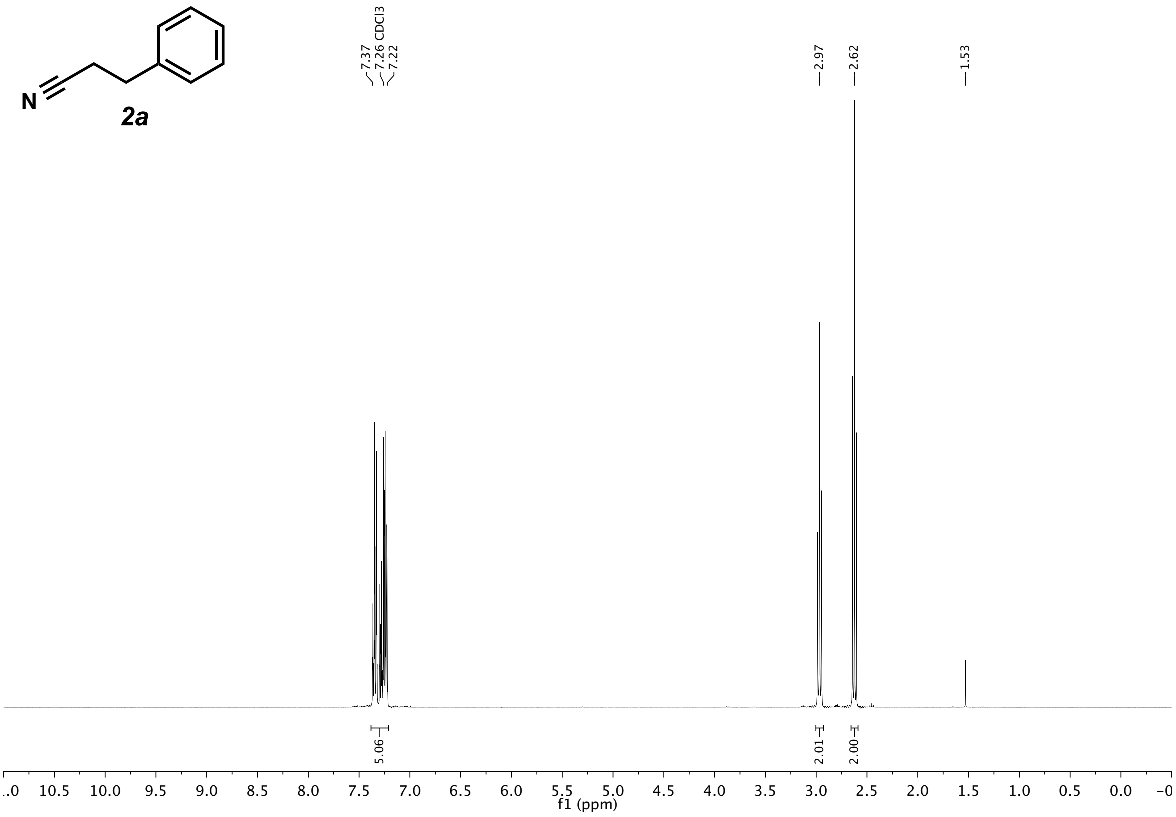

^{1}H NMR (400 MHz, $CDCl_3$) spectrum of compound **2a**.

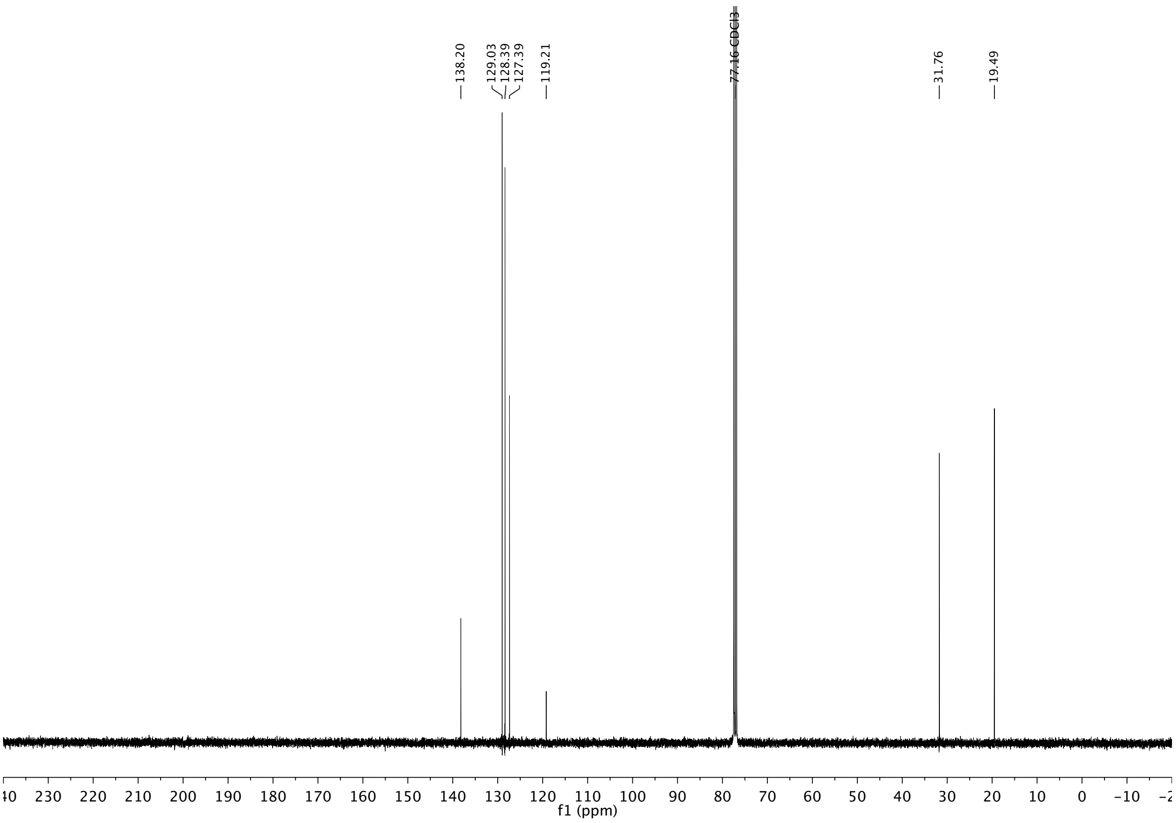

^{13}C NMR (101 MHz, $CDCl_3$) spectrum of compound **2a**.

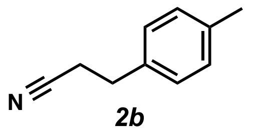

2b

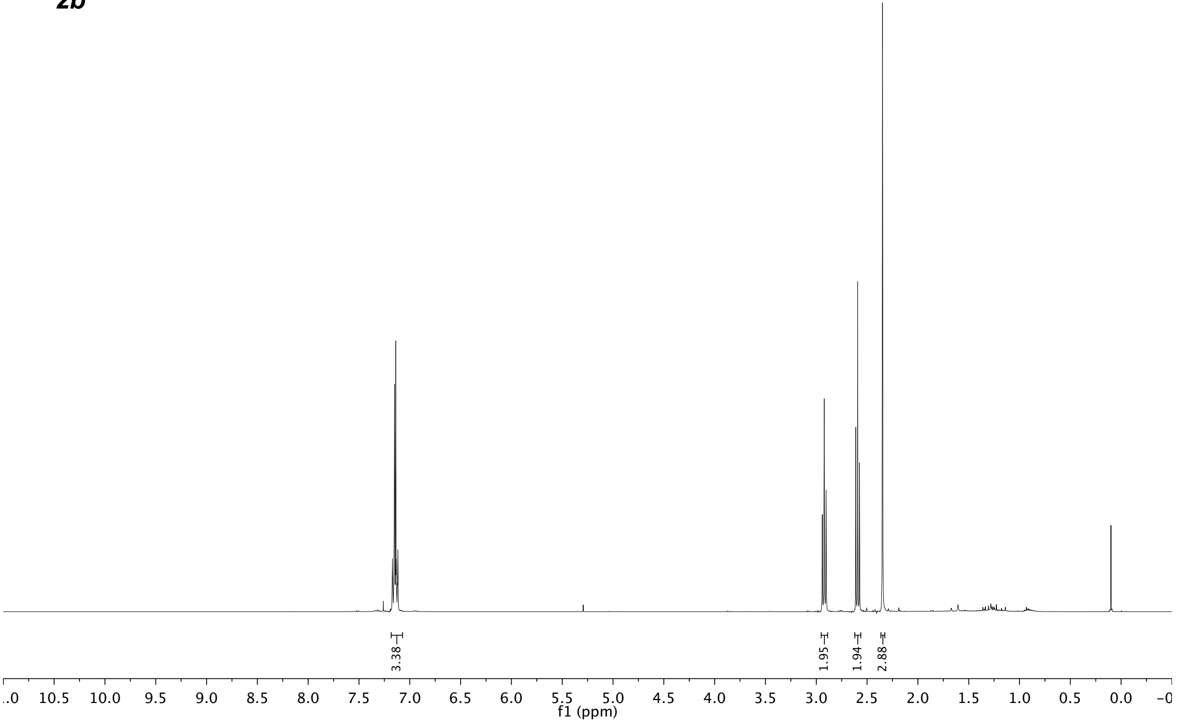

^{1}H NMR (400 MHz, $CDCl_3$) spectrum of compound **2b**.

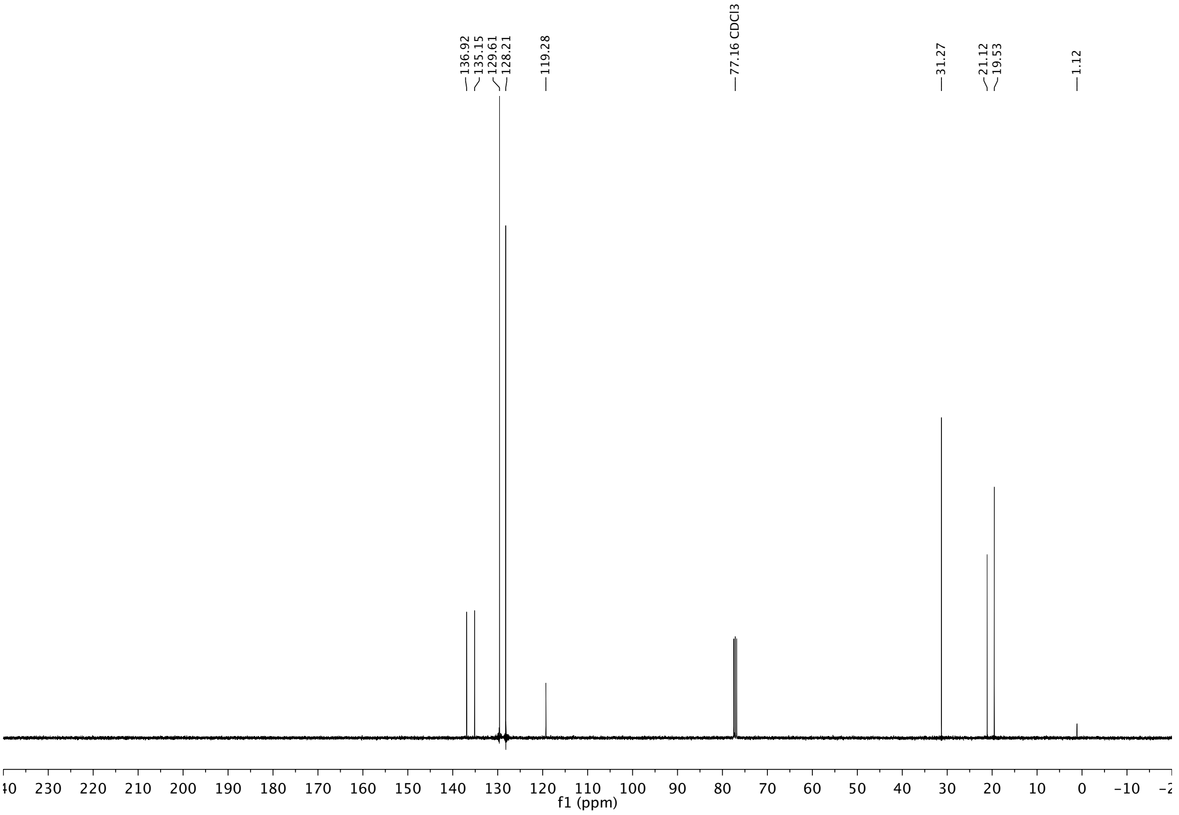

^{13}C NMR (101 MHz, $CDCl_3$) spectrum of compound **2b**.

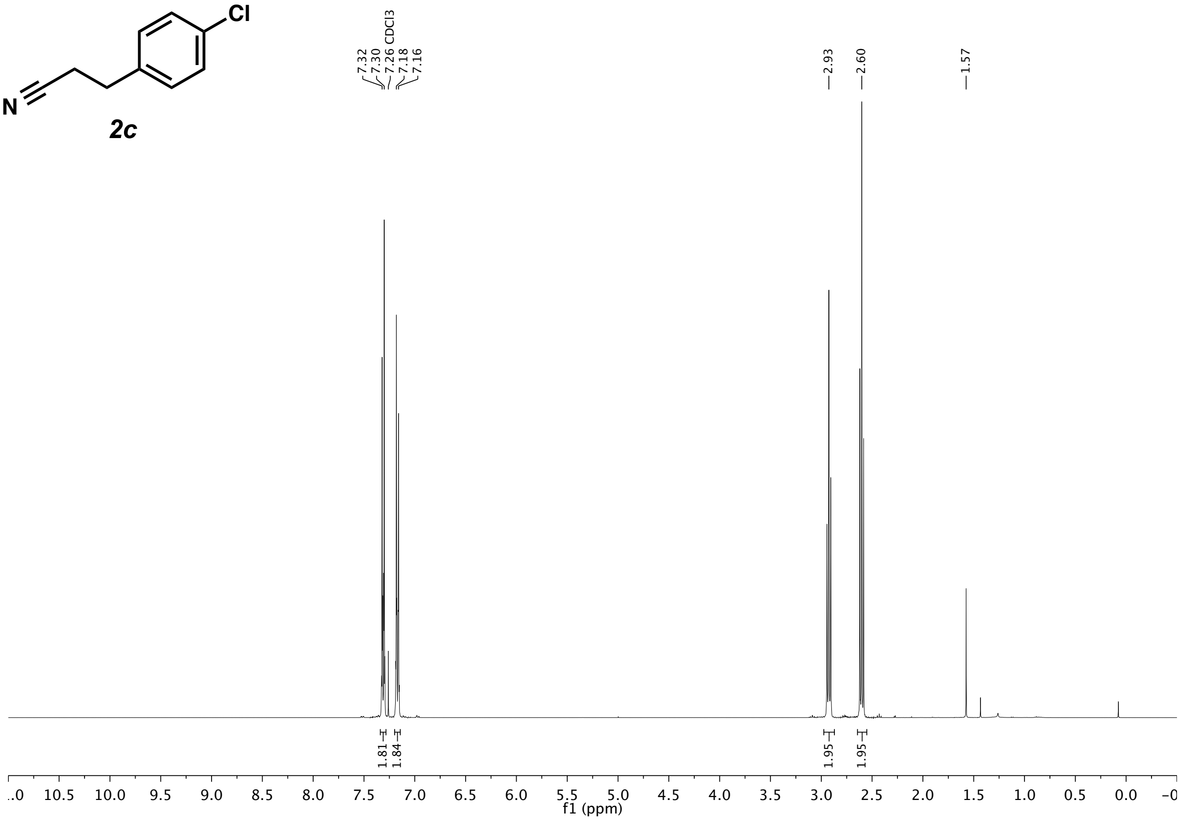

^{1}H NMR (400 MHz, $CDCl_3$) spectrum of compound **2c**.

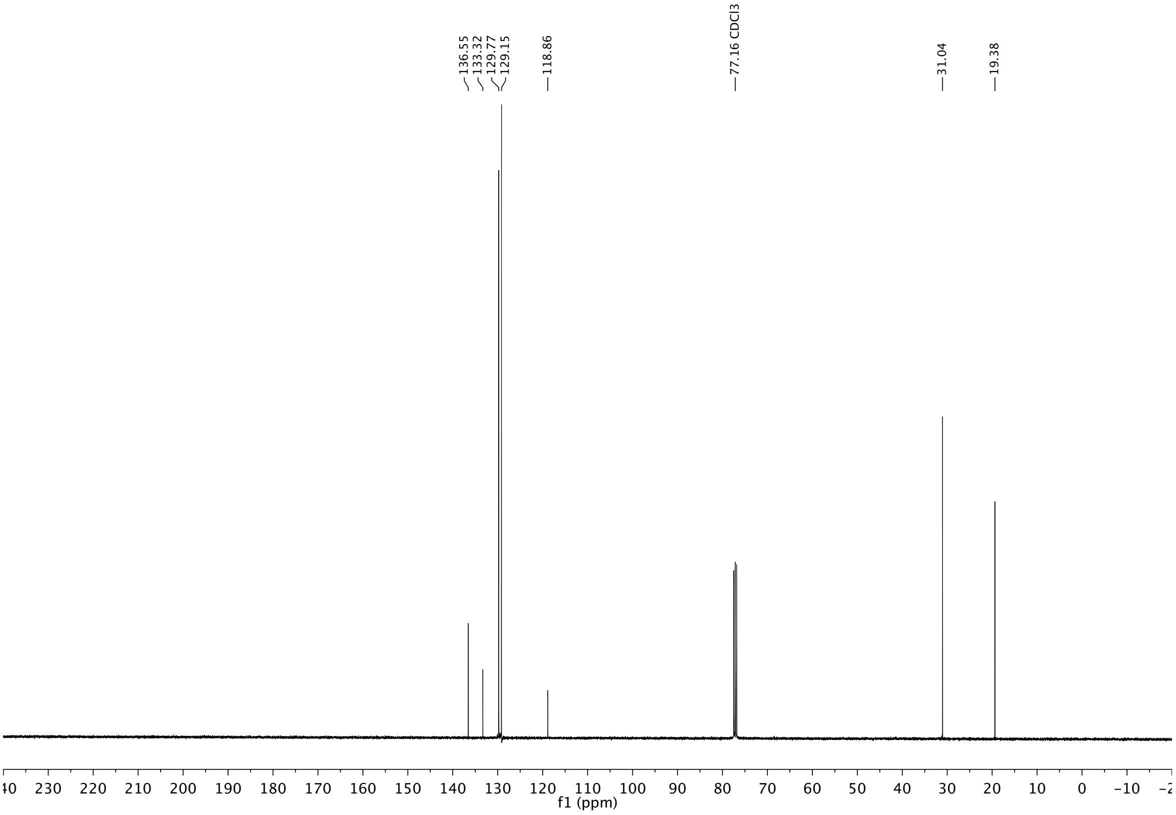

^{13}C NMR (101 MHz, $CDCl_3$) spectrum of compound **2c**.

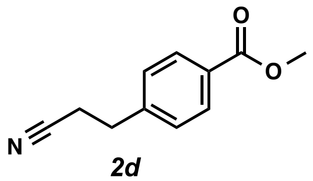

2d

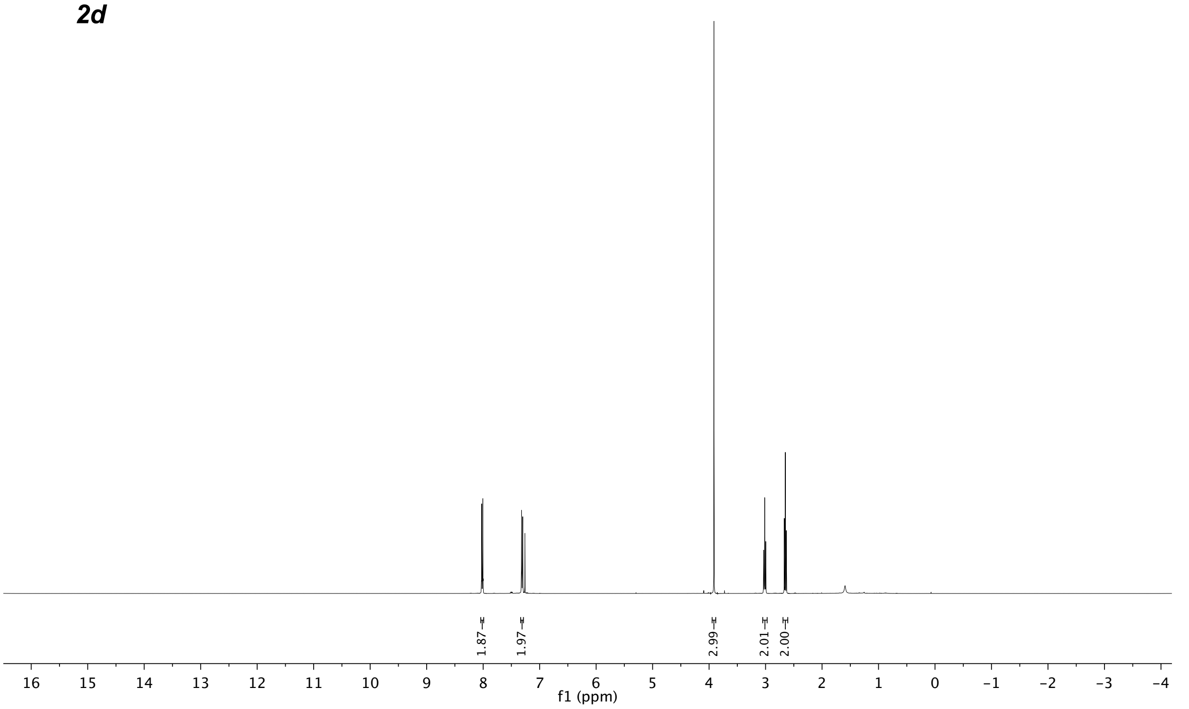

^{1}H NMR (400 MHz, $CDCl_3$) spectrum of compound **2d**.

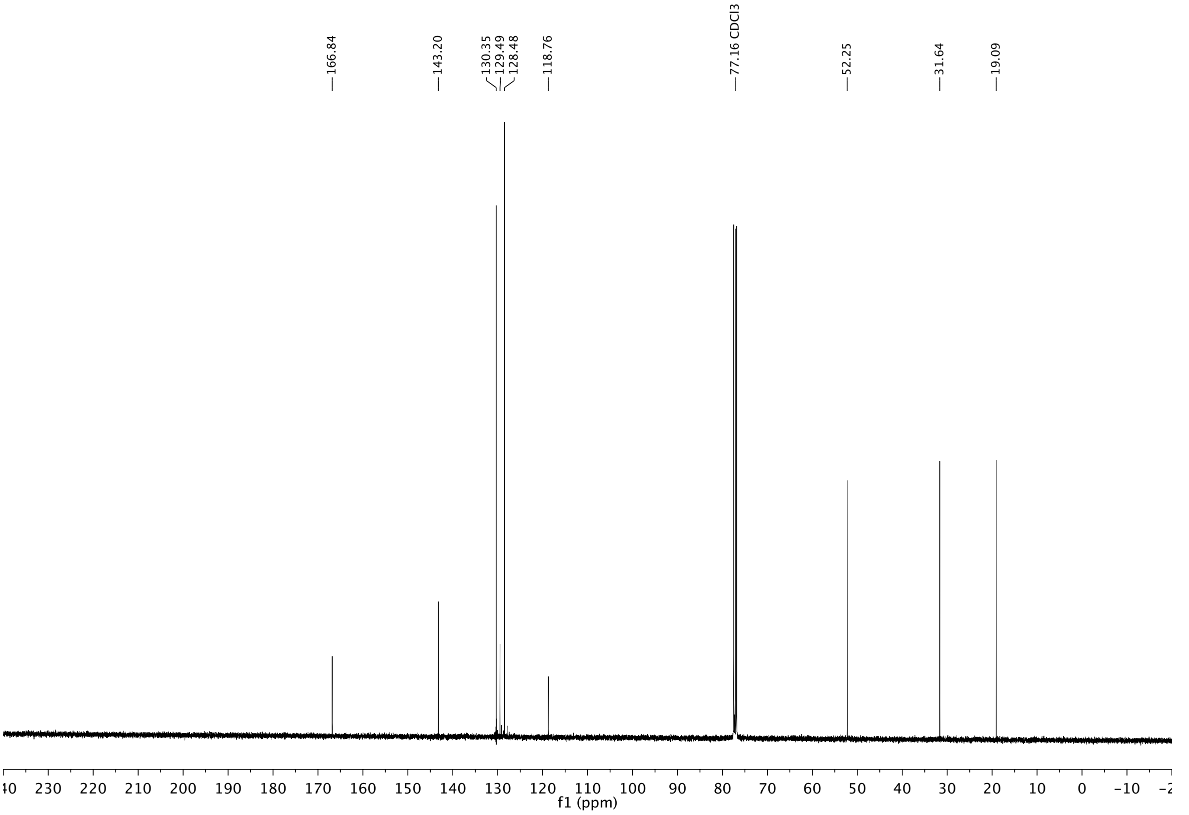

^{13}C NMR (101 MHz, $CDCl_3$) spectrum of compound **2d**.

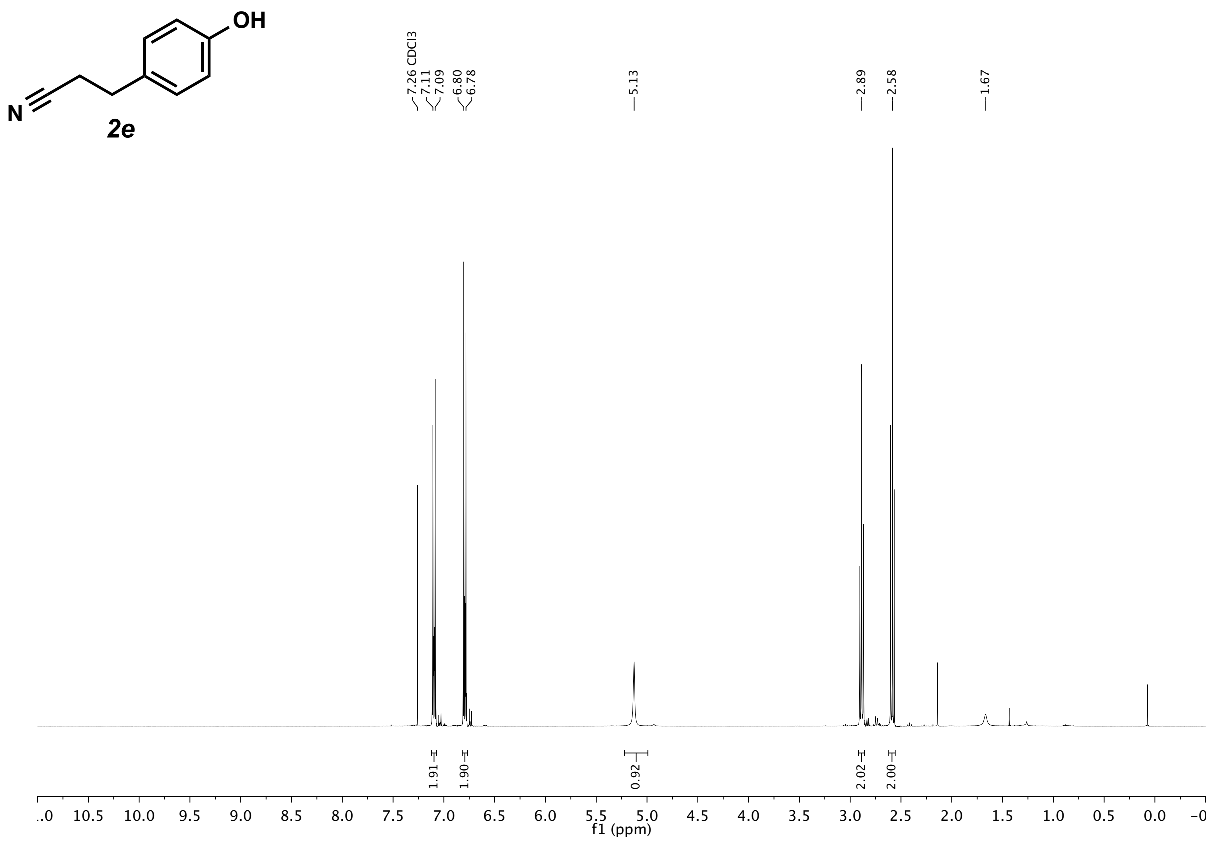

^{1}H NMR (400 MHz, $CDCl_3$) spectrum of compound **2e**.

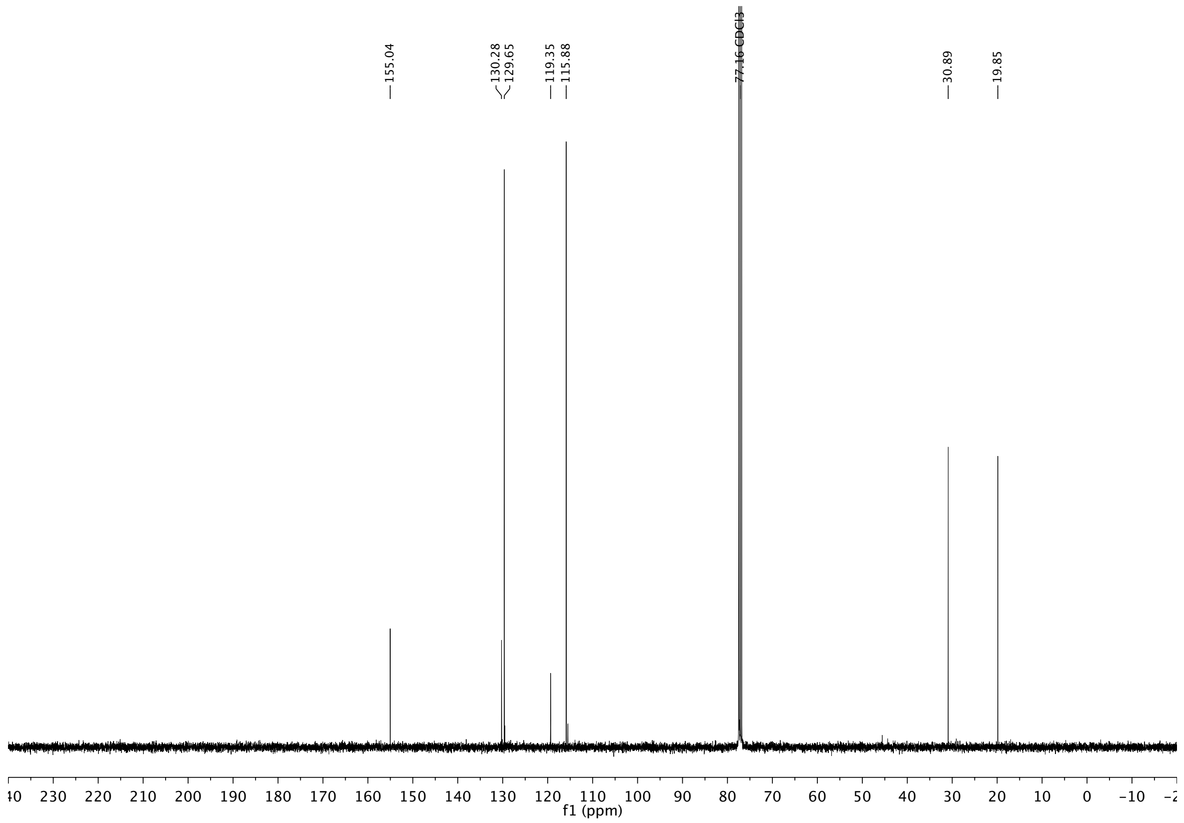

^{13}C NMR (101 MHz, $CDCl_3$) spectrum of compound **2e**.

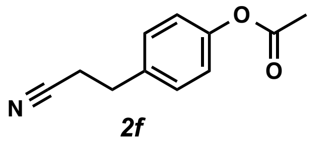

2f

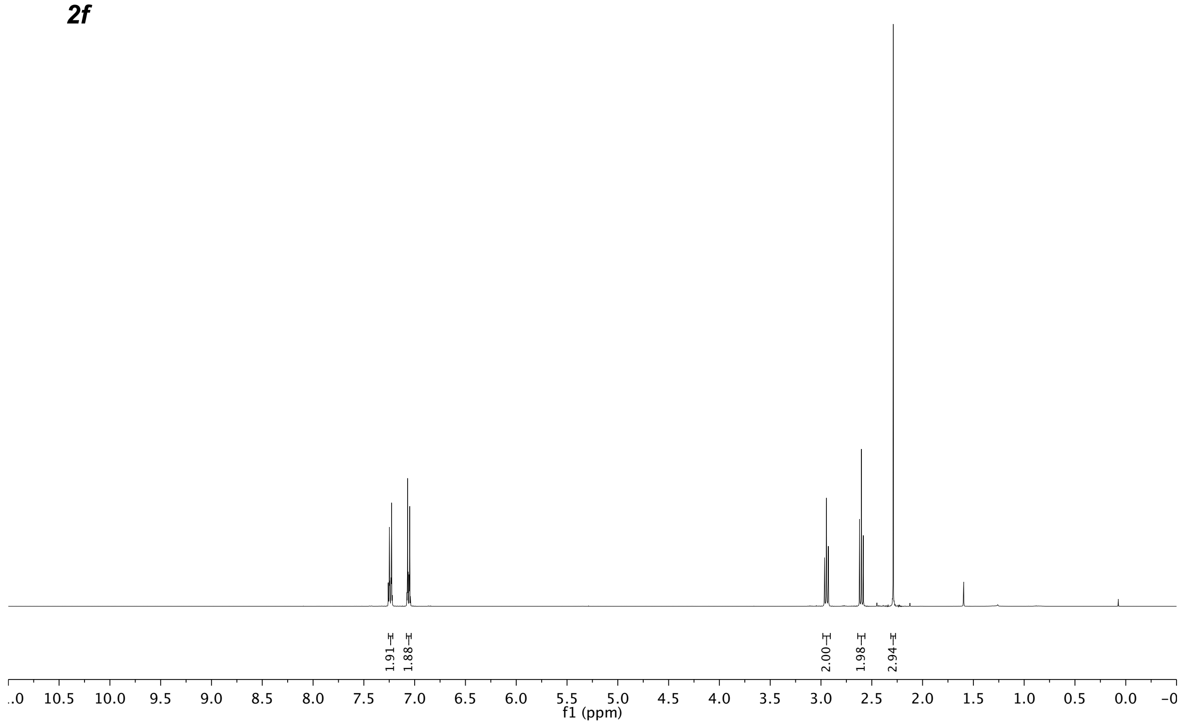

^{1}H NMR (400 MHz, $CDCl_3$) spectrum of compound **2f**.

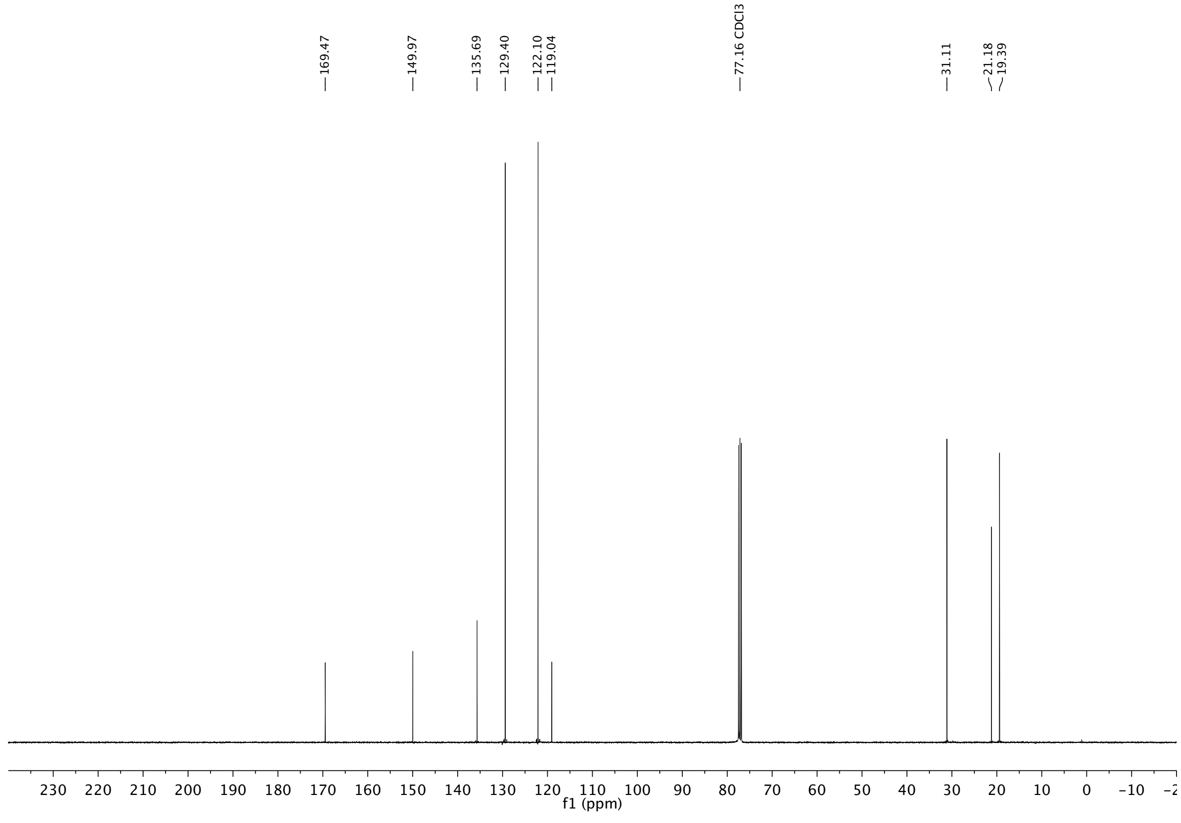

^{13}C NMR (101 MHz, $CDCl_3$) spectrum of compound **2f**.

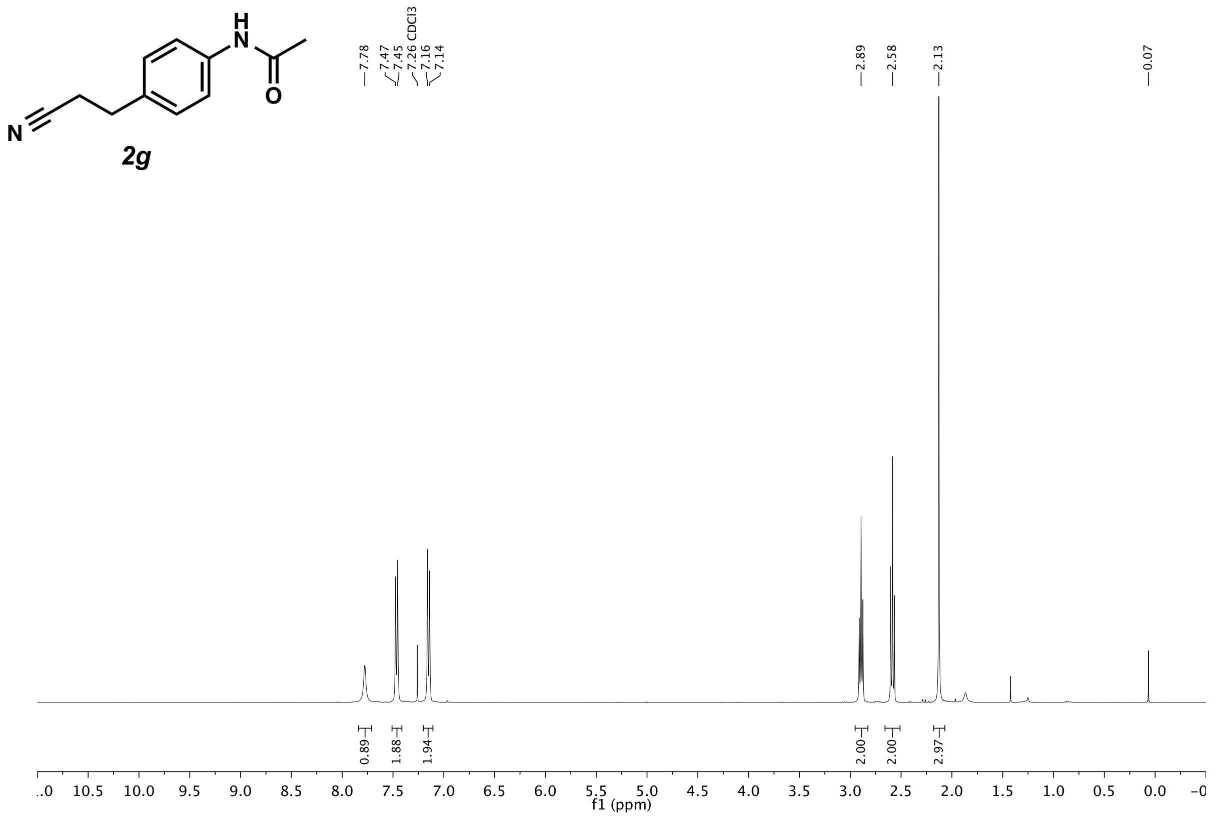

^{1}H NMR (400 MHz, $CDCl_3$) spectrum of compound **2g**.

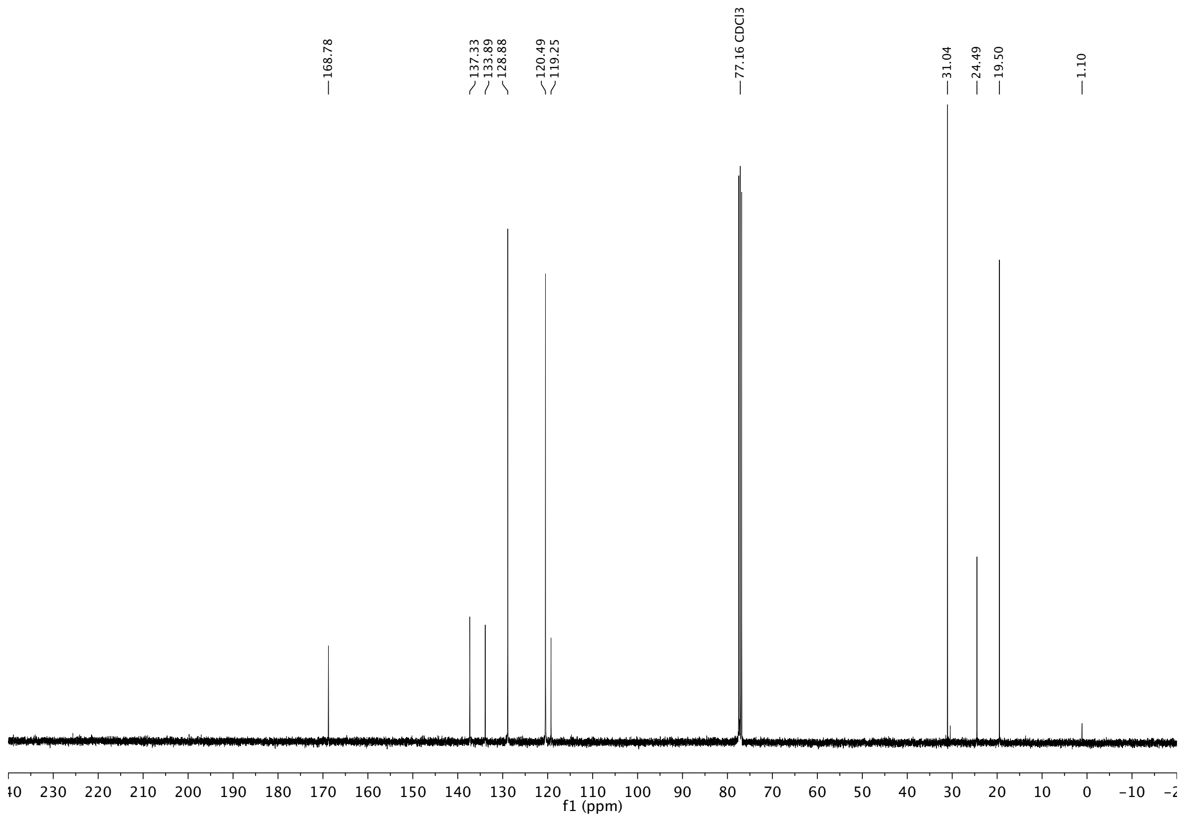

^{13}C NMR (101 MHz, $CDCl_3$) spectrum of compound **2g**.

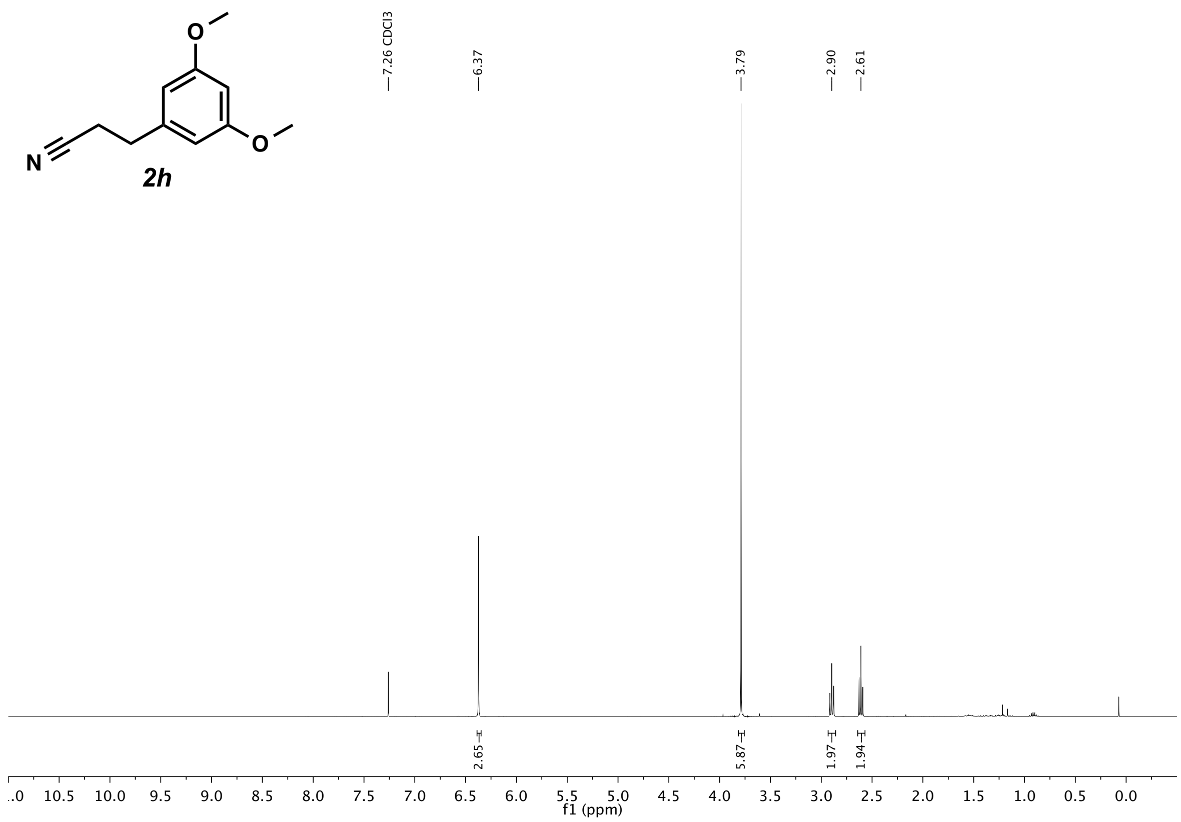

^1H NMR (400 MHz, $CDCl_3$) spectrum of compound **2h**.

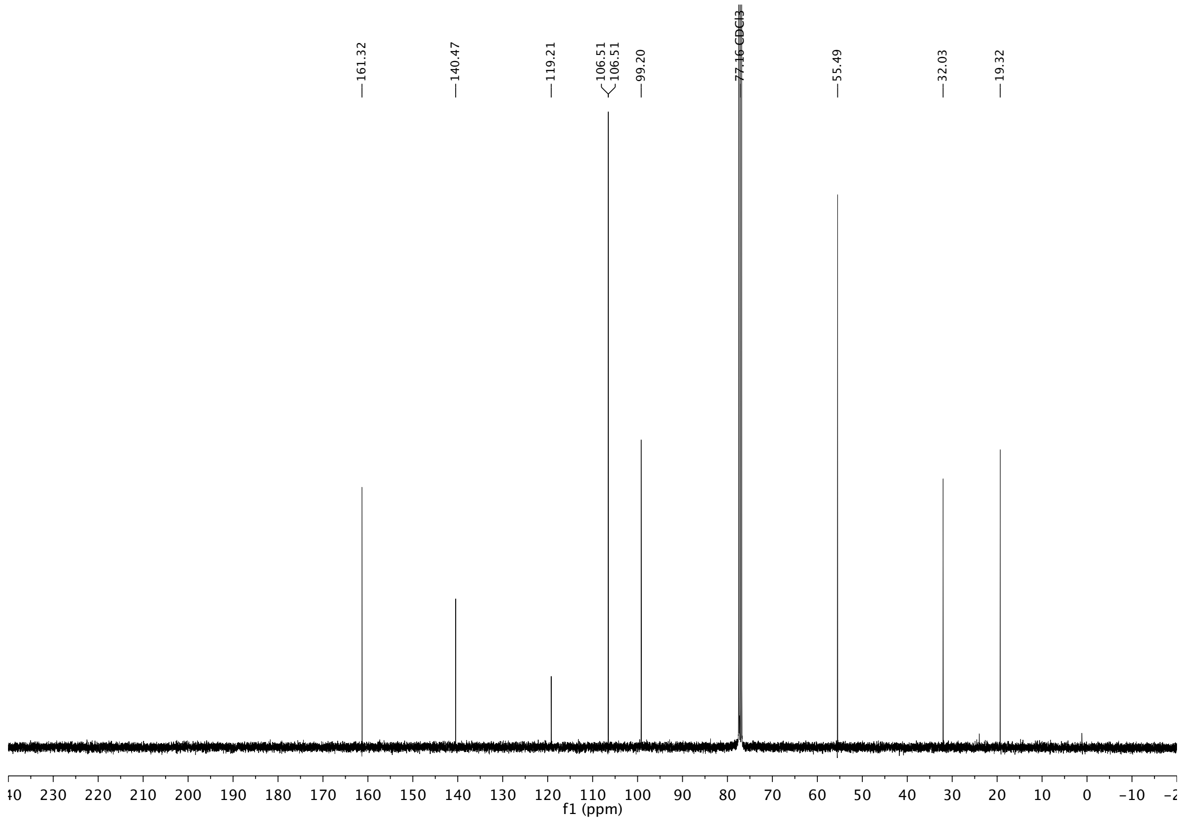

^{13}C NMR (101 MHz, $CDCl_3$) spectrum of compound **2h**.

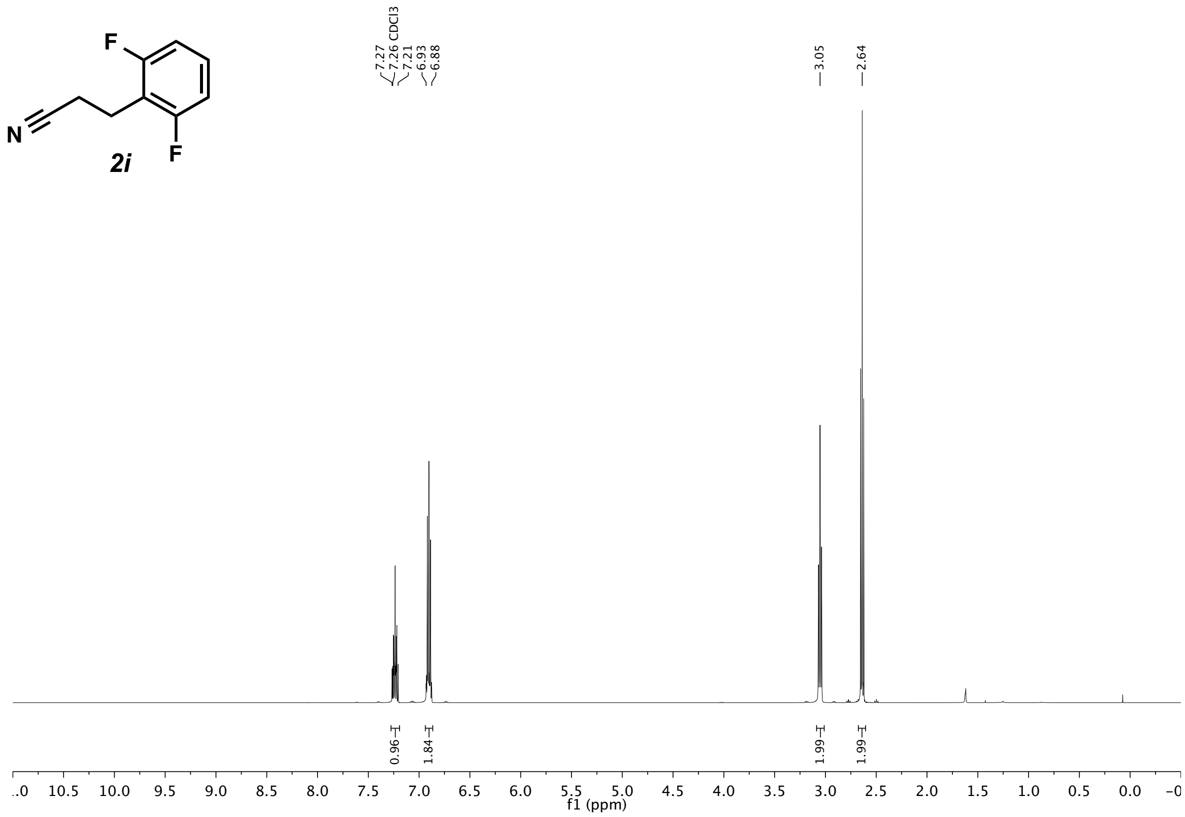

^{1}H NMR (500 MHz, $CDCl_3$) spectrum of compound **2i**.

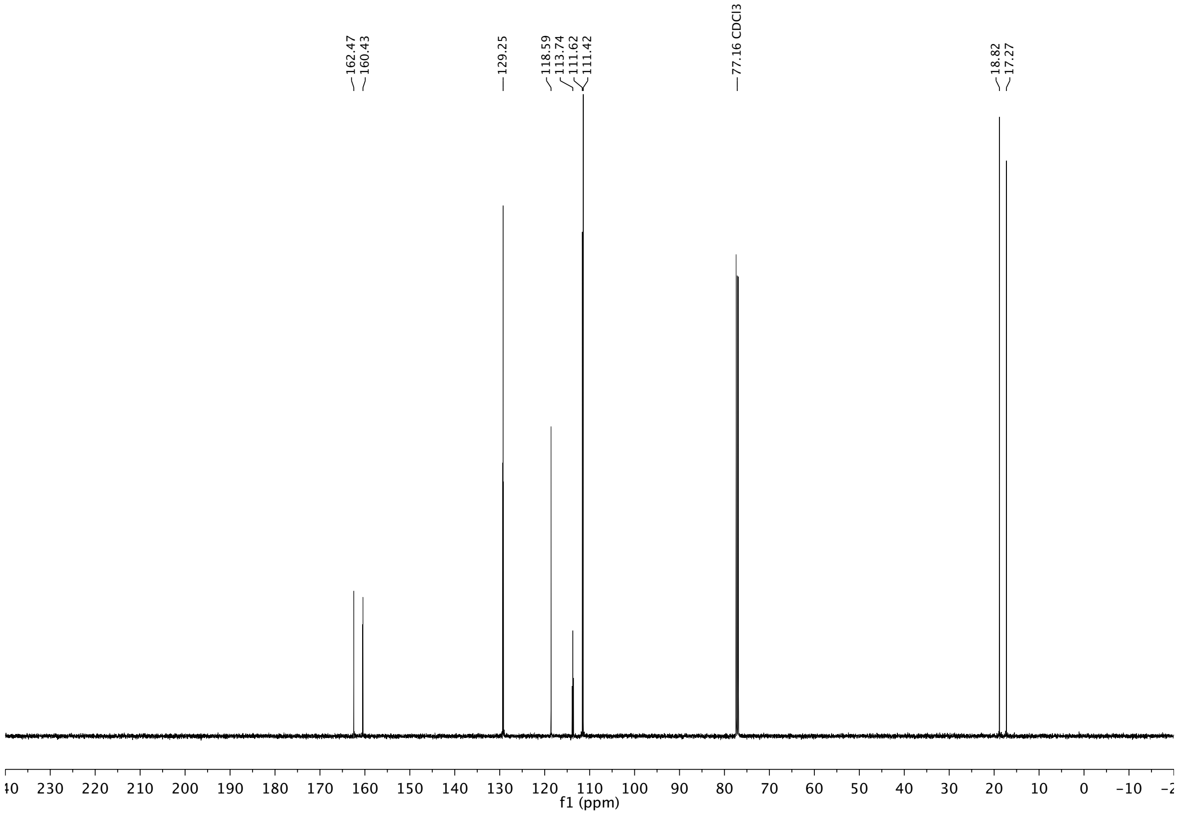

^{13}C NMR (126 MHz, $CDCl_3$) spectrum of compound **2i**.

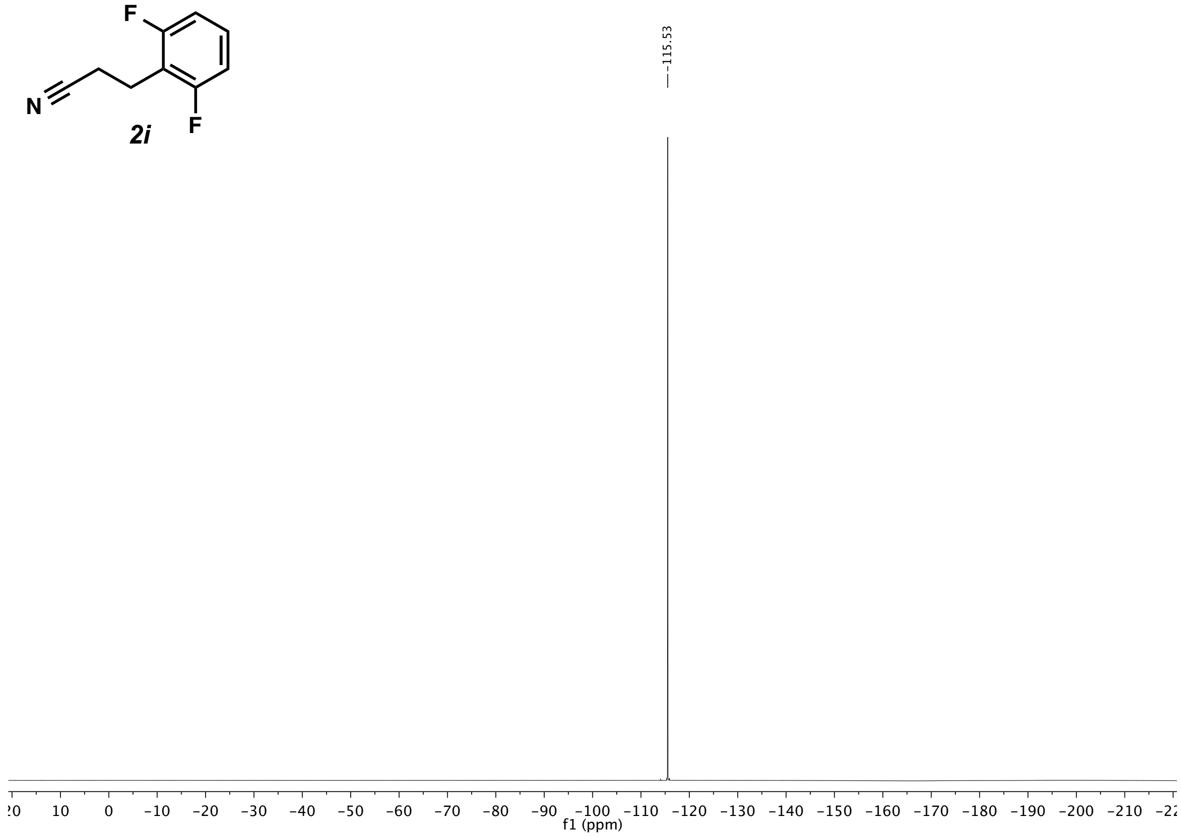

^{19}F NMR (470 MHz, $CDCl_3$) spectrum of compound **2i**.

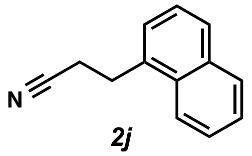

2j

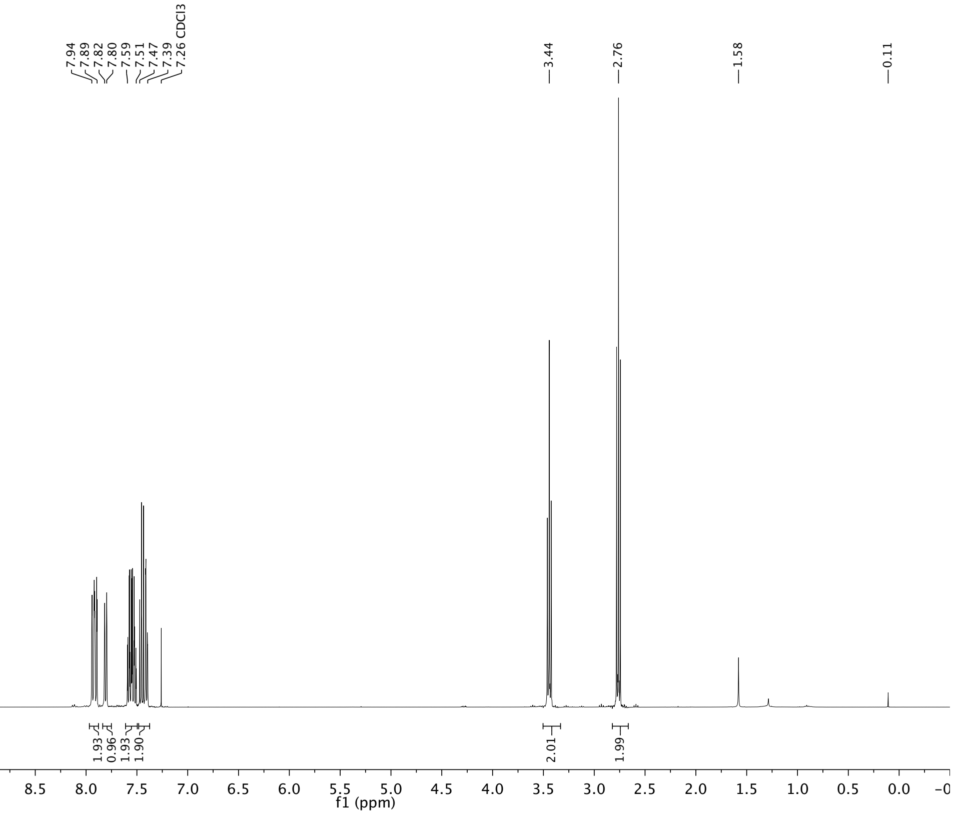

^{1}H NMR (400 MHz, $CDCl_3$) spectrum of compound **2j**.

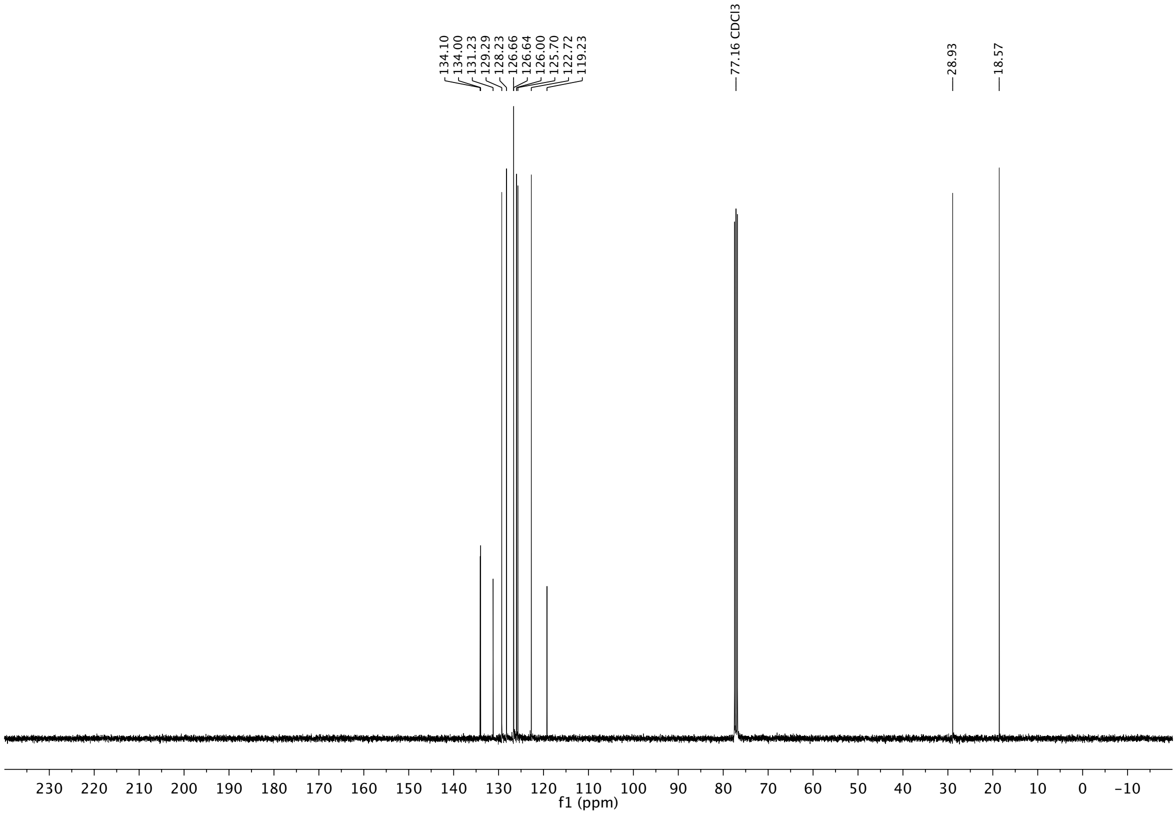

^{13}C NMR (101 MHz, $CDCl_3$) spectrum of compound **2j**.

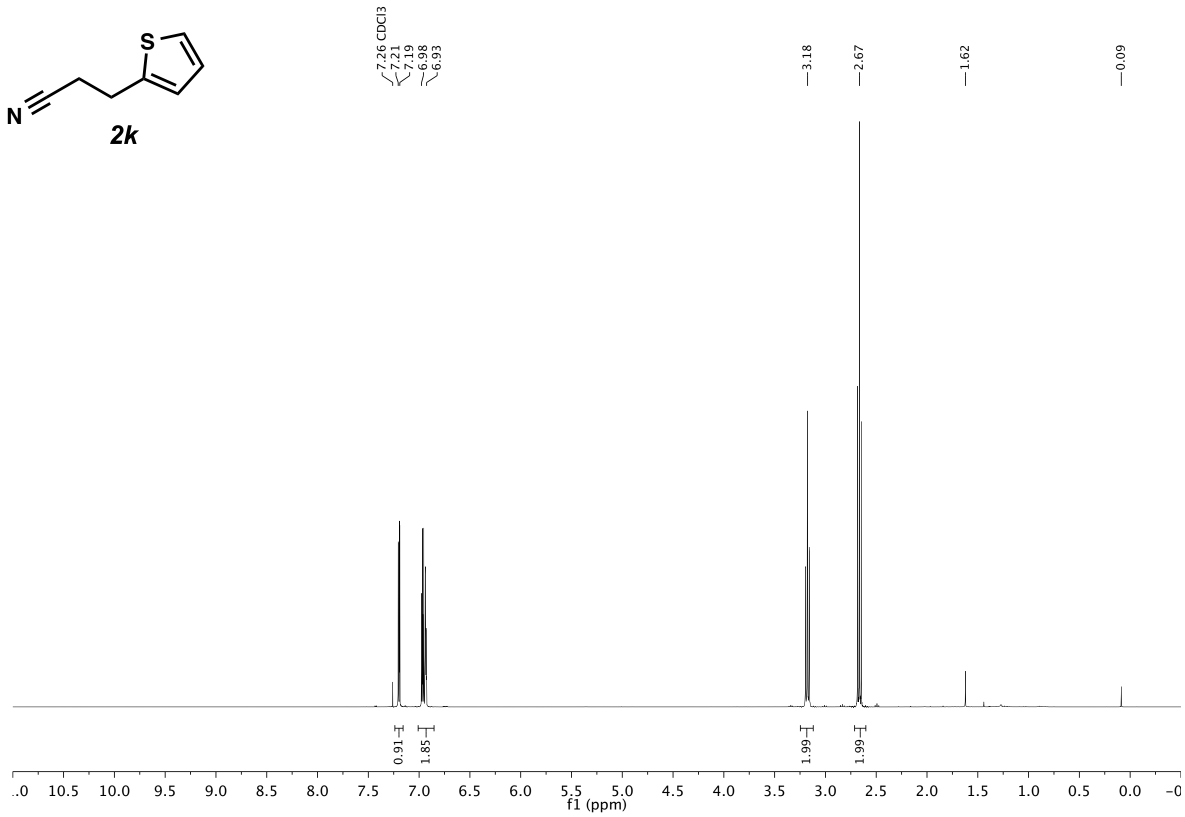

^{1}H NMR (400 MHz, $CDCl_3$) spectrum of compound **2k**.

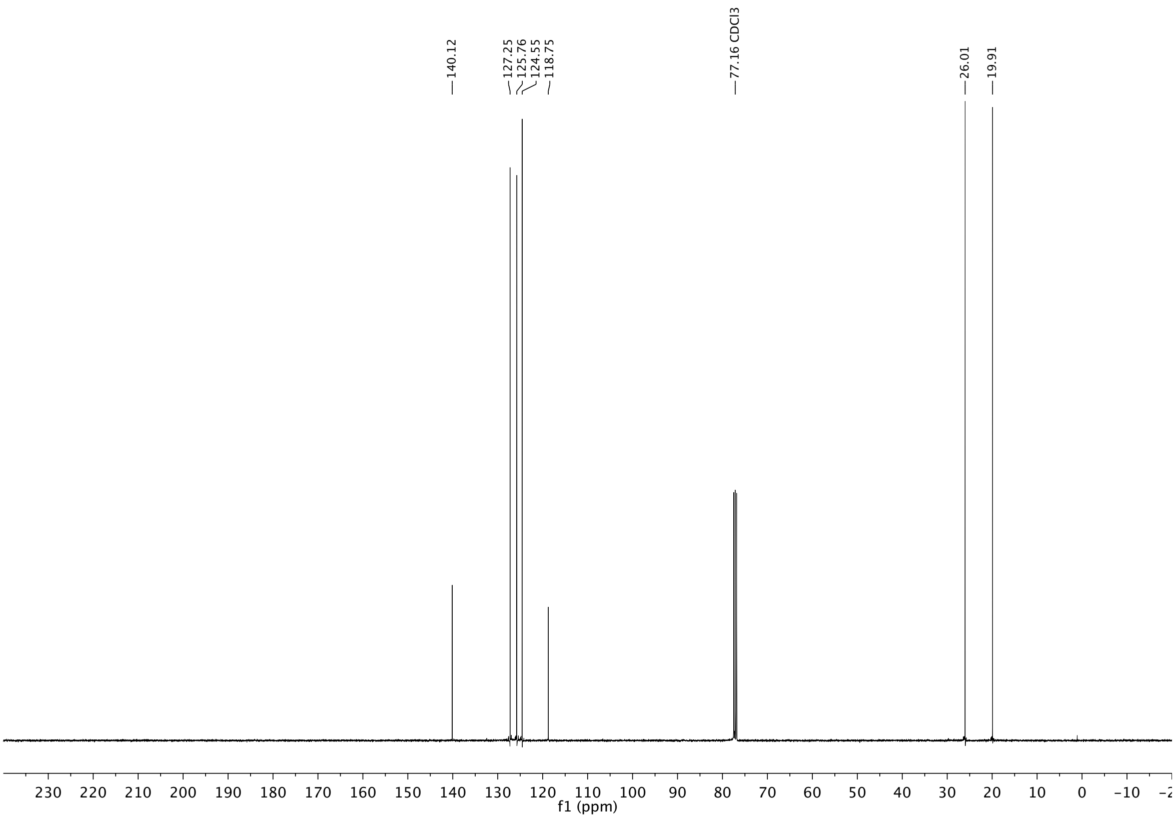

^{13}C NMR (101 MHz, $CDCl_3$) spectrum of compound **2k**.

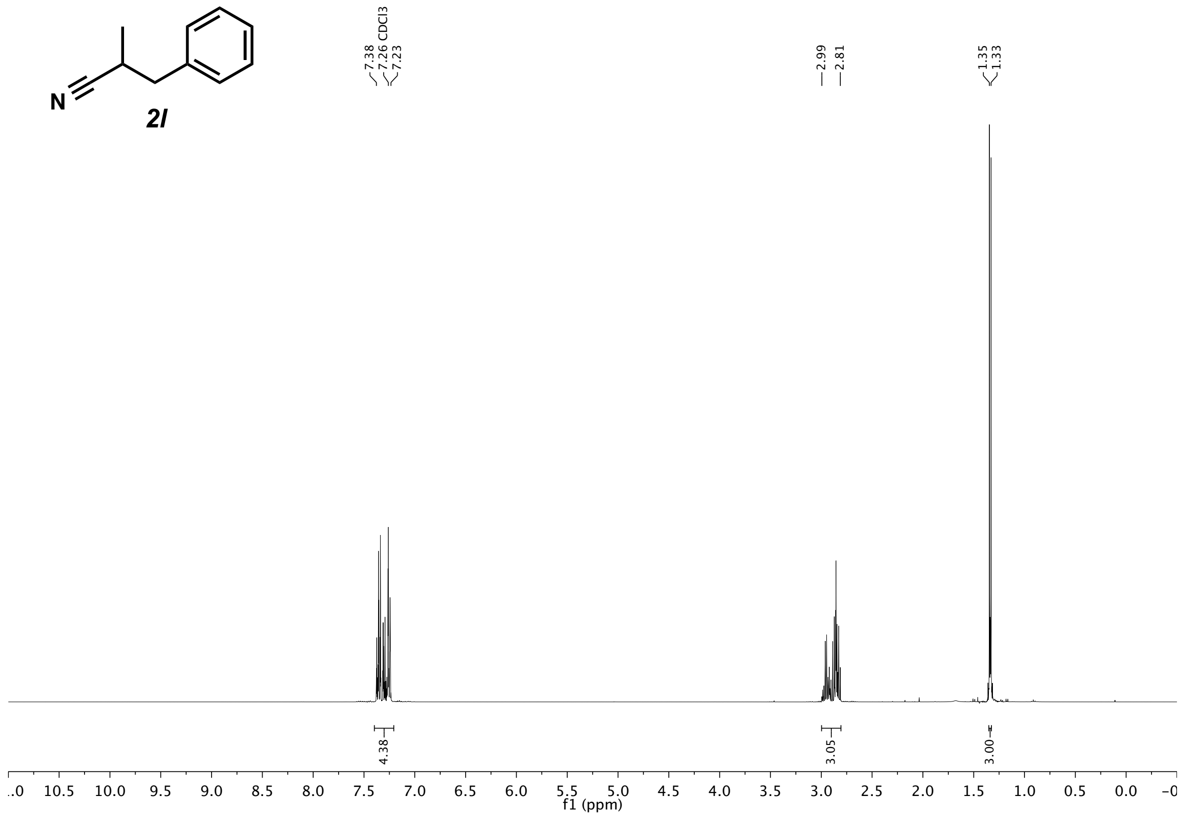

^{1}H NMR (400 MHz, $CDCl_3$) spectrum of compound **2l**.

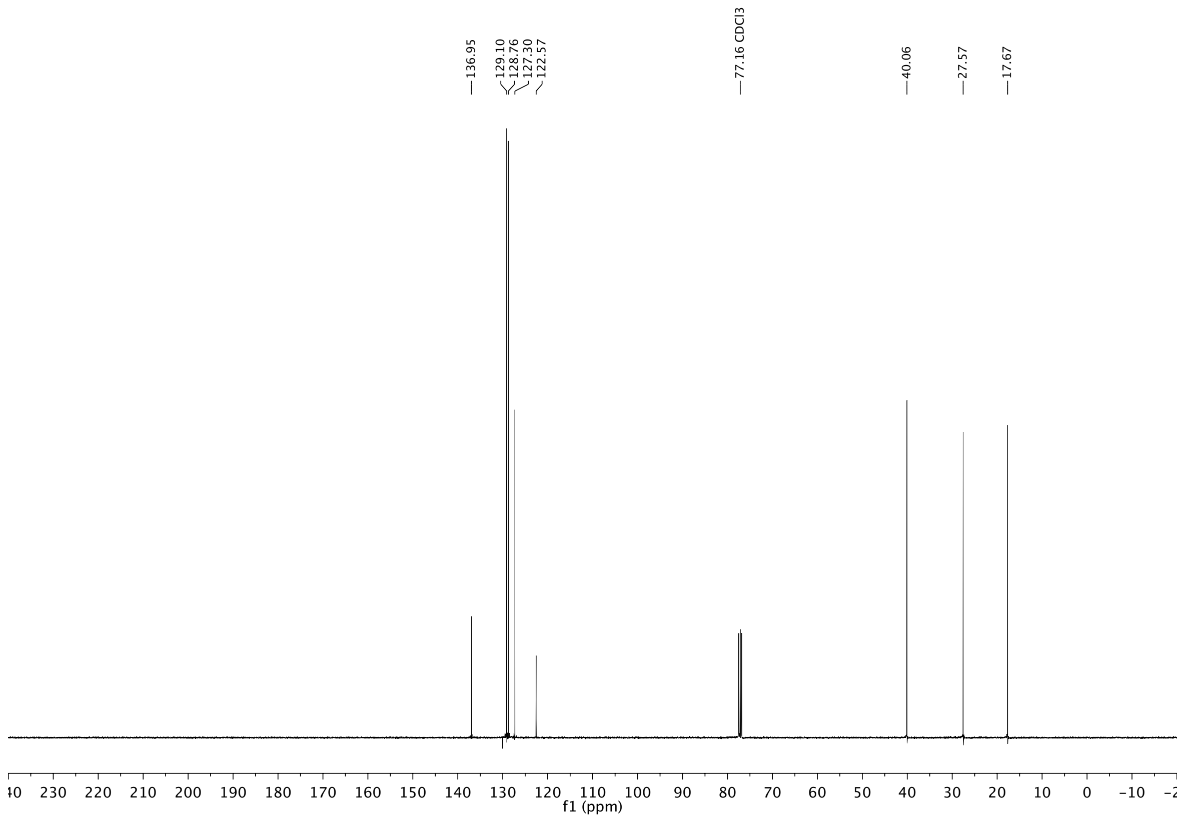

^{13}C NMR (101 MHz, $CDCl_3$) spectrum of compound **2l**.

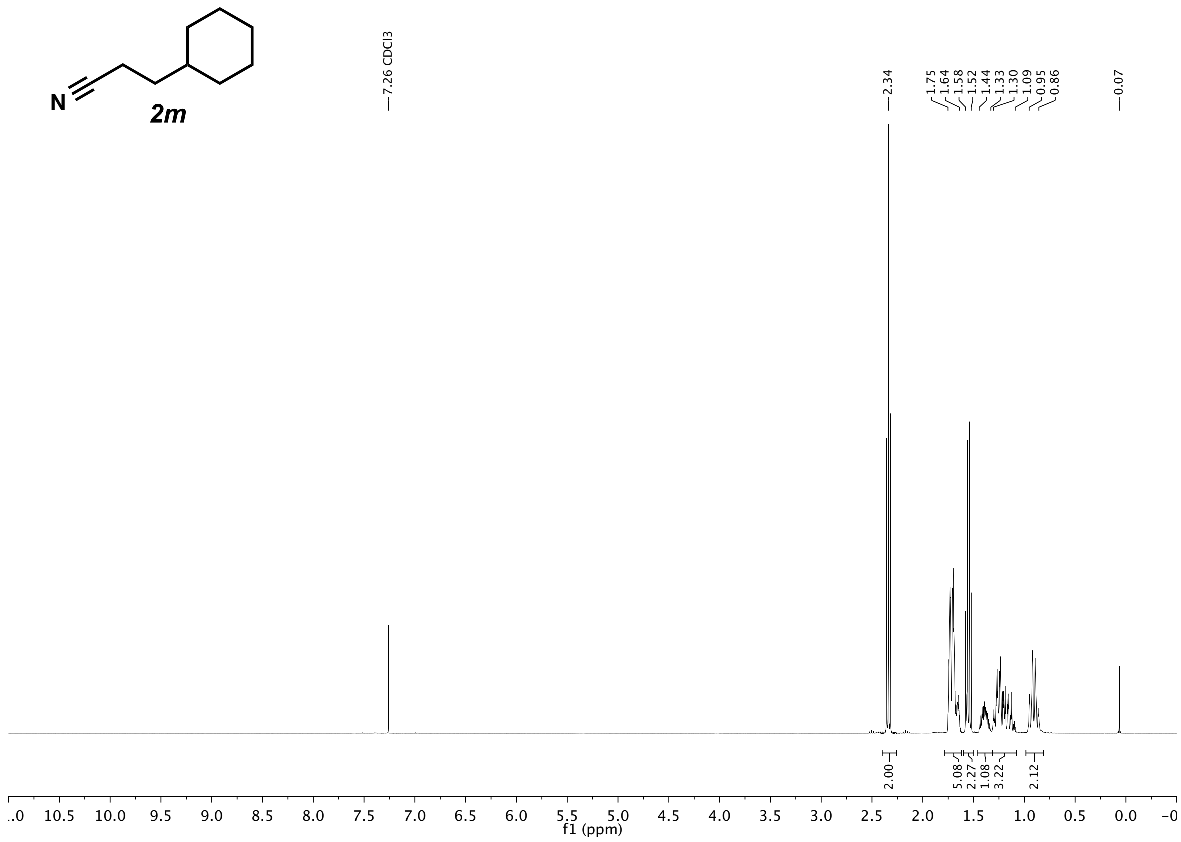

^{1}H NMR (400 MHz, $CDCl_3$) spectrum of compound **2m**.

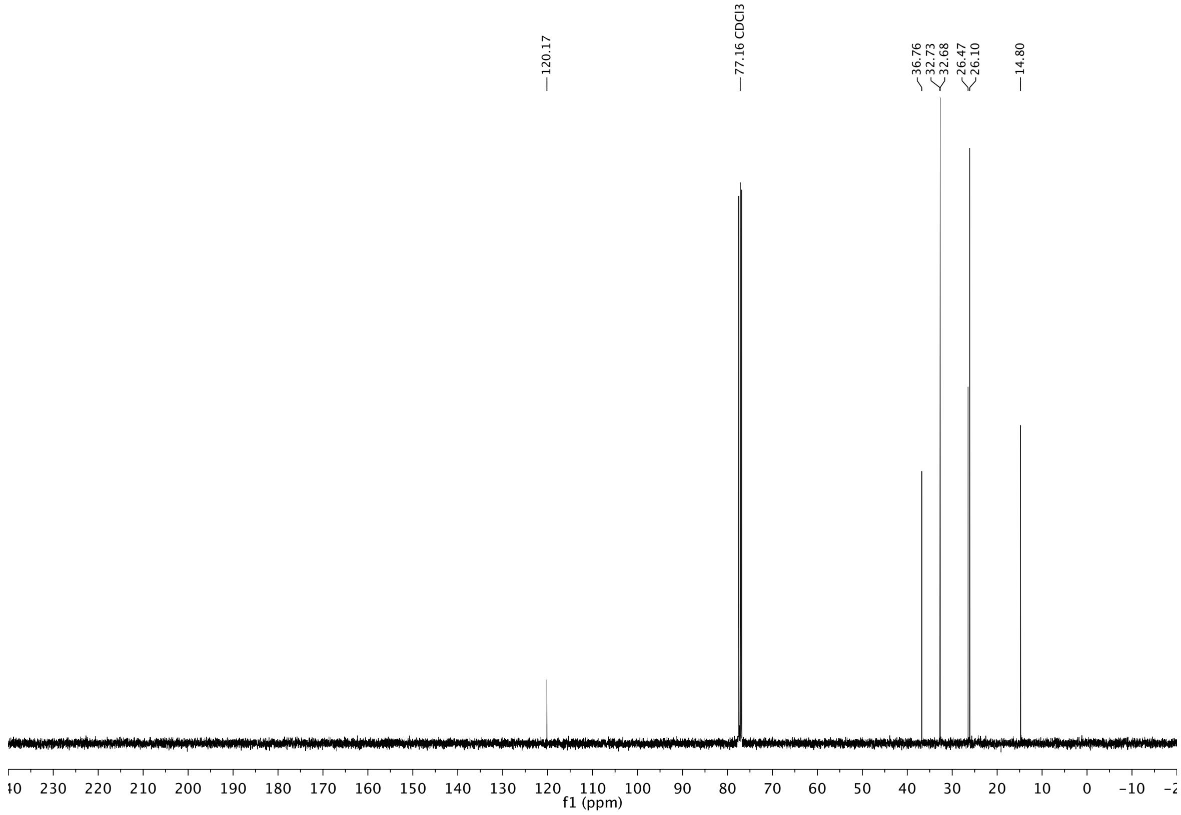

^{13}C NMR (101 MHz, $CDCl_3$) spectrum of compound **2m**.

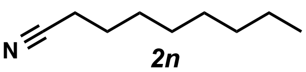

2n

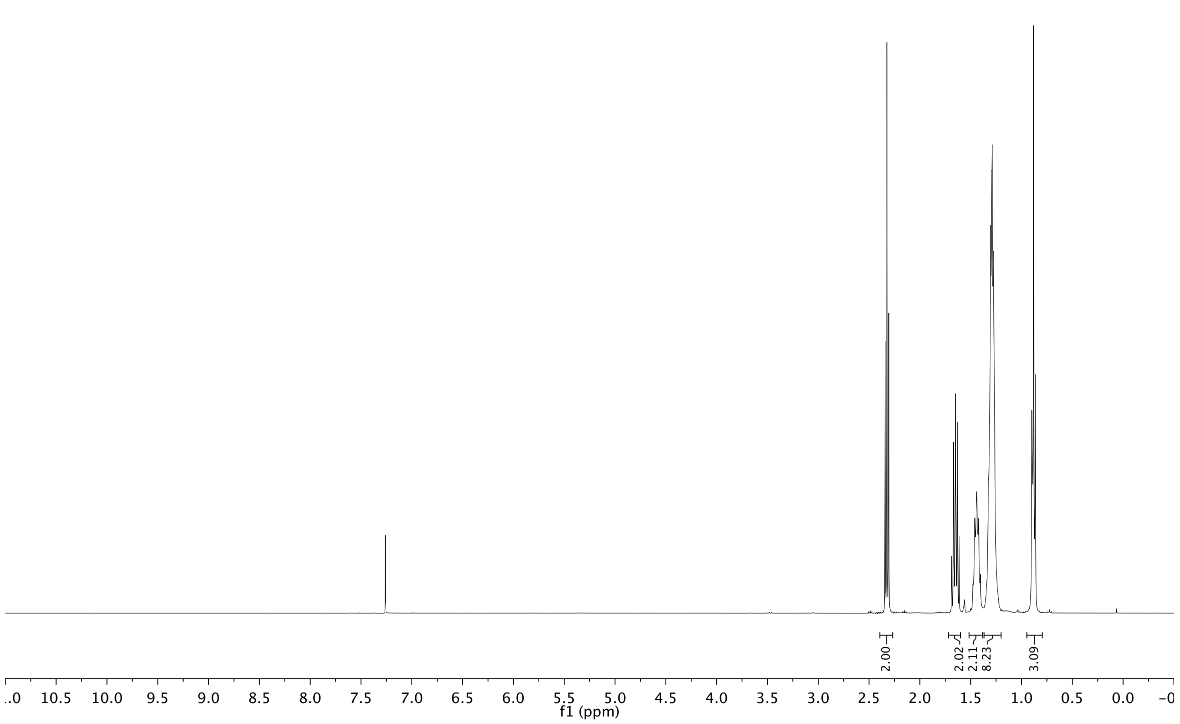

^{1}H NMR (400 MHz, $CDCl_3$) spectrum of compound **2n**.

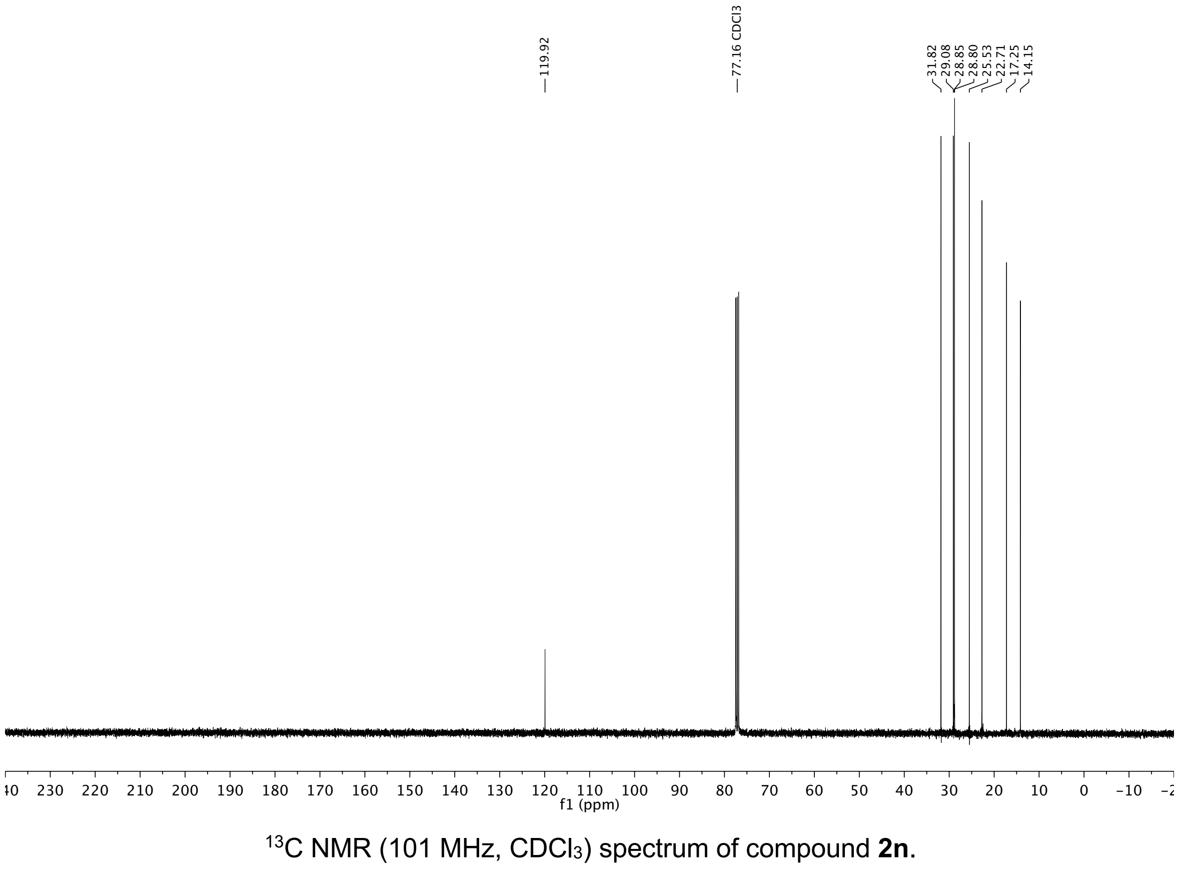

^{13}C NMR (101 MHz, $CDCl_3$) spectrum of compound **2n**.

2o

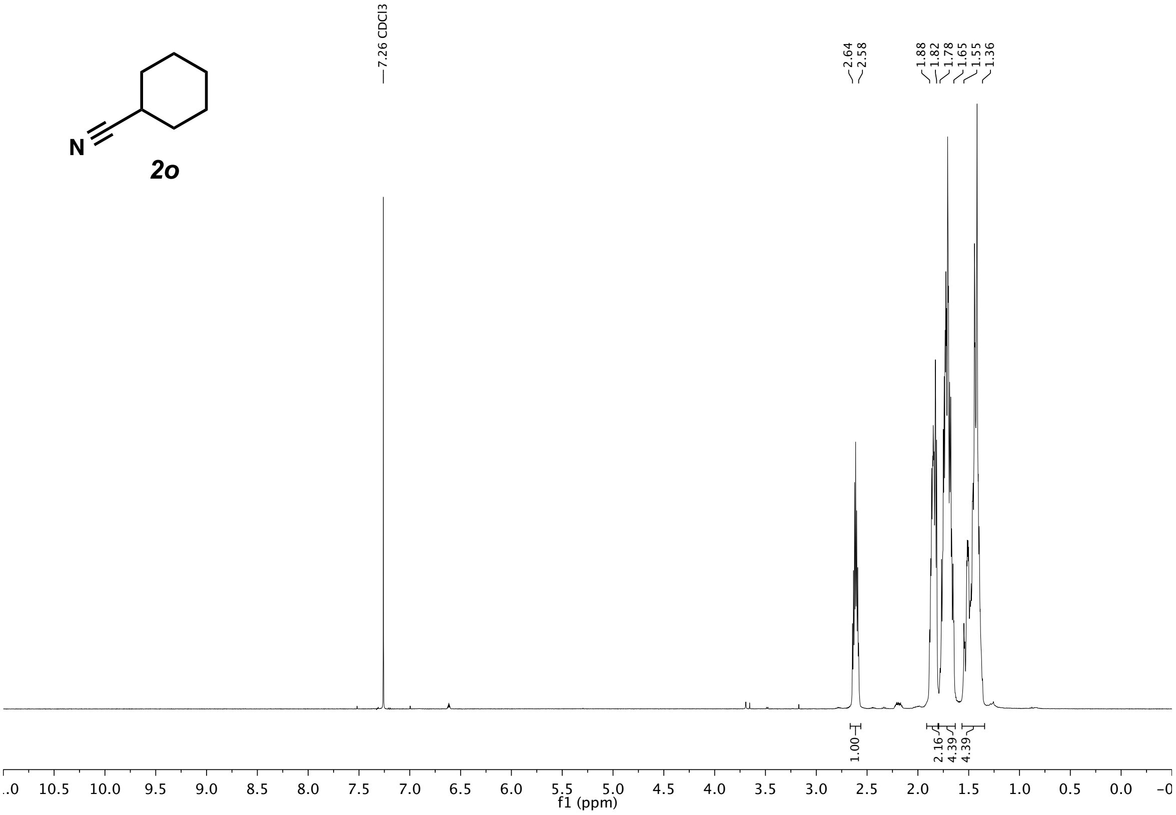

^{1}H NMR (400 MHz, $CDCl_3$) spectrum of compound **2o**.

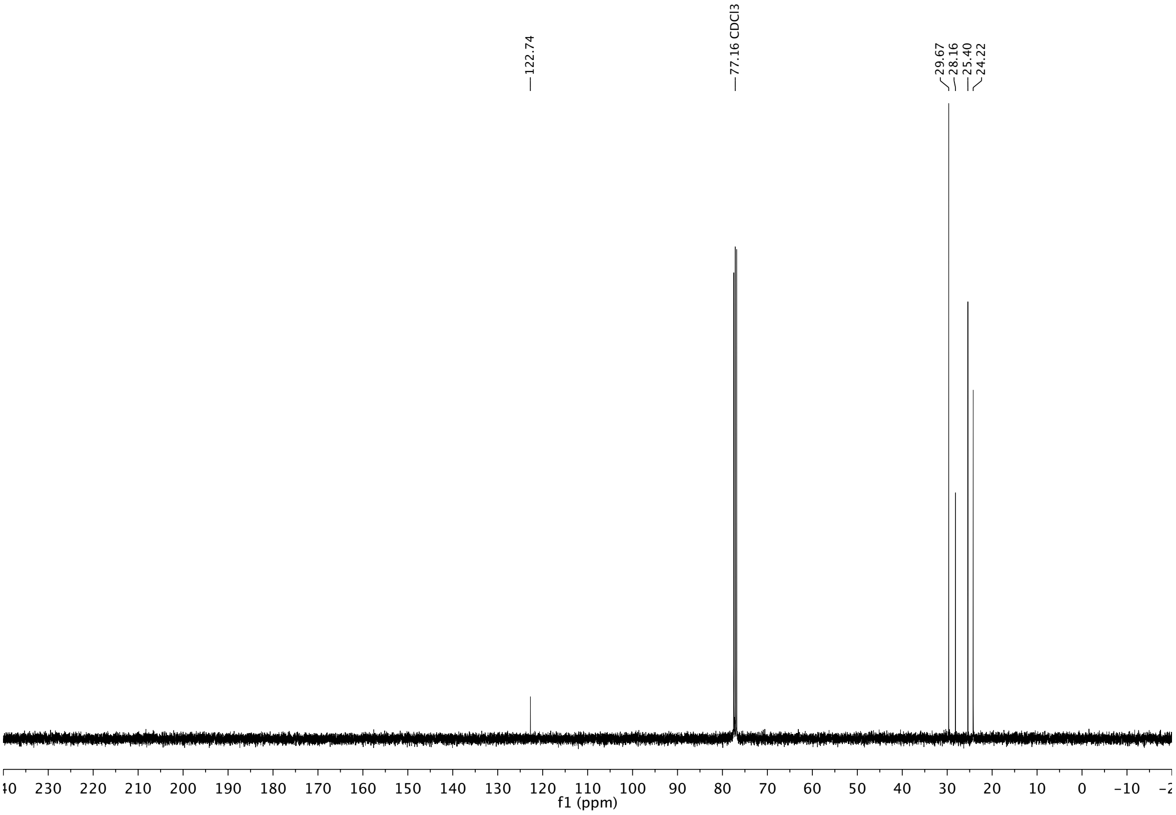

^{13}C NMR (101 MHz, $CDCl_3$) spectrum of compound **2o**.

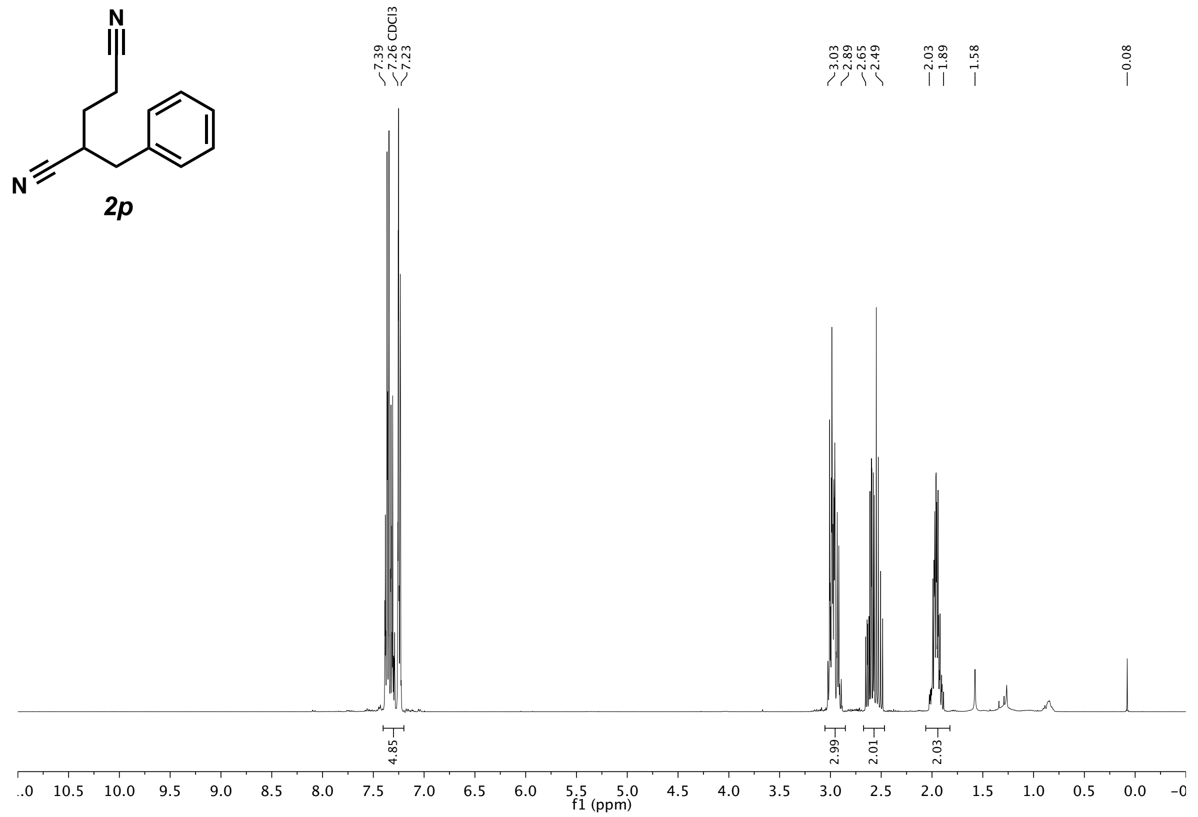

^{1}H NMR (400 MHz, $CDCl_3$) spectrum of compound **2p**.

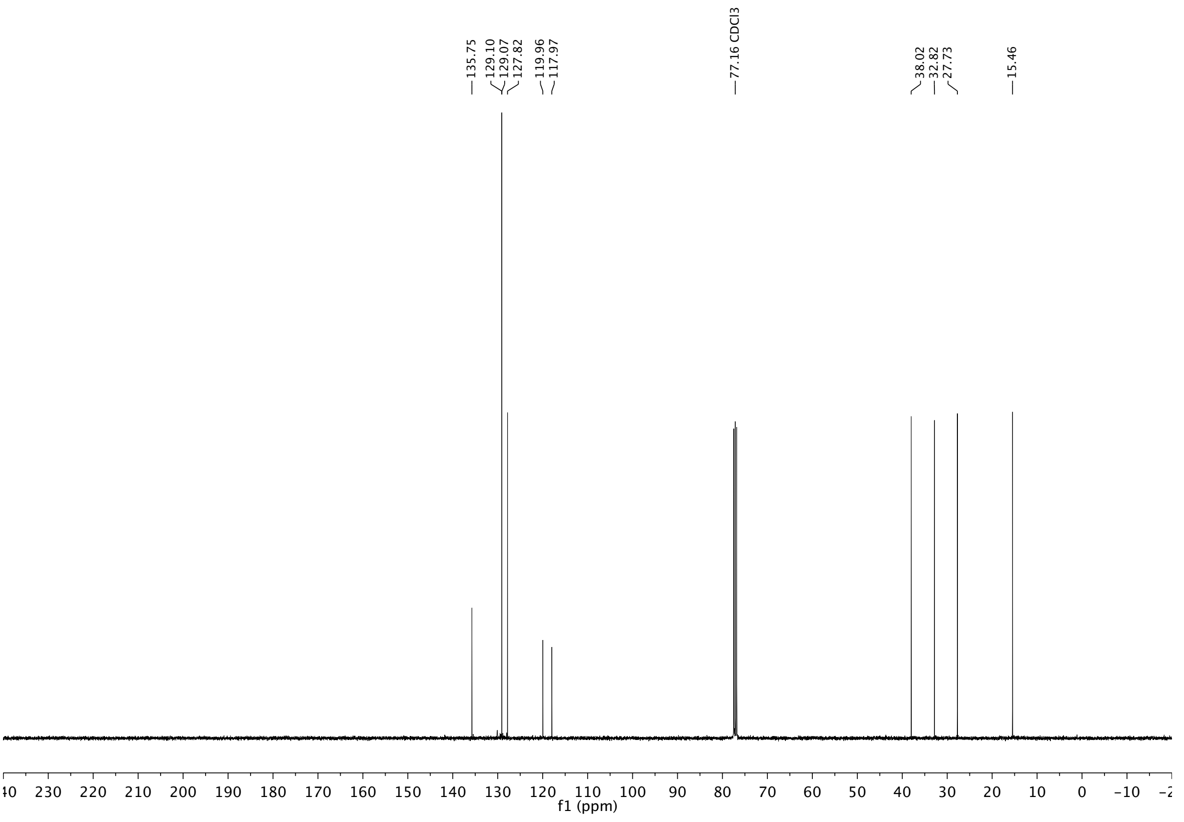

^{13}C NMR (101 MHz, $CDCl_3$) spectrum of compound **2p**.

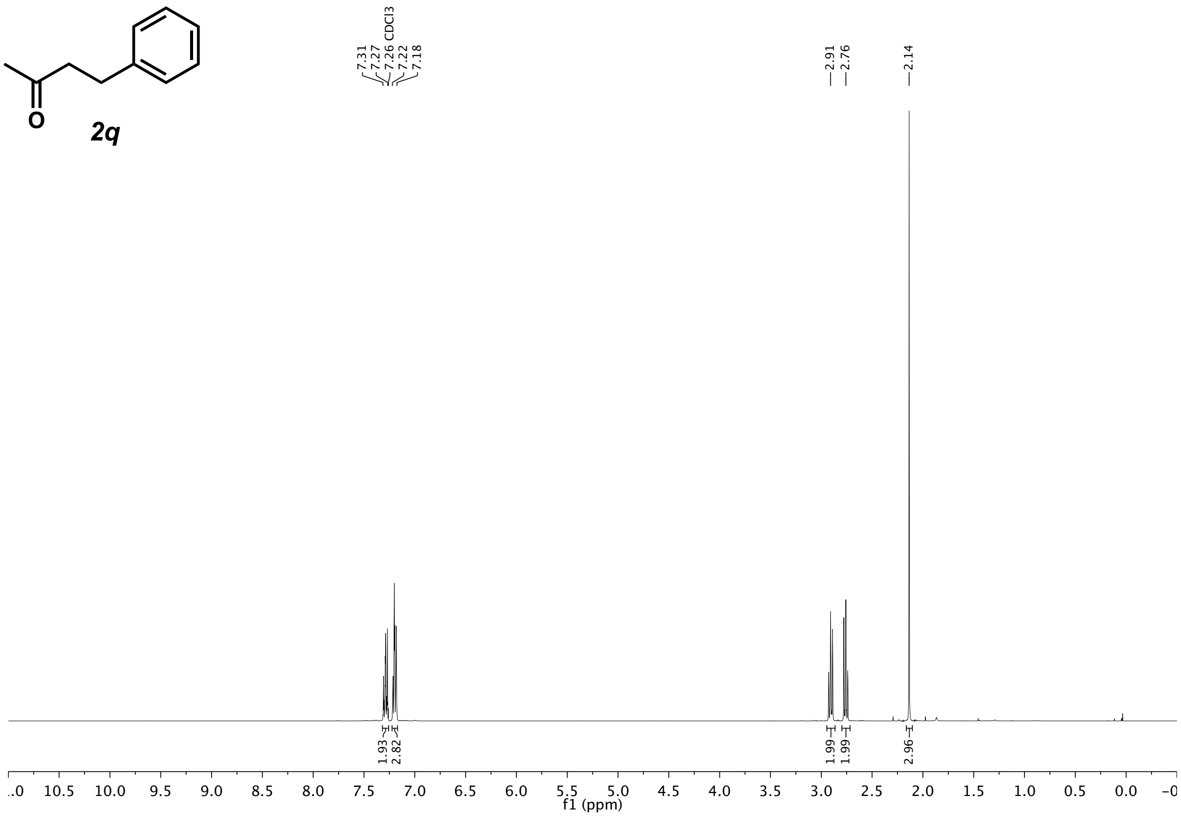

^{1}H NMR (400 MHz, $CDCl_3$) spectrum of compound **2q**.

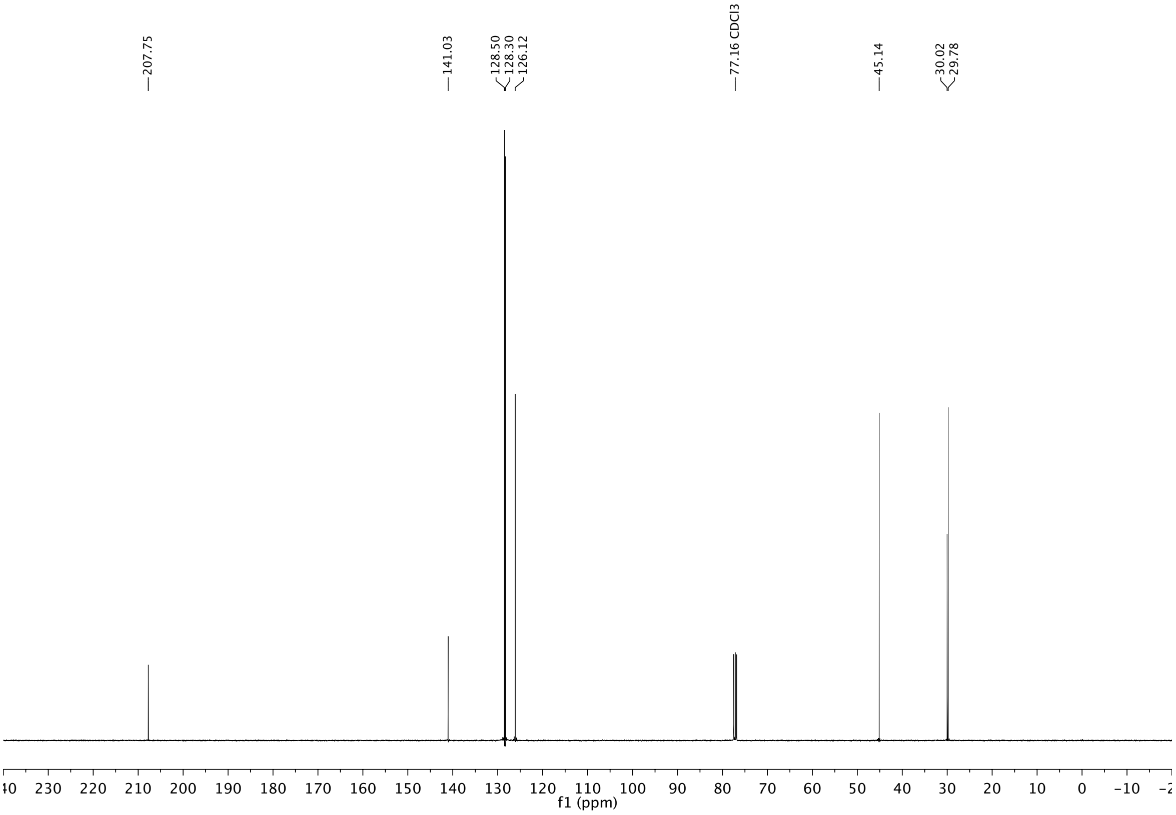

^{13}C NMR (101 MHz, $CDCl_3$) spectrum of compound **2q**.

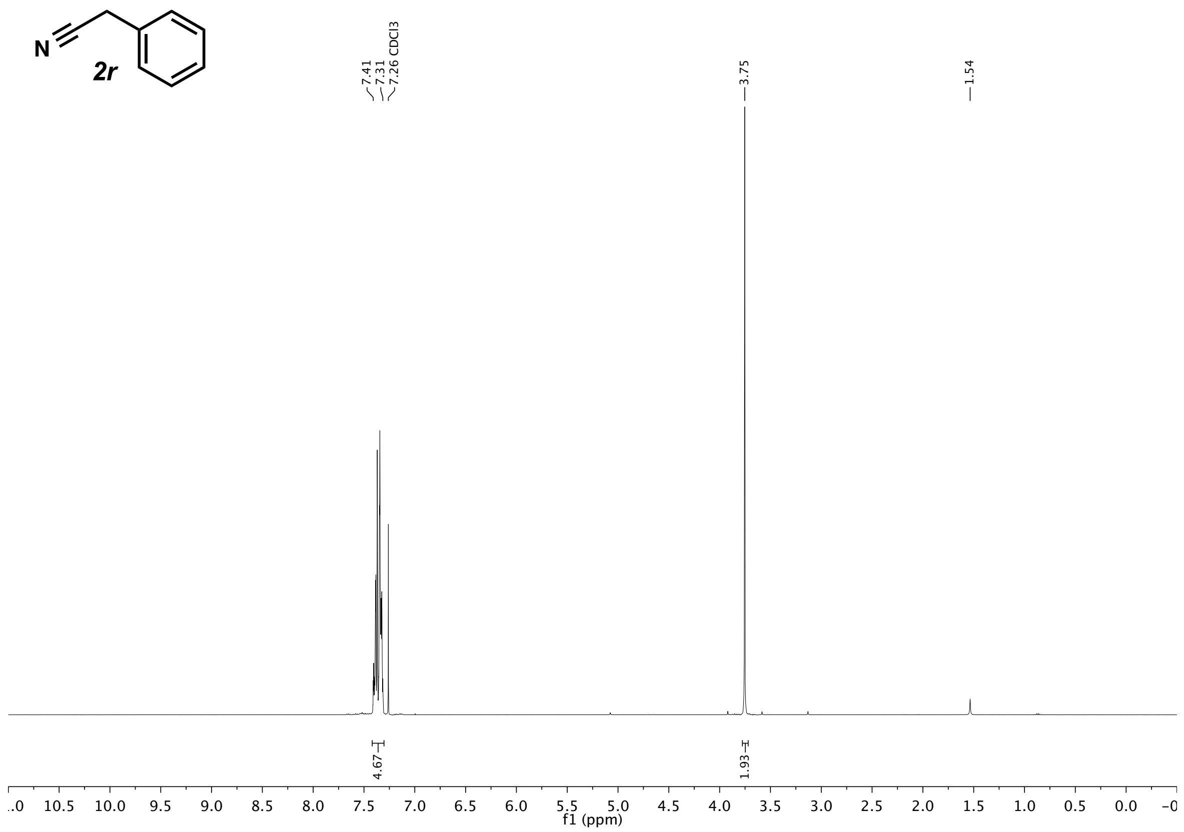

^{1}H NMR (400 MHz, $CDCl_3$) spectrum of compound **2r**.

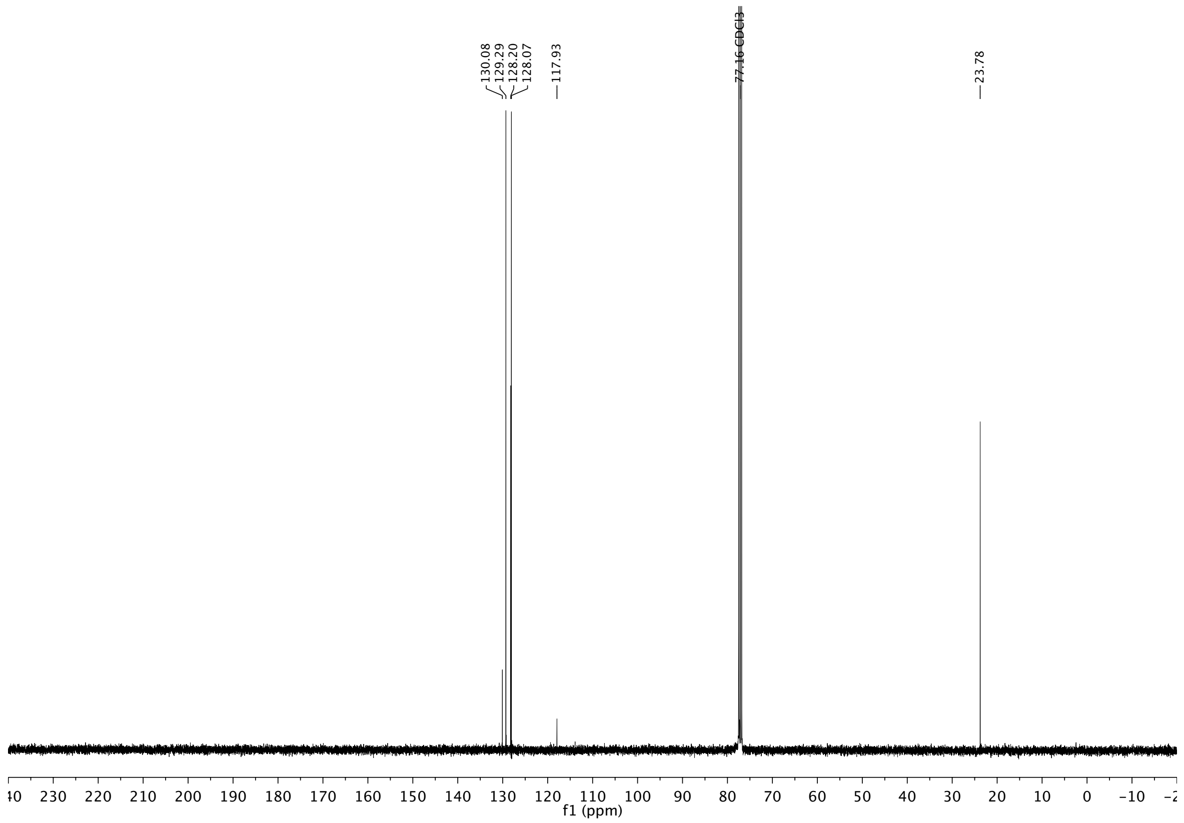

^{13}C NMR (101 MHz, $CDCl_3$) spectrum of compound **2r**.
